# Mechanistic Investigations into the Selective Reduction of Oxygen by a Multicopper Oxidase T3 Site-Inspired Dicopper Complex

**DOI:** 10.1021/acscatal.3c01143

**Published:** 2023-04-12

**Authors:** Phebe H. van Langevelde, Errikos Kounalis, Lars Killian, Emily C. Monkcom, Daniël L. J. Broere, Dennis G. H. Hetterscheid

**Affiliations:** † Leiden Institute of Chemistry, Leiden University, 2300 RA Leiden, The Netherlands; ‡ Organic Chemistry and Catalysis, Institute for Sustainable and Circular Chemistry, Faculty of Science, Utrecht University, Universiteitsweg 99, 3584 CG Utrecht, The Netherlands

**Keywords:** oxygen reduction reaction, dinuclear copper complex, homogeneous electrocatalysis, multicopper oxidases, bio-inspired catalysis

## Abstract

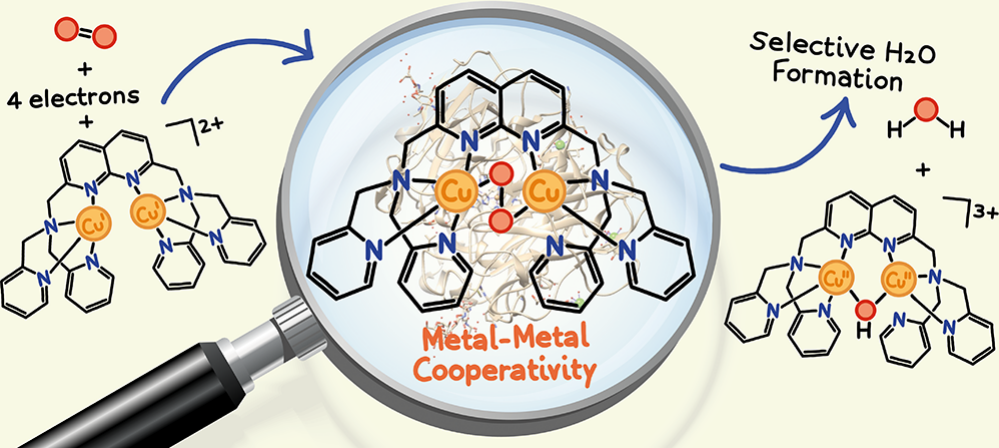

Understanding how multicopper oxidases (MCOs) reduce oxygen in the trinuclear copper cluster (TNC) is of great importance for development of catalysts for the oxygen reduction reaction (ORR). Herein, we report a mechanistic investigation into the ORR activity of the dinuclear copper complex **[Cu_2_L(μ-OH)]^3+^** (**L** = 2,7-bis[bis(2-pyridylmethyl)aminomethyl]-1,8-naphthyridine). This complex is inspired by the dinuclear T3 site found in the MCO active site and confines the Cu centers in a rigid scaffold. We show that the electrochemical reduction of **[Cu_2_L(μ-OH)]^3+^** follows a proton-coupled electron transfer pathway and requires a larger overpotential due to the presence of the Cu-OH-Cu motif. In addition, we provide evidence that metal–metal cooperativity takes place during catalysis that is facilitated by the constraints of the rigid ligand framework, by identification of key intermediates along the catalytic cycle of **[Cu_2_L(μ-OH)]^3+^**. Electrochemical studies show that the mechanisms of the ORR and hydrogen peroxide reduction reaction found for **[Cu_2_L(μ-OH)]^3+^** differ from the ones found for analogous mononuclear copper catalysts. In addition, the metal–metal cooperativity results in an improved selectivity for the four-electron ORR of more than 70% because reaction intermediates can be stabilized better between both copper centers. Overall, the mechanism of the **[Cu_2_L(μ-OH)]^3+^**-catalyzed ORR in this work contributes to the understanding of how the cooperative function of multiple metals in close proximity can affect ORR activity and selectivity.

## Introduction

The four-electron reduction of oxygen to water plays a key role in fuel cell technology.^1,2^ Currently, the most efficient catalysts for the oxygen reduction reaction (ORR) are Pt-based, which drives up the costs of fuel cell devices and limits their commercial viability.^3−5^ Nature has found an efficient way to reduce oxygen in multicopper oxidases (MCOs) using earth-abundant copper.^6^ The thermodynamic and kinetic properties of the ORR catalyzed by the MCO enzyme laccase have been compared to those of Pt electrodes.^7−9^ Interestingly, immobilization of laccase on electrodes resulted in the selective four-electron reduction of oxygen to water at a lower overpotential than Pt.^7^ From this, it is apparent that MCOs, especially laccases, are excellent ORR catalysts. Hence, studying catalysts that are inspired by the MCO active site can point scientists toward new design principles for the next-generation ORR catalysts.

**Figure 1:**
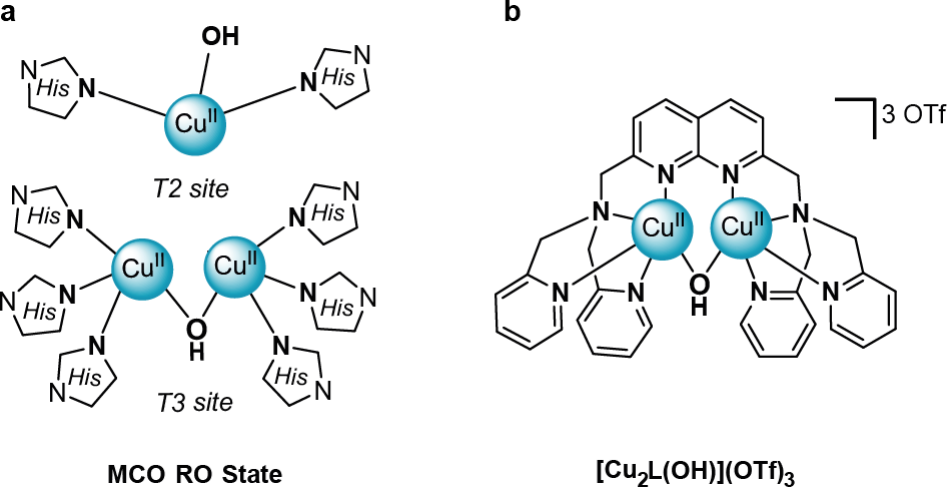
Schematic overview of (a) the MCO active site in the RO state with histidines simplified for clarity and (b) structure of the **[Cu_2_L(μ-OH)](OTf)_3_** complex.

**Figure 2:**
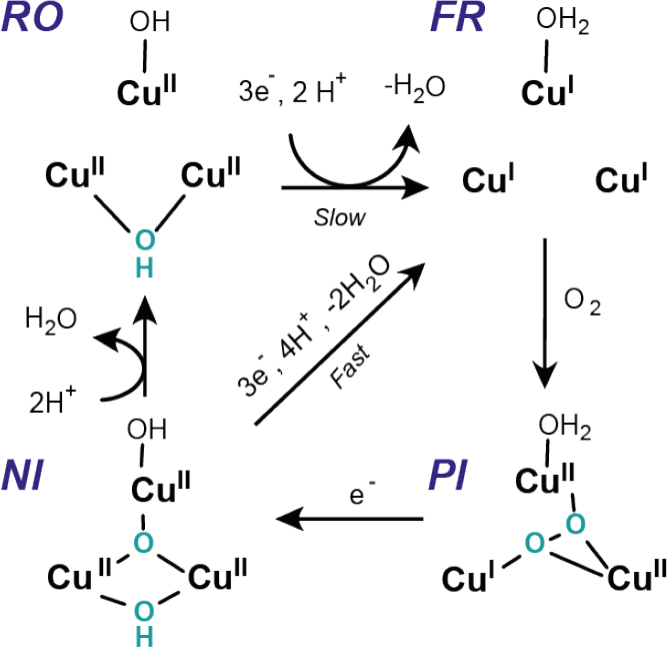
Schematic overview of the oxygen reduction mechanism in the TNC of MCOs.

MCOs are a class of enzymes that couple the oxidation of a variety of substrates to the reduction of oxygen.^6^ The MCO active site consists of a mononuclear T1 copper site and a trinuclear copper cluster (TNC) containing bimetallic T3 and mononuclear T2 sites (Figure 1a).^10^ The T1 site is responsible for the oxidation of substrates and the transfer of electrons to the TNC, where in turn, oxygen can bind and is reduced. A combination of spectroscopic and crystallographic studies, supported by computational studies in more recent years, have contributed to the identification of the key intermediates in the catalytic cycle.^11,12^ In addition, an overall mechanism for the four-electron reduction of oxygen was formulated involving these intermediates shown in Figure 2.^11−14^

The O_2_ reduction mechanism to H_2_O is composed of two distinct two-electron transfers. The first two electrons are transferred when dioxygen binds to the fully reduced (FR) state. In studies using MCOs without T1 sites or with T1 sites replaced by Hg^2+^, the peroxide intermediate (PI), formed after this initial electron transfer, could be detected.^15−17^ In MCOs with an intact T1 site, the PI cannot be isolated since a rapid, second two-electron transfer will lead to cleavage of the O–O bond and the formation of the native intermediate (NI) state.^17^ To complete the catalytic cycle, the NI is re-reduced to the FR state in a process involving four protons and three electrons.^18^ Outside of this catalytic cycle lies the enzyme’s resting oxidized (RO) state, to which the NI will slowly decay in the absence of reducing equivalents.^19,20^ Apparent from this mechanism is that in all catalytic intermediates, except the FR, oxygen-derived species bound between multiple copper centers play a key role. The fact that metal–metal cooperativity plays an essential role throughout the MCO mechanism inspired us to investigate how the ORR activity and selectivity are established at a dinuclear copper site that is inspired by the MCO T3 site.

The MCO active site has inspired the design of many molecular copper complexes to date, typically supported by polynucleating ligands that feature multiple N-donor atoms that mimic the histidine ligands in the TNC.^21,22^ The coordination chemistry and the reactivity of Cu(I) complexes with oxygen has also been extensively studied.^21−23^ In addition, trinuclear copper complexes have been developed to study the role of proton-coupled electron transfers in MCOs.^24^ However, when it comes to the ORR reactivity, only a few complexes with multiple metal centers have been investigated to date. Moreover, the catalytic activity of these complexes has often been investigated in organic solvents and chemical reducing agents are typically used.^25−29^ These reaction conditions do not resemble those in which the ORR activity of MCOs has been studied.

On the other hand, electrochemical studies that have reported the ORR activity of homogeneous metal complexes with two^30−32^ or even three^31,33,34^ copper atoms in aqueous solutions all lack evidence that direct cooperativity between the copper centers takes place during catalysis. A reason for this is that covalently linking copper sites will not invariably result in synergy between the metal centers. For instance, not all metal centers might participate during catalysis,^31^ the copper sites can turn away from each other,^30^ the catalyst can decompose during electrocatalysis,^32^ or multiple copper centers in close proximity will result in a change of selectivity, but not due to direct metal–metal cooperativity.^33^ An exception is a study by Yu and co-workers, in which three copper centers are forced closely together, although a full characterization of the active species and a catalytic mechanism are absent.^34^

To achieve catalytic ORR in a cooperative manner, we set out to study the ORR activity of a complex with a rigid ligand framework that can coordinate two copper atoms in close proximity. Recently, the mononuclear Cu(tmpa) complex (tmpa = tris(2-pyridylmethyl)amine) was studied in one of our groups and was shown to catalyze the ORR with the highest TOF reported for any homogeneous copper complex.^35^ Interestingly, the dinuclear analogue of tmpa, the 2,7-bis[bis(2-pyridylmethyl)aminomethyl]-1,8-naphthyridine (**BPMAN**) ligand, can coordinate two copper atoms in close proximity to form the **[Cu_2_L(μ-OH)]^3+^** (**L** = **BPMAN**) complex^36^ and was previously reported to electrochemically catalyze the water oxidation reaction (Figure 1b).^37^ It is evident from Figure 1 that **[Cu_2_L(μ-OH)]^3+^** bears similarities to the T3 site in MCOs, including the μ-OH ligand. Moreover, the Cu–Cu distance in **[Cu_2_L(μ-OH)]^3+^** was reported to be 3.4 Å,^37^ which is similar to the reported Cu–Cu distance in the T3 site of MCOs in the RO state of approximately 3.7 Å.^12^

In this study, we present a mechanistic investigation of the ORR catalyzed by **[Cu_2_L(μ-OH)]^3+^** and explain its activity and selectivity, which is supported by a combination of electrochemical and reactivity studies to identify the nature of the active species. In addition, by comparing our results to the previously reported mononuclear Cu(tmpa) system, the effect of the dinuclear copper core on the reaction kinetics and selectivity was explored. In this way, the study of this T3 site-inspired complex contributes to a better understanding of how the cooperative function of multiple metals in close proximity can affect ORR activity and selectivity.

## Results and Discussion

### Synthesis of **[Cu_2_L(μ-OH)]OTf_3_**

The 2,7-bis[bis(2-pyridylmethyl)aminomethyl]-1,8-naphthyridine ligand was synthesized in 64% yield using a new synthetic protocol (see the Supporting Information). With the **BPMAN** ligand (**L**) in hand, crystalline **[BPMANCu_2_(μ-OH)](OTf)_3_** (**[Cu_2_L(μ-OH)]OTf_3_**) was prepared in a reaction with Cu(OTf)_2_ in acetonitrile according to literature procedures.^37^ The paramagnetic ^1^H-NMR spectrum of **[Cu_2_L(μ-OH)]^3+^** in D_2_O, not reported previously, shows a set of 7 and a set of 2 of equally integrating resonances between +71 and +8 ppm with a relative ratio of 2:1 (see Figure S3). This observation can be explained by the methylene protons flanking the pendant pyridines being magnetically inequivalent (set of 2) and agrees with the expected resonances of the four pyridine arms and the 1,8-naphthyridine moiety. The surprisingly sharp peaks in the paramagnetic ^1^H-NMR spectrum of this dicopper(II) compound are likely the result of antiferromagnetic coupling between the two Cu(II) centers, as has been observed for other hydroxo-bridged dicopper(II) complexes.^38,39^ The paramagnetic ^1^H-NMR spectrum of the same compound in DCM-*d*_2_ gives rise to a similar spectrum, except for an additional broad resonance at −70.8 ppm (see Figure S5). We assign this resonance to the hydroxide proton and attribute the absence of this resonance in D_2_O to the rapid exchange of the proton with the solvent.

### Cyclic Voltammetry of **[Cu_2_L(μ-OH)]^3+^**

**Figure 3:**
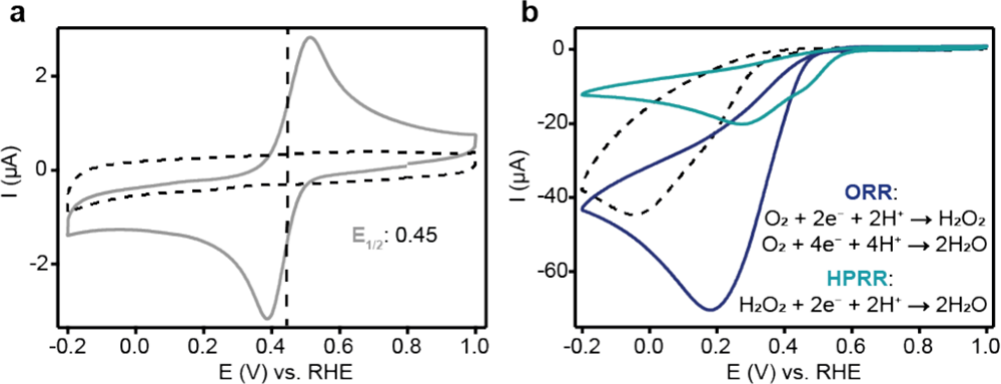
CVs of (a) the Cu^II^/Cu^I^ redox couple of **[Cu_2_L(μ-OH)]^3+^** with a half-wave potential of 0.45 V vs RHE indicated by the vertical line and the bare GC electrode under an Ar atmosphere by a black dashed line and (b) the ORR (dark blue) and HPRR (light blue) catalyzed by **[Cu_2_L(μ-OH)]^3+^**. ORR activity of the bare GC electrode shown as reference (black dashed line). Conditions: 0.15 mM **[Cu_2_L(μ-OH)]^3+^**, 0.1 M PB pH 7, Ar or O_2_ atmosphere or 1.1 mM H_2_O_2_, 293 K, 100 mV/s scan rate.

The **[Cu_2_L(μ-OH)]^3+^** complex was studied electrochemically in a 0.1 M phosphate buffer (PB) at pH 7 using a glassy carbon (GC) working electrode. A cyclic voltammogram (CV) under an Ar atmosphere shows a single Cu^II^/Cu^I^ redox couple with a half-wave potential (*E*_1/2_) of 0.45 V vs RHE (Figure 3a). The redox couple of **[Cu_2_L(μ-OH)]^3+^** is shifted toward higher potentials compared to the mono-nuclear analogue Cu(tmpa) that has an *E*_1/2_ of 0.21 V vs RHE under the same conditions.^35^ To investigate if **[Cu_2_L(μ-OH)]^3+^** displays ORR activity, a CV in the presence of oxygen was recorded. Indeed, the CV showed a clear catalytic wave (Figure 3b). In addition to the ORR, the catalytic activity of **[Cu_2_L(μ-OH)]^3+^** for the hydrogen peroxide reduction reaction (HPRR) is of interest because H_2_O_2_ is found as a detectable intermediate in the aqueous electrochemical ORR mechanism of other copper complexes with tetradentate pyridylamine ligands that is formed after the two-electron reduction of oxygen.^30,33,35,40,41^ In the presence of 1.1 mM H_2_O_2_, which is the same concentration as the maximum solubility of oxygen in PB, a catalytic wave was observed with a slightly earlier onset than the ORR (Figure 3b). As this earlier onset is only present in the first scan, we contribute this occurrence to the weak binding of H_2_O_2_ to the oxidized form of the catalyst, although such a species could not be detected by other techniques like NMR. As the time between the first and second scan is relatively short, this equilibrium is not restored in between cycles and the onset shifts back to that of the ORR in subsequent scans (see Figure S8). The peak-shaped waves in both the ORR and HPRR voltammograms indicate the depletion of substrate during catalysis. The same mass transfer phenomena are observed in rotating disk electrode experiments, vide infra. We attribute the difference in the peak catalytic currents between the ORR and HPRR to the different diffusion rates of O_2_ (1.9 × 10^–5^ cm^2^ s^–1^) and H_2_O_2_ (0.8–1.4 × 10^–5^ cm^2^ s^–1^)^42,43^ and possible difference in electron transfer number for each of the respective reduction reactions.

Prior to further mechanistic studies, we carefully investigated if the electrochemical data is representative of the homogeneous catalyst in solution, rather than any heterogeneous species that may have been deposited on the electrode surface. Such investigation on the nature and stability of the active species is important in homogeneous electrocatalysis.^44^ This has been exemplified by a previous study by one of our groups on a different dicopper ORR catalyst used as a model system for MCOs, for which the sole active species in ORR was found to be copper deposit.^32^ In addition, catalytic rates and selectivities can only be assigned to the homogeneous catalyst in solution if the interference of any formed heterogeneous deposits can be minimized.

The possible formation of any deposited species was studied in a combination of CV experiments and electrochemical quartz crystal microbalance (EQCM) experiments (see the Supporting Information Sections 4.1 and 4.2). First, we investigated if any species deposits onto the electrode during CV measurements by recording a CV in a solution of **[Cu_2_L(μ-OH)]^3+^** and subsequently recording a CV of the same electrode in a solution in the absence of catalyst. These experiments under an Ar atmosphere show that the **[Cu_2_L(μ-OH)]^3+^** catalyst tends to adsorb onto the glassy carbon electrode during CV measurements, but it is likely to stay intact as the redox couple of the species that adsorbs onto the electrode has the same *E*_1/2_ as the **[Cu_2_L(μ-OH)]^3+^** redox couple (see Figure S9a and Figure S10a). In contrast, the same experiments in the presence of O_2_ or H_2_O_2_ show that a new heterogeneous species forms on the electrode during catalysis that is also catalytically active for the ORR and HPRR (see Figure S9). This indicates that the catalyst partly decomposes during catalysis. Furthermore, EQCM measurements were carried out to investigate the possible mass change of the electrode during CV experiments as this is an indication that heterogeneous deposits form on the surface of the electrode. In line with the CV experiments discussed above, EQCM experiments indeed show that a heterogeneous species is formed on the surface of the electrode during catalysis, whereas this formation is prevented if the **[Cu_2_L(μ-OH)]^3+^** catalyst is studied under an Ar atmosphere (see Figure S13).

Next, we investigated to what extent the adsorption of catalyst or the formation of a deposit on the electrode surface contribute to the recorded activity of **[Cu_2_L(μ-OH)]^3+^**. In measurements under an Ar atmosphere the adsorbed species behaves similar to the homogeneous one, and upon changing to an oxygen atmosphere, its catalytic activity is small as only a little amount of catalyst adsorbs onto the electrode (see Figure S10 and Figure S9a). Therefore, it was excluded that any adsorbed catalyst interferes with the measurements of the homogeneous **[Cu_2_L(μ-OH)]^3+^** species under an Ar atmosphere.

To study the catalytic activity of the deposit that forms during catalysis by decomposition of **[Cu_2_L(μ-OH)]^3+^**, additional measurements were carried out under non-substrate limited conditions. These conditions allow to study the catalytic activity, while this is not governed by the depletion of oxygen. From these measurements, it is clear that the catalytic activity of **[Cu_2_L(μ-OH)]^3+^** in the first scan of a CV measurement is significantly less than the activity of the deposits that form over the course of several scans (see Figure S11a). In other words, under catalytic conditions, active deposits form over the course of multiple CV scan. To verify that the largest part of the activity can still be contributed to the homogeneous **[Cu_2_L(μ-OH)]^3+^** species, we investigated the activity of the deposit that forms only during the first part of the catalytic CV measurement (see Figure S12). This indicates that the contribution of the deposit to the catalytic current is minimal, which is further supported by the EQCM measurements that show that the buildup of material on the electrode predominantly occurs below 0.2 V, when the ORR has already peaked under stationary conditions.

To ensure that the influence of any heterogeneous deposits on the results of our studies into the activity of homogeneous **[Cu_2_L(μ-OH)]^3+^** is kept to a minimum, in this work, only the first CV scans recorded on a freshly polished electrode were used. In addition, this study focusses solely on the fundamental catalytic mechanism of the catalyst, not on any long-term activity, since in that case, the influence of heterogeneous deposits cannot be ruled out.

### Redox Behavior of **[Cu_2_L(μ-OH)]^3+^**

The first step in the catalytic ORR mechanism is the reduction of **[Cu_2_L(μ-OH)]^3+^** to obtain the active reduced catalyst. The redox behavior of **[Cu_2_L(μ-OH)]^3+^** was previously investigated by Zhang *et al*. in 0.1 M PB of pH 7 using a boron-doped diamond (BDD) working electrode.^37^ They reported two irreversible waves for the reduction and oxidation of the catalyst with a peak-to-peak separation (Δ*E*_p_) of 340 mV at a scan rate of 5 mV/s. In our study, the Δ*E*_p_ of the Cu^II^/Cu^I^ redox couple at a scan rate of 100 mV/s reduces to 127 mV (Figure 3a). We attribute the smaller Δ*E*_p_ in this study to the more favorable electron transfer properties of GC compared to BDD as BDD is known to act as a semiconductor at low dopant densities.^45^

Scan rate dependence studies of the redox couple (10–500 mV/s) show that the peak current linearly depends on the square root of the scan rate, which indicates a diffusion-controlled process and hence that **[Cu_2_L(μ-OH)]^3+^** is present as a homogeneous species in solution (see Figure S14b). The Δ*E*_p_ of 127 mV at 100 mV/s indicates a quasi-reversible process and suggests that the electron transfer process to reduce and re-oxidize **[Cu_2_L(μ-OH)]^3+^** is slow. In addition, a Laviron plot shows that the peak potential of the redox couple depends linearly on the logarithm of the scan rate (see Figure S14c), which means that the electron transfer processes in the catalyst become relatively slow compared to the timescale of the CV experiment. A third sign of slow electron transfer kinetics is the relatively low half-wave potential of the catalytic wave (*E*_cat/2_) in the ORR, and thus, *E*_cat/2_ < *E*_1/2_ holds for **[Cu_2_L(μ-OH)]^3+^**, while for fast catalysts, it is expected that *E*_cat/2_ = *E*_1/2_ or even *E*_cat/2_ > *E*_1/2_ when substrate depletion plays a role.

In MCOs, both copper centers in the T3 site of the RO state are reduced simultaneously in a proton-coupled electron transfer (PCET) step to form the FR state, resulting in the loss of the μ-hydroxyl ligand as H_2_O.^6,11^ In this study, only a single peak for the Cu^II^/Cu^I^ redox couple is observed, which indicates that in **[Cu_2_L(μ-OH)]^3+^**, both copper atoms are reduced and oxidized simultaneously. To see whether the reduction of **[Cu_2_L(μ-OH)]^3+^** also involves a protonation, we studied the influence of the proton concentration on the redox behavior of the complex (see the Supporting Information Section 6). CVs of **[Cu_2_L(μ-OH)]^3+^** under an Ar atmosphere were recorded in a 0.01 M Britton–Robinson buffer over a wide pH range. Attempts to study the pH dependence in unbuffered solution resulted in difficulties in obtaining reproducible data. We ascribe this observed irregularity to the complex’s propensity to access different protonation states. Particularly, the pyridinic nitrogen atoms of the ligand can be protonated easily as shown computationally by Zhang *et al*.^37^ The use of a buffered solution minimizes local pH changes during experiments.

CV measurements in buffered solution show that the *E*_1/2_ of the **[Cu_2_L(μ-OH)]^3+^** redox couple changes as a function of pH. The slope of this graph is −17 mV/dec, which we could not assign to a specific reaction step involving one or more protons (see Figure S15).

**Figure 4:**
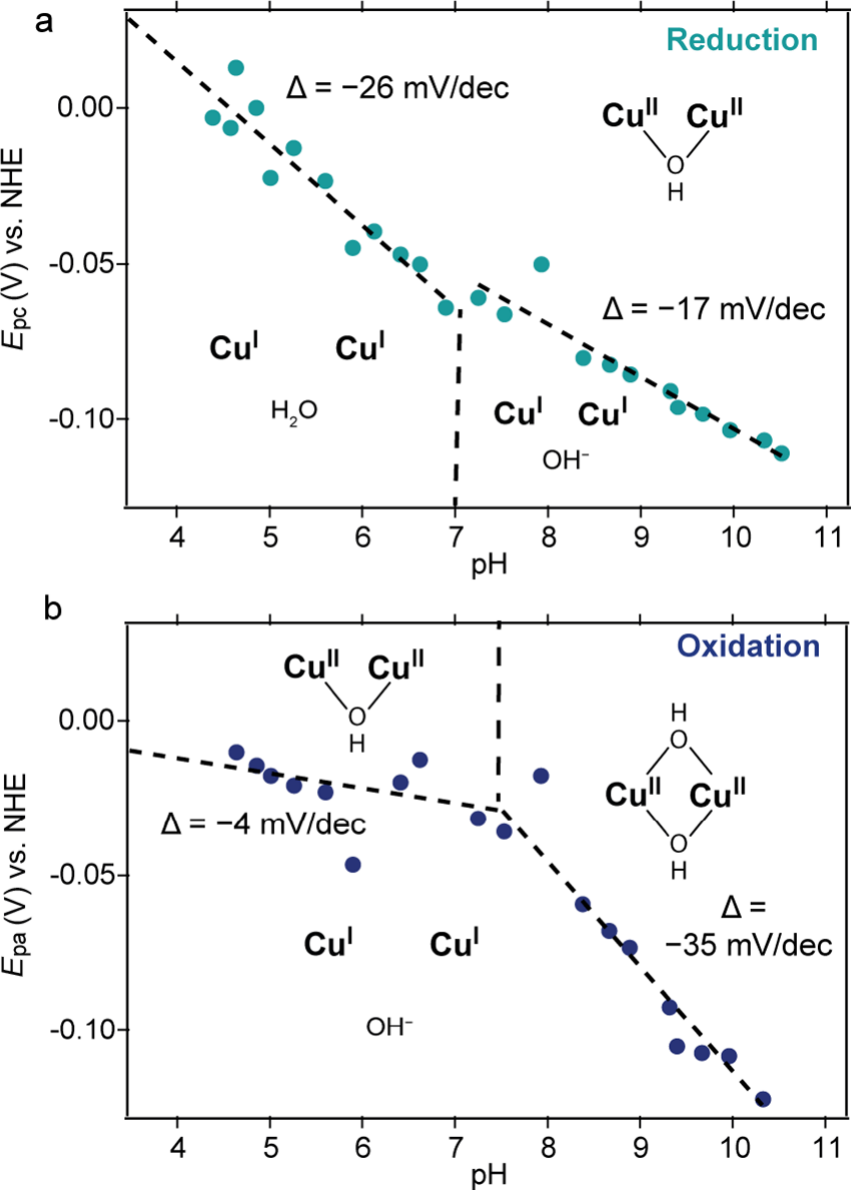
Plot of the peak potential of (a) the cathodic (*E*_pc_) and (b) anodic (*E*_pa_) peaks of **[Cu_2_L(μ-OH)]^3^** from DPV measurements as a function of pH, including the slope of the graph and the different dicopper species involved in the processes. Conditions: 0.15 mM **[Cu_2_L(μ-OH)]**^**3**+^, 0.01 M Britton–Robinson buffer and 0.05 M Na_2_SO_4_, Ar atmosphere, 293 K, DPV recorded with regular voltage pulses of 3 mV, a step potential of 0.3 mV, a modulation time of 3 ms, and a time interval of 50 ms.

More clarity was obtained by analysis of differential pulse voltammetry (DPV) measurements at varying pH, a technique that can deconvolute multiple redox events (see Figure S16). A plot of the peak potential of the cathodic (*E*_pc_) and anodic (*E*_pa_) events as a function of pH shows that both peaks shift in a different manner upon changing the pH (see Figure 4). Between pH 4 and 7, the reductive peak changes with −26 mV/dec. This value is close to the theoretical value of −30 mV/decade of a step in which one proton and two electrons are transferred^46^ and agrees with the protonation of the hydroxyl ligand upon reduction of both copper centers in **[Cu_2_L(μ-OH)]^3+^**. In contrast, Zhang *et al*. measured on BDD a shift of −60 mV/decade of the cathodic wave, which they contributed to the single electron reduction of **[Cu_2_L(μ-OH)]^3+^** and a proton transfer forming a mixed-valence Cu^II^Cu^I^ species.^37^ Our results differ from this observation, likely due to the more favorable electron transfer rates featured by the GC electrode. Above pH 7, a clear kink in the graph can be seen and the cathodic peak shifts with only −17 mV/dec. This points toward a mechanism in which mainly electrons are transferred and suggests that under alkaline conditions, the μ-hydroxyl ligand is not always protonated before it will leave the copper site.

Regarding the anodic peak, we observe a trend that is the opposite of the cathodic peak. At low pH, the position of the oxidation peak does not change, while above pH 7, we observe a slope of −35 mV/dec. At these high pH values, we ascribe this slope to the formation of either a bis-μ-hydroxyl or deprotonated μ-O species.

Interestingly, the oxidative DPV measurement at low pH values gives rise to a second oxidation peak at more positive potentials (see Figure S16b). The presence of a second oxidation peak that only forms at low pH values suggests that a wide availability of protons induces a chemical process. We could not further verify the origin of this second oxidation peak, but we hypothesize that it belongs to oxidation of a slow equilibrium product that forms out of Cu(I)Cu(I).

The redox behavior of **[Cu_2_L(μ-OH)]^3+^** was also studied in acetonitrile solution since precise control over the availability of protons is more easily attainable in organic solvent than in aqueous solution. Interestingly, a solution of 0.3 mM **[Cu_2_L(μ-OH)]^3+^** in 0.1 M TBAPF_6_ in MeCN does not show a clear reduction event, which is not in agreement with the single reduction peak observed in aqueous solutions. The multiple, small reduction peaks observed below −0.5 V vs RHE could not be assigned to a specific redox process and most likely are the result of traces of water that are always present in MeCN. Addition of a proton source (triethylammonium hexafluorophosphate, TEAPF_6_) generates a CV with a clear redox couple and a single reduction wave, demonstrating that in MeCN, a proton source is required for the reduction of **[Cu2L(μ-OH)]^3+^** (Figure 6a). Such behavior is expected in organic solutions as it is likely that upon reduction of the catalyst, formation of H_2_O is preferred over the liberation of OH^–^. This agrees with the observations in aqueous solution below pH 7 and strongly suggests that protonation of the μ-hydroxyl ligand takes place simultaneously with reduction of the catalyst.

### Role of the Bridging Hydroxyl Ligand

Intrigued by the complex redox behavior of **[Cu_2_L(μ-OH)]^3+^**, the coordination chemistry of the Cu^II^-OH-Cu^II^ motif was studied in more detail. To begin with, possible protonation of the μ-hydroxyl ligand was studied in UV–vis measurements. The absorption band at 350 nm gradually disappears when the pH is lowered from 5.8 to 2.8 and reappears again when the pH is increased. Based on the literature, we expect this absorption to be the OH^–^ → Cu(II) LMCT.^47^ We ascribe this change to the protonation of the hydroxide and the reversible formation of a **[Cu_2_L(H_2_O)]^4+^** complex (see Figure S17).

**Figure 5:**
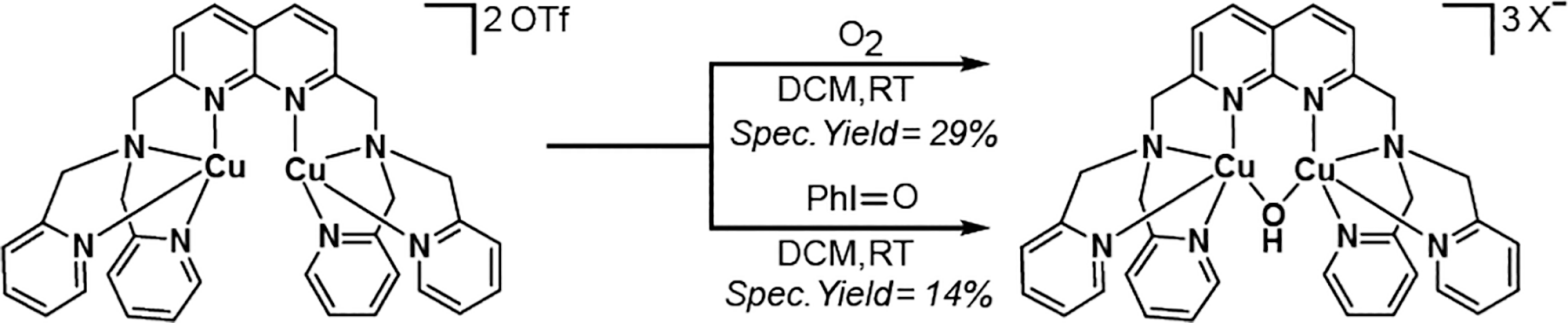
Overview of the reactions taking place when a solution of the **[Cu_2_L](OTf)_2_** complex in DCM is exposed to air or to PhIO, resulting in the formation of the oxidized **[Cu_2_L(μ-OH)]^3+^** cation in low spectroscopic yields.

To further gain insights into the Cu^II^-OH-Cu^II^ motif, we studied how the μ-hydroxyl ligand can form. Attempts to synthesize the [**Cu_2_L](OTf)_4_** complex did not succeed. In the presence of trace amounts of water, the **[Cu_2_L(μ-OH)]^3+^** species will form, which points out that the μ-hydroxyl ligand results in the formation of a very stable Cu(II) species. Therefore, the Cu^I^ complex **[Cu_2_L](OTf)_2_** was synthesized from treatment of BPMAN with 1 equiv of [CuOTf]_2_·toluene in DCM in 97% yield with NMR data agreeing with reported literature values (see the Supporting Information).^36^ To see whether the bridging hydroxyl ligand can form, a solution of **[Cu_2_L]^2+^** in DCM was exposed to air. This resulted in conversion toward a new, green colored species, which gives rise to a set of paramagnetic resonances in the ^1^H-NMR spectrum that match the resonances found for **[Cu_2_L(μ-OH)]^3+^** (Spec. Yield = 29%, see Figure 5 and the Supporting Information Section 7.1). Interestingly, within 2 min of exposing the DCM solution to air, a short-lived (only trace amounts detectable after 1 h) intermediate was observed in the ^1^H-NMR spectrum. We argue that the intermediate that we observe in NMR is not the previously spectroscopically observed μ-1,2-peroxide, which was reported to be short-lived even at −78 °C.^36^ Instead, the observed intermediate could be one of the dicopper(II) species along the possible sequence of the hydroperoxo and oxo intermediates, leading up to the formation of **[Cu_2_L(μ-OH)]^3+^**. Dicopper(II) μ-O species are highly basic^48^ and are capable of abstracting a proton from introduced moisture, the solvent, or one of the benzylic positions of the ligand. Alternatively, C–H activation of the benzylic hydrogens by the transient Cu(II) peroxo is also a possibility. The abstraction of a proton or hydrogen from the benzylic positions of the **BPMAN** ligand is also in line with the low spectroscopic yield of 29% found for this reaction and the progressive color change of the mixture to dark orange overnight. To probe this hypothesis further, a DCM solution of **[Cu_2_L]^2+^** was treated with 1 equiv of the oxo transfer agent iodosylbenzene (PhIO) under anaerobic conditions, which resulted in the formation of a progressively darker orange reaction mixture. Analysis of the ^1^H-NMR spectrum of this mixture revealed **[Cu_2_L(μ-OH)]^3+^** as the only observable species in a spectroscopic yield of 14% (see the Supporting Information Section 7.2). Reforming **[Cu_2_L](OTf)_2_** out of **[Cu_2_L(μ-OH)](OTf)_3_** is also possible by sequentially treating a THF suspension of **[Cu_2_L(μ-OH)](OTf)_3_** with 2 equiv of Co(Cp*)_2_ and an equivalent of pyridinium triflate (see Figure 5 and the Supporting Information Section 7.3).

**Figure 6:**
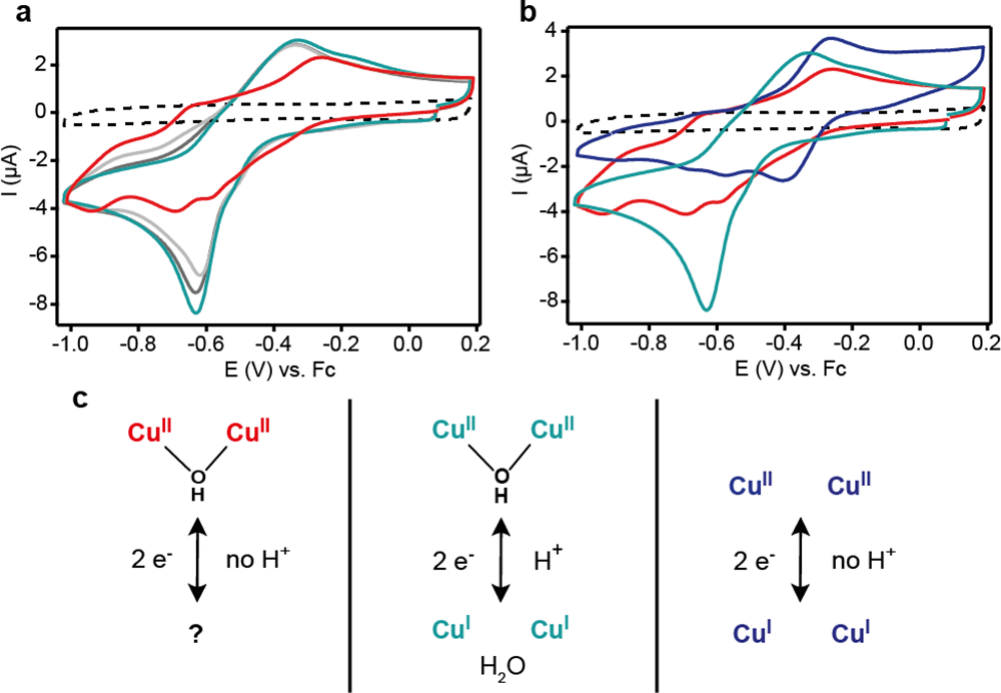
(a) CVs of 0.3 mM **[Cu_2_L(μ-OH)]^3+^** with no (red), 0.15 mM (light gray), 0.6 mM (dark gray), and 1.0 mM (light blue) TEAPF_6_ present. (b) CVs of 0.3 mM **[Cu_2_L(μ-OH)]^3+^** with (light blue) and without (red) 1.0 mM TEAPF_6_ compared to the 0.3 mM **[Cu_2_L]^2+^** redox couple (dark blue). (c) Schematic overview of the redox processes taking place in figure (b). Conditions: 0.1 M TBAPF_6_ in MeCN, Ar atmosphere, 293 K, 100 mV/s scan rate.

Next, the aptitude of the reduced **[Cu_2_L](OTf)_2_** complex to form the Cu^II^-OH-Cu^II^ motif was confirmed in electrochemical experiments as well. The redox couple of **[Cu_2_L]^2+^**, lacking the μ-hydroxyl ligand, was recorded in acetonitrile solution under inert conditions showing that the oxidized **[Cu_2_L]^4+^** complex can be formed in acetonitrile (Figure 6b). From these measurements, it is evident that the reduction and oxidation peak of **[Cu_2_L]^2+^** shifted to more positive potentials compared to the Cu^II^-OH-Cu^II^ species as the *E*_1/2_ of **[Cu_2_L]^2+^** is −0.33 V vs Fc and the *E*_1/2_ of **[Cu_2_L(μ-OH)]^3+^** is −0.48 V vs Fc. In addition, the redox couple of **[Cu_2_L]^2+^** is more reversible than that of **[Cu_2_L(μ-OH)]^3+^**, which is an indication that in the absence of the bridging hydroxyl ligand, the electron transfer and reorganization between the Cu^I^-Cu^I^ and Cu^II^-Cu^II^ species becomes easier. Moreover, these observations suggest that the negatively charged μ-hydroxyl ligand stabilizes the oxidized dinuclear copper core in **[Cu_2_L(μ-OH)]^3+^** and that there is a thermodynamic driving force to form this ligand (Figure 6b,c).

In the same experiment in water, it is possible for OH^–^ to bind to the dicopper core when **[Cu_2_L]^2+^** is oxidized or exposed to oxygen, and **[Cu_2_L(μ-OH)]^3+^** instead of **[Cu_2_L]^4+^** forms (see Figure S28). Interestingly, exposure of the **[Cu_2_L]^2+^** acetonitrile solution to oxygen from the air will form the **[Cu_2_L(μ-OH)]^3+^** species only when a proton source is added, while the complex seems to undergo decomposition to unidentified species when no source of protons is available (see the Supporting Information Section 8). It is to be expected that this decomposition is caused by the abstraction of a proton from the ligand, which agrees with the observed reactivity described in the previous section. In this way, the reactivity studies and electrochemical experiments show good agreement and together highlight the fast formation of the μ-hydroxyl ligand if the complex is in the presence of both an oxygen and a proton source.

### Reaction Kinetics of the ORR and HPRR

**Figure 7:**
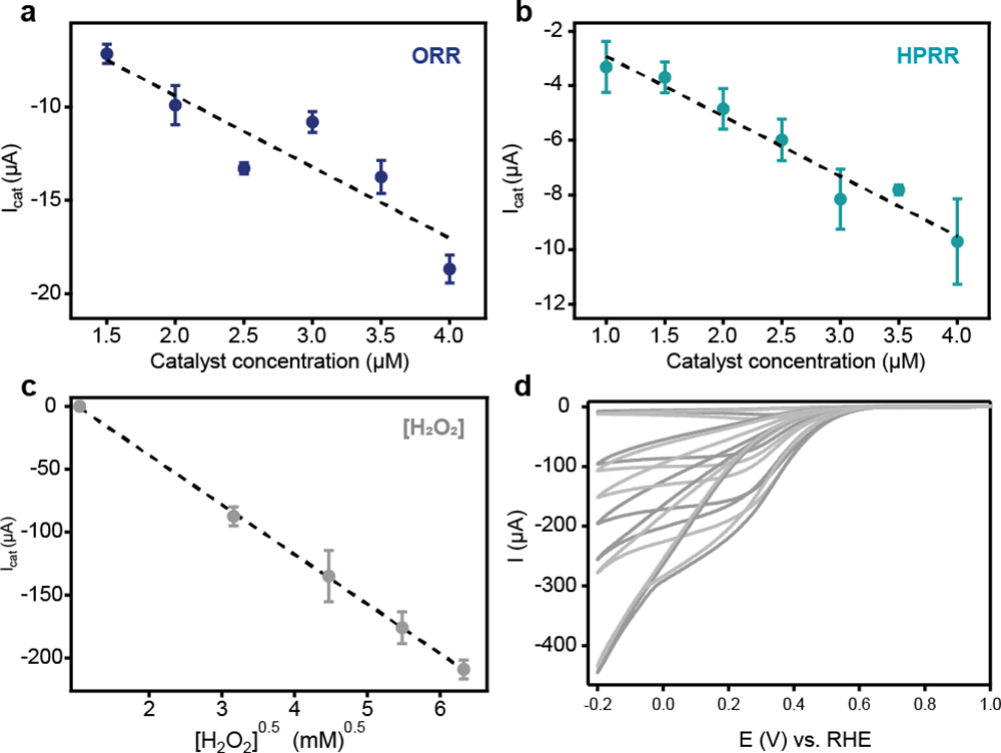
Background-corrected catalytic currents plotted against the (a) catalyst concentration for the ORR, (b) catalyst concentration for the HPRR (1.1 mM H_2_O_2_), (c) square root of the H_2_O_2_ concentration for the HPRR at 0.2 V vs RHE (0.15 mM **[Cu_2_L(μ-OH)]^3+^**), and (d) the CVs of the HPRR corresponding to figure (c). In figure (a)–(c), the data points are the average of two measurements. Conditions: 0.1 M PB pH 7, Ar or O_2_ atmosphere, 293 K, 100 mV/s scan rate.

To get insights into the catalytic mechanism that follows upon reduction of **[Cu_2_L(μ-OH)]^3+^**, catalyst concentration dependencies were determined for the ORR and HPRR (Figure 7a,b). These dependencies were studied in the regime of low catalyst concentrations (1–4 μM), where the reaction rates are not limited by the substrate. For the HPRR, this leads to CV measurements that resemble a plateau current, which is an indication that the reaction is not controlled by diffusion of substrate. In the case of the ORR, we were not able to observe plateau-like currents, although we do see a dependence of the catalytic current on the catalyst concentration. Hence, we assume that our measurements are not fully dominated by substrate consumption. Additionally, the H_2_O_2_ concentration was varied to determine the reaction dependency in the substrate (Figure 7c,d). Since a clear catalytic plateau was absent, the dependence on H_2_O_2_ concentration was determined at a potential of 0.2 V vs RHE. In both the ORR and HPRR, a linear first-order dependence of the catalytic current on the catalyst concentration was observed. In addition, a first-order dependence of the catalytic current on the substrate concentration for the HPRR was observed. For the ORR, the substrate dependence was not determined, although we assume that in the same manner, one molecule of oxygen at a time will react at the dinuclear copper site.

Next, the same experiments were used to gain insights into the reaction kinetics. For low concentrations of **[Cu_2_L(μ-OH)]^3+^**, the reaction is not limited by substrate availability and a quantitative value for the observed rate constant (*k*_obs_) can be determined from the catalytic current enhancement method using eq 1(49,50)
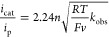
1

This equation holds for electrochemical processes that are controlled by a rate-limiting chemical step and wherein no intermolecular electron transfer steps take place. In eq 1, *i*_cat_ is the measured catalytic current, *i*_p_ is the peak current in the absence of substrate, *n* is the number of electrons transferred in the catalytic reaction, *T* is the temperature (*T* = 293 K), *v* is the scan rate (*v* = 100 mV/s), and *k*_obs_ is the observed first-order rate constant. The theoretical *i*_p_ was calculated from the Randles–Sevcik equation because of the low catalyst concentrations (see the Supporting Information Section 5). It is not possible to determine the number of electrons transferred in the ORR from stationary CV experiments; hence, we determined the *k*_obs_ for the ORR as the range between the minimum number of electrons transferred to reduce oxygen to H_2_O_2_ (*n* = 2) and the complete reduction of oxygen to water (*n* = 4). The calculated values for *k*_obs_ from the current enhancement method lie between 3.7 ± 1.0 × 10^3^ s^–1^ and 14.7 ± 4.2 × 10^3^ s^–1^ for the ORR and is 4.8 ± 1.4 × 10^3^ s^–1^ for the HPRR (see the Supporting Information Section 9). It should be noted that the *k*_obs_ values for the ORR were determined under conditions that might not be under kinetic control. For that reason, care should be taken not to overinterpret the results and a comparison between the found *k*_obs_ values for the ORR and HPRR may be invalid, given that the *k*_obs_ for ORR is probably underestimated.

Interestingly, determination of the *k*_obs_ for **[Cu_2_L(μ-OH)]^3+^** in the same manner in acetate buffer of pH 4.8 resulted in a *k*_obs_ for the HPRR that is comparable with the value at pH 7, whereas the *k*_obs_ for the ORR became almost four times as large (see Table S2). This observation indicates that the *k*_obs_ of the ORR is proton-dependent. A logical explanation for the larger *k*_obs_ of the ORR under acidic conditions is that the RDS involves a protonation step, which suggests that the protonation involved in the reduction of **[Cu_2_L(μ-OH)]^3+^** is the RDS of the ORR. The fact that the *k*_obs_ of the HPRR is not influenced by the pH can be explained by the fact that after reduction of the catalyst, the HPRR mechanism is followed by another slow step that does not lead to a general increase in the observed rate.

### Further Insights into the HPRR Mechanism

Intrigued by the proton-independent *k*_obs_ of the HPRR, we further investigated the reaction mechanism. First, a solvent kinetic isotope effect (KIE) of 1.08 ± 0.25 for the HPRR was determined under non-substrate limiting conditions (see the Supporting Information Section 10). This value indicates either the absence of a KIE, or the presence of a minor secondary KIE, suggesting that the RDS of the reaction indeed does not involve breaking of an O–H bond. Such a small KIE is in contrast with the previously determined KIE of Cu(tmpa) for the HPRR of 1.4–1.7, which was proposed to go through a Fenton-type mechanism, producing radical species upon homolytical cleavage of the O–O bond.^51^ To exclude such a Fenton-type mechanism for **[Cu_2_L(μ-OH)]^3+^**, hydroxyl radical trapping experiments for the HPRR were carried out during which no radicals could be detected (see the Supporting Information Section 11).

Interestingly, exposing a degassed D_2_O suspension of **[Cu_2_L]^2+^** to an equivalent of H_2_O_2_ led to the instant formation of a green, water-soluble species that gave rise to a different paramagnetic ^1^H-NMR spectrum than was found for **[Cu_2_L(μ-OH)]^3+^** (see the Supporting Information Section 7.4). Additionally, two absorption bands arose in the UV–Vis spectrum at 620 and 660 nm after the reaction of **[Cu_2_L]^2+^** with H_2_O_2_ (see Figure S24). These two observations suggest that oxidation of the Cu centers to Cu(II) has occurred. The difference in NMR spectra raised the question as to what might be bound to the copper centers. The oxidation of the Cu centers pointed toward cleavage of the O–O bond of H_2_O_2_ and hinted at the fact that the species being formed might be the **[Cu_2_L(μ-OH)_2_]^2+^** species that we postulated to form at high pH regimes in the *E*–pH diagram. Computational studies (see the Supporting Information Section 14) indeed show that formation of **[Cu_2_L(μ-OH)_2_]^2+^** from **[Cu_2_L]^2+^** and H_2_O_2_ is energetically favorable by approximately 38 kcal/mol. To investigate the feasibility of a bis-hydroxide complex, we exposed a **[Cu_2_L(μ-OH)]^3+^** solution in D_2_O to an equivalent of KOH (see the Supporting Information Section 7.5). This indeed gave rise to the same resonances in the paramagnetic ^1^H-NMR spectrum as those found for the reaction between **[Cu_2_L]^2+^** and H_2_O_2_. Considering the reactants in the two separate reactions and the measurements on the *E*–pH diagram that indicate that under basic conditions such a species can form, led us to confirm this new species as **[Cu_2_L(μ-OH)_2_]^2+^**. The limited stability of this bis-hydroxide upon removal of the aqueous solvent however prohibited further characterization of this species. The instability of the **[Cu_2_L(μ-OH)_2_]^2+^** species suggests that under nonbasic conditions, one of the μ-OH ligands is readily protonated to produce **[Cu_2_L(μ-OH)]^3+^** and H_2_O.

Combining the experiments on the HPRR, strong evidence is found for metal–metal cooperativity being involved in the catalytic mechanism. First, there is a first-order dependence of the catalytic rate on the concentration of catalyst and the concentration of H_2_O_2_. This validates that a single molecule of H_2_O_2_ will be reduced at the dicopper core of the catalyst. Another observation is the fact that **[Cu_2_L(μ-OH)_2_]^2+^** is formed when **[Cu_2_L]^2+^** is reacted with H_2_O_2_. Such a species can only form if the O–O bond is cleaved after binding of H_2_O_2_ between both copper cores. In addition, **[Cu_2_L(μ-OH)]**^**3**+^ catalyzes the HPRR via a different pathway than the mononuclear Cu(tmpa) complex.^51^ Although for Cu(tmpa), a significant KIE is observed and the mechanism is proposed to go through a Fenton-type mechanism, our experiments on **[Cu_2_L(μ-OH)]^3+^** exclude such a pathway. It is therefore most likely that **[Cu_2_L(μ-OH)]^3+^** binds H_2_O_2_ via a different binding motif involving interactions with both copper centers, instead of end-on binding as was proposed for Cu(tmpa).^51^

### Selectivity of the ORR

After gaining insights into the HPRR mechanism and having a strong indication that both copper centers cooperate in the binding of H_2_O_2_ and cleavage of the O–O bond, we set out to investigate the mechanism and cooperativity in the ORR. As stated before, H_2_O_2_ is found as a detectable intermediate in the electrochemical ORR mechanism of other copper complexes with tetradentate pyridylamine ligands.^30,33,35,40,41^ Hence, it is interesting to see whether the HPRR is part of the ORR occurring via two sequential two-electron reductions or whether **[Cu_2_L(μ-OH)]^3+^** can directly reduce oxygen to water via a four-electron pathway, similar to MCOs. Therefore, rotating (ring) disk electrode (R(R)DE) experiments were employed.

First, it is important to realize that in the same manner as in stationary CV experiments, the homogeneous catalytic activity of **[Cu_2_L(μ-OH)]^3+^** in RRDE experiments was ensured by investigating the formation of any active deposits (see the Supporting Information Section 12.3). Upon recording a CV in a solution of **[Cu_2_L(μ-OH)]^3+^** and subsequently recording a CV of the same electrode in a blank solution that does not contain any catalyst, it was shown that a catalytically active, heterogeneous deposit forms on the electrode during the ORR. Under RRDE conditions, this deposit is less active than the homogeneous catalyst in solution (see Figure S39a). Next, the selectivity for the four- or two-electron ORR of the catalyst in solution and the formed deposits were compared, and it was shown that these experiments have a comparable outcome (see Figure S39b). This verifies that the formed deposits do not affect the observed selectivity of **[Cu_2_L(μ-OH)]^3+^**. To ensure that we exclusively study the homogeneous catalyst in solution, only the first scan of any RRDE experiment is used to assign the catalytic activity and selectivity of **[Cu_2_L(μ-OH)]^3+^**. In chronoamperometry measurements (CA), it is not possible to exclude contribution of any deposited species that form during the experiment. However, we do not observe any change in selectivity or activity over the course of CA experiments, indicating that no buildup of a catalytically competent species occurs. An exception is when a potential close to the onset of 0.4 V vs RHE is applied. In this case, we observe a change in the selectivity over time, which we attribute to the fact that under these conditions the current is low in general, which will easily lead to deviations in the selectivity (see Figure S38).

**Figure 8:**
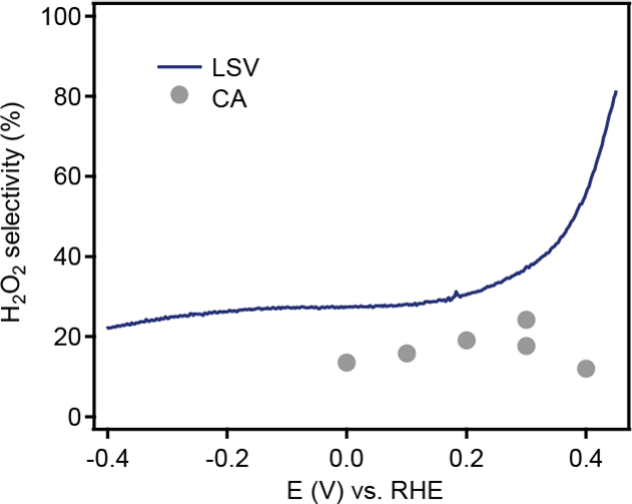
H_2_O_2_ selectivity of **[Cu_2_L(μ-OH)]^3+^** in the ORR as determined from RRDE LSV measurements (dark blue line) and CA measurements (gray dots) at different potentials. The LSV currents were corrected for the currents measured between 0.8 and 1.0 V vs RHE and the CA points were corrected for the currents measured at 0.8 V vs RHE preceding the experiment. Conditions: 0.15 mM **[Cu_2_L(μ-OH)]^3+^**, O_2_ atmosphere, 0.1 M PB pH 7, 293 K, 1600 RPM, Pt ring at 1.2 V vs RHE.

The RDE CVs of **[Cu_2_L(μ-OH)]^3+^** recorded in PB of pH 7 at various rotation rates show slow catalysis of the ORR because even at a strongly negative potential of −0.4 V vs RHE, no clear mass-transport limited plateau is reached (see Figure S35). More interestingly, RRDE measurements of **[Cu_2_L(μ-OH)]^3+^** were carried out to determine the selectivity of the ORR over the full potential window. In RRDE measurements, the hydrogen peroxide that is produced at the GC disk can be quantified by oxidation at the Pt ring (see the Supporting Information Section 12.2). Figure 8 shows that **[Cu_2_L(μ-OH)]^3+^** selectively catalyzes the four-electron ORR in both linear sweep voltammetry (LSV) and CA measurements, producing more than 70% H_2_O over the main part of the potential window. Only close to the onset potential in the LSV, the selectivity to H_2_O_2_ is high, which is likely to be less accurate as the currents are (too) low. To exclude that any H_2_O_2_ is decomposed by the catalyst before it can be detected at the ring, the stability of H_2_O_2_ solutions in the presence of **[Cu_2_L(μ-OH)]^3+^** was studied. These experiments confirm that the catalyst is not capable of breaking down H_2_O_2_ before it reaches the ring (see the Supporting Information Section 13).

It is likely that the preference of **[Cu_2_L(μ-OH)]^3+^** to catalyze the ORR in a four-electron process arises from metal–metal cooperativity in the dinuclear copper core. In **[Cu_2_L(μ-OH)]^3+^**, the naphthyridine-based ligand enforces the two copper atoms to bind in close proximity and in this way facilitates bridging binding modes for exogeneous ligands, like the peroxo intermediate.^36^ This is in contrast with mononuclear copper complexes that can either bind a (hydro)peroxo ligand in an end-on manner or upon reaction with oxygen will slowly dimerize to form a peroxo dicopper complex,^21^ as is observed for Cu(tmpa).^52^ The ORR selectivity to water of 70% by **[Cu_2_L(μ-OH)]^3+^** stands out compared to the selectivity of Cu(tmpa), which only converts 20% of oxygen to water in the presence of high concentrations of oxygen.^35^ The improved selectivity of **[Cu_2_L(μ-OH)]^3+^** points to a different mechanism in which the enforced bridging geometries result in the thermodynamic stabilization of the formed (hydro)peroxo intermediates and facilitate the easy cleavage of the O–O bond. Furthermore, we hypothesize that the energy barrier to release any intermediate ORR products like H_2_O_2_ from the dinuclear copper core is high. On the one hand, the release of H_2_O_2_ is disfavored kinetically due to the proximal oxygen being not easily accessible for protonation. On the other hand, liberating any of the (hydro)(per)oxo ligands results in the formation of the bare dinuclear Cu^II^ Cu^II^ core, which is energetically disfavored due to severe electrostatic repulsions of the two copper centers confined in the rigid naphthyridine framework. Combined, we believe that these are the main arguments that steer the reaction to the four-electron pathway and explain the significant difference in selectivity compared to mononuclear analogues.

### Discussion of the Catalytic Mechanism of **[Cu_2_L(μ-OH)]^3+^**

**Figure 9:**
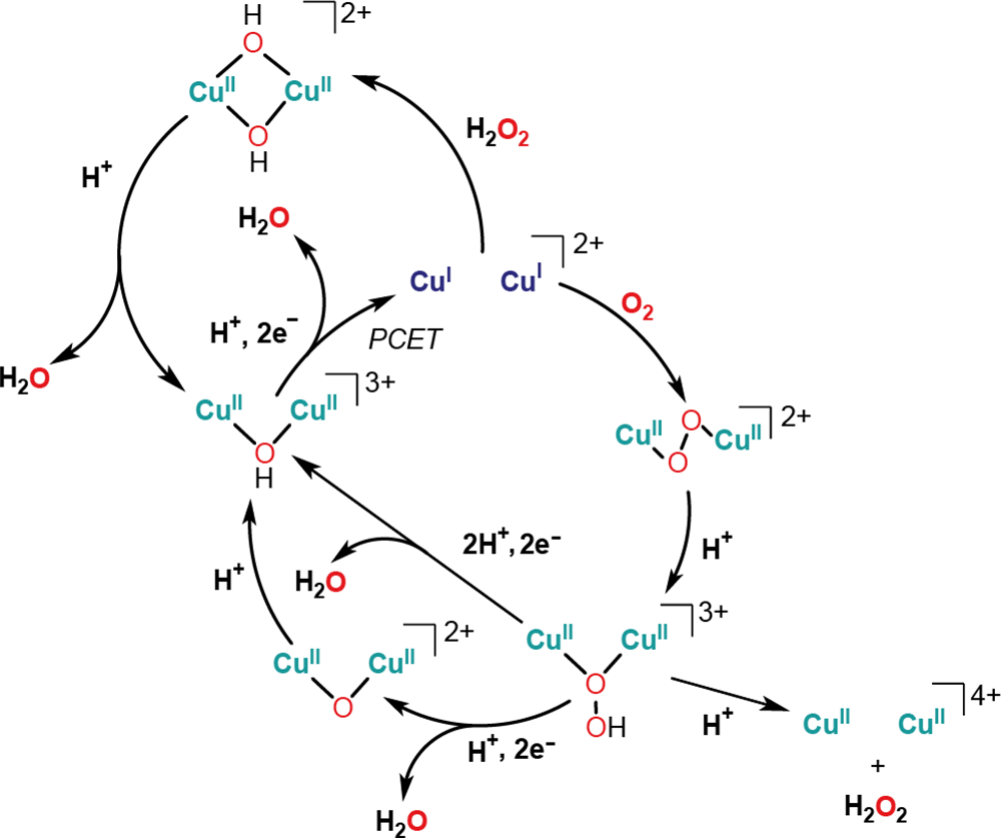
Proposed stepwise mechanism for the ORR and HPRR by **[Cu_2_L(μ-OH)]^3+^**. For clarity, the **BPMAN** ligand is not depicted. The PCET step takes place below pH 7.

Combining the investigations of the ORR and HPRR, a mechanism for these reactions catalyzed by **[Cu_2_L(μ-OH)]^3+^** could be established (see Figure 9). Starting the catalytic cycle, **[Cu_2_L(μ-OH)]^3+^** needs to be reduced and the bridging ligand must dissociate from the dicopper core. Measurements have shown that below pH 7, two electrons and one proton are transferred and the hydroxide is released as H_2_O. Under more alkaline conditions, a **[Cu_2_L(μ-OH)_2_]^2+^** species forms that is reduced in a two electron and one proton process. In addition, CV measurements have shown that the μ-OH moiety stabilizes the Cu^II^-OH-Cu^II^ core, which makes activation of the catalyst harder and slows down the electron transfer in general.

After reduction and release of the bridging ligand, oxygen can bind to the Cu^I^ Cu^I^ core. From a previous study by Lippard and co-workers on the same compound, it is known that the trans-μ-1,2-peroxo species can be formed;^36^ hence, it is likely that the same species will be formed here. Next, we hypothesize that a hydroperoxo species forms after protonation. RRDE experiments show that small quantities of H_2_O_2_ are produced during the ORR. It is speculated that the hydroperoxo can bind in a μ-1,1 manner as such μ-1,1-hydroperoxo species have been shown to be able to form previously.^25,53−56^ Although we were not able to isolate this intermediate species, it is anticipated that the high selectivity of **[Cu_2_L(μ-OH)]^3+^** for the four-electron ORR arises from the relatively strong binding of the (hydro)peroxo moiety between both copper atoms. This results in the four-electron ORR taking place via protonation of the distal oxygen atom and prevents formation of the disfavored bare Cu^II^ Cu^II^ species. In the next step, two electrons and two protons are transferred, resulting in cleavage of the O–O bond and generation of H_2_O. Whether this step proceeds directly or via the intermediate production of a μ-oxo species cannot be addressed experimentally. We have shown however through our studies with the oxo-transfer agent PhIO that if an oxo-species would form, it would readily be protonated, reforming **[Cu_2_L(μ-OH)]^3+^**.

Next to the ORR mechanism, the off-cycle HPRR mechanism was studied. After reduction of **[Cu_2_L(μ-OH)]^3+^**, H_2_O_2_ binds to the dicopper core, upon which two electrons are transferred to break the O–O bond and the **[Cu_2_L(μ-OH)_2_]^2+^** species is formed. Due to the relative instability of this bis-μ-OH species, we hypothesize that this species will be quickly protonated to regenerate the stable **[Cu_2_L(μ-OH)]^3+^** complex. Though this HPRR mechanism is not part of the ORR, this reaction as well shows how having two copper centers in close proximity allows for the cooperative activation of substrates.

Altogether, the catalytic pathways of **[Cu_2_L(μ-OH)]^3+^** provide multiple evidence for a cooperative catalytic mechanism, which contrasts the mechanisms of previously reported dicopper-based catalysts for the electrocatalytic ORR. First of all, our studies on the reduction of **[Cu_2_L(μ-OH)]^3+^** and oxidation of **[Cu_2_L]^2+^** show that a bridging ligand will always form between both copper centers. This is established by the 1,8-naphthyridine backbone that forces both copper atoms together and avoids catalysis at a single copper site. A first-order dependence in the substrate and catalyst for the HPRR and ORR further support this reactivity at a dicopper site. Moreover, the bridged binding of reaction intermediates previously reported by Lippard *et al*.^36^ and observed herein for the **[Cu_2_L(μ-OH)_2_]^2+^** species underlie cooperativity between both copper sites during catalysis. In addition, the mechanism of the ORR and HPRR found for **[Cu_2_L(μ-OH)]^3+^** differ from the ones found for Cu(tmpa), resulting in improved selectivity for the four-electron ORR of more than 70% as a result of metal–metal cooperativity.

Subsequently, we can link the activity and selectivity of this homogeneous catalyst to the ORR mechanism found in MCOs. In both the synthesized **[Cu_2_L(μ-OH)]^3+^** complex and T3 site of the MCO resting state, a μ-OH ligand is present.^12^ From our study, it is apparent that the formation of the Cu-OH-Cu motif is a severe thermodynamic driving force; hence, the presence of the bridging hydroxyl ligand can be linked to slow electron transfer kinetics and a large energy barrier to activate the catalyst. This is comparable to what is observed for MCOs, where the presence of the μ-OH makes reduction of the RO state too slow to be part of the catalytic cycle.^20^

In the enzymatic pathway, an oxygen molecule can bind to the FR state of MCOs resulting in the formation of a μ_3_–1,1,2-peroxo intermediate in an irreversible manner.^57^ The binding mode of the μ_3_–1,1,2 peroxide is essential for the cleavage of the O–O bond and the four-electron reduction of oxygen.^57^ When **[Cu_2_L]^2+^** reacts with oxygen, the peroxo intermediate binds differently, likely as a μ-1,2-peroxide, which was observed previously.^36^ However, we can explain the greatly improved selectivity toward the overall four-electron ORR by the ability to bind catalytic intermediates in a bridging manner, which is not possible for the mononuclear analogue Cu(tmpa).

To complete the catalytic ORR cycle in MCOs, the second two-electron reduction and cleavage of the O–O bond result in formation of the NI state containing a μ_3_-O and μ-OH^–^.^17^ Next, the FR state is regenerated rapidly by protonation and reduction of this highly basic μ_3_-O ligand.^19^ In **[Cu_2_L(μ-OH)]^3+^**, a μ_3_-oxo cannot form, and instead, we hypothesize that **[Cu_2_L(μ-OH)]^3+^** is regenerated upon the two electron reduction of the hydroperoxo intermediate, which is more reminiscent of the decay of NI to RO.

## Conclusions

To conclude, by combining electrochemical measurements and reactivity studies, insights into the mechanisms of the ORR and HPRR catalyzed by **[Cu_2_L(μ-OH)]^3+^** have been obtained. Some of the key active species along these catalytic pathways were identified, resulting in a detailed understanding of the activity and selectivity of this catalyst. From our study, it is evident that metal–metal cooperativity during both HPRR and ORR could be established by the rigid dinucleating 1,8-naphthyridine-based ligand that keeps both copper atoms in **[Cu_2_L(μ-OH)]^3+^** closely together during catalysis. Until now, no other studies on biomimetic copper complexes have given such direct evidence for cooperativity during electrocatalysis of the ORR or HPRR. Following from this, the dinuclear **[Cu_2_L(μ-OH)]^3+^** complex catalyzes the HPPR and ORR in a different manner than its mononuclear analogue Cu(tmpa).^35,51^ Due to the presence of the Cu-OH-Cu motif in **[Cu_2_L(μ-OH)]^3+^**, the dicopper core is stabilized and its reduction follows a PCET pathway. Moreover, the (hydro)peroxo intermediates that can form during the HPRR and ORR are bound between both copper centers, and as a result, the formation of hydrogen peroxide is disfavored and the selectivity for the four-electron reduction of oxygen is greatly improved. Taken together, this work helps explain how the electrocatalytic activity and selectivity of the ORR are affected by a rigid dinuclear copper site, which ultimately can be used for the development of oxygen reduction catalysts based on earth-abundant metals.

## Supporting Information Available

The ESI contains experiments, synthesis, hydrogen peroxide reduction by **[Cu_2_L(μ-OH)]^3+^**, catalyst stability, scan rate dependence and diffusion coefficient calculations, pH dependency studies, reactivity studies, *k*_obs_, HPRR measurements, radical trapping experiments, RRDE measurements, stability of H_2_O_2_ solutions, computational methods, NMR spectra, electrochemical experiments, UV–vis spectra, and EPR spectra. (PDF)
