# Supplementary material for: Mechanistic Investigations into the Selective Reduction of Oxygen by a Multicopper Oxidase T3 Site-Inspired Dicopper Complex
Source: ACS Catal. 2023 Apr 12;13(8):5712–22. doi: 10.1021/acscatal.3c01143 (PMC10127274; doi:10.1021/acscatal.3c01143)
Supplement: Supplementary file 1 — cs3c01143_si_001.pdf [file cs3c01143_si_001.pdf]

Mechanistic Investigations into the Selective Reduction of Oxygen by a Multicopper Oxidase T3 Site-Inspired Dicopper Complex

Supporting Information

Phebe H. van Langevelde[a‡], Errikos Kounalis[b‡], Lars Killian[a,b‡], Emily C. Monkcom[b], Daniël L. J. Broere[b*], and Dennis G. H. Hetterscheid[a*]

[a]Leiden Institute of Chemistry, Leiden University, 2300 RA, Leiden, The Netherlands

[b]Organic Chemistry and Catalysis, Institute for Sustainable and Circular Chemistry, Faculty of Science, Utrecht University, Universiteitsweg 99, 3584 CG, Utrecht, The Netherlands

[‡]These authors contributed equally

*Corresponding authors: Daniël L. J. Broere (email: d.l.j.broere@uu.nl), and Dennis G. H. Hetterscheid (email: d.g.h.hetterscheid@chem.leidenuniv.nl).

1. EXPERIMENTAL 3

1.1 GENERAL 3
1.2 ELECTROCHEMICAL EXPERIMENTS 4

2. SYNTHESIS 6

2.1 SYNTHESIS OF 2,7-BIS(CHLOROMETHYL)-1,8-NAPHTHYRIDINE 6
2.2 SYNTHESIS OF 2,7-BIS[BIS(2-PYRIDYLMETHYL)AMINOMETHYL]-1,8-NAPHTHYRIDINE (BPMAN) 8
2.3 SYNTHESIS OF [BPMANCU$_2$(M-OH)](OTF)$_3$ 10
2.4 SYNTHESIS OF [BPMANCU$_2$](OTF)$_2$ 15

3. HYDROGEN PEROXIDE REDUCTION BY [CU$_2$L(M-OH)]$^{3+}$ 17

4. CATALYST STABILITY DURING CV MEASUREMENTS 17

4.1 DEPOSIT TESTS IN CV 18
4.2 ELECTROCHEMICAL QUARTZ CRYSTAL MICROBALANCE MEASUREMENTS 23

5. SCAN RATE DEPENDENCE AND DIFFUSION COEFFICIENT CALCULATIONS 24

6. PH DEPENDENCE STUDIES 26

6.1 CV MEASUREMENTS 27
6.2 DPV MEASUREMENTS 27
6.3 UV-VIS AT VARYING PH 28

7. REACTIVITY STUDIES 29

7.1 AIR EXPOSURE EXPERIMENT WITH [CU$_2$L]$^{2+}$ 29
..... 30
7.2 EXPERIMENT WITH [CU$_2$L]$^{2+}$AND PHIO 30
7.4 EXPERIMENT WITH [CU$_2$L]$^{2+}$AND H_2O_2 33
7.5 TREATMENT OF [BPMANCU$_2$(M-OH)](OTF)$_3$ WITH KOH 35

8 ELECTROCHEMICAL MEASUREMENTS OF [CU$_2$L]$^{2+}$ 39

9. K$_{OBS}$ FOR ORR AND HPRR IN DIFFERENT BUFFERS 41

10. HPRR MEASUREMENTS IN D_2O 43

11. RADICAL TRAPPING EXPERIMENTS 44

12. RRDE MEASUREMENTS 45

12.1 RDE CVS AND KOUTECKY-LEVICH ANALYSIS 45
12.2 H_2O_2 SELECTIVITY IN RRDE 45
12.3 FORMATION OF DEPOSITS IN RRDE EXPERIMENTS 48

13. STABILITY OF H_2O_2 SOLUTIONS IN THE PRESENCE OF [CU$_2$L(M-OH)]$^{3+}$ 49

14. COMPUTATIONAL METHODS 50

14.1 GENERAL REMARKS 50
14.2 OPTIMIZED XYZ COORDINATES AND COMPUTED ENERGIES 51

REFERENCES 61

1. Experimental

1.1 General

All manipulations were performed under inert atmosphere using standard Schlenk techniques or inside of a N_2-filled MBRAUN LABmaster DP glovebox using anhydrous solvents and reagents, unless noted otherwise. Glassware was dried at 130 ˚C prior to use. Solvents were collected from an MBRAUN MB-SPS-800 solvent purification system and stored over 4 Å molecular sieves, except for CH_2Cl_2, which was stored over 3 Å molecular sieves. Acetonitrile was stored over 3 Å molecular sieves before being passed over a pad of activated alumina. Deuterated solvents were obtained from Cambridge Isotope Laboratories, degassed, and stored over 3 Å molecular sieves when necessary. D_2O for the kinetic isotope experiments was obtained from Eurisotop (99.9 % D). All commercial reagents were used as received and were obtained from Sigma Aldrich, Strem or Acros. All electrolyte solutions were prepared using high purity salts, NaH_2PO_4 (Merck, Suprapur ≥99.99 %), Na_2HPO_4 (Honeywell Fluka, TraceSELECT ≥99.999 %), Na_2SO_4 (Alfa Aesar, Puratronic 99.9955 %), $NaClO_4{\cdot}H_2O$ (Merck, EMSURE ≥99.7 %), H_3BO_3 (Sigma-Aldrich, 99.999 % trace metals basis), CH_3COOH (Honeywell Fluka, Trace SELECT ≥99.0 %), H_3PO_4 (85 wt. % in H_2O, Sigma-Aldrich, 99.999% trace metals basis) and Mili-Q water. H_2O_2 solution was obtained as a 30 wt% solution in water (Merck) and diluted to the desired concentration. The H_2, O_2, and Ar gasses used in electrochemical experiments were supplied by Linde.

NMR data was recorded on an Agilent MRF 400 equipped with a OneNMR probe and Optima Tune system or a Varian VNMR-S-400 equipped with an AutoX probe. All resonances in ^{1}H-NMR were referenced to residual proteo solvent peaks (7.26 for $CDCl_3$, 7.16 for C_6D_6, 5.32 for CD_2Cl_2 and 4.79 for D_2O). The respective ^{19}F NMR spectra were referenced employing absolute referencing using the ^{1}H NMR spectrum of the same sample. EPR spectra were recorded on a Bruker EMXPlus X-band spectrometer. UV-Vis data was recorded on a PerkinElmer Lambda950 spectrophotometer or an Agilent Varian Cary 50 spectrophotometer. The pH of the electrolyte solutions was altered by addition of diluted solutions of $NaOH{\cdot}H_2O$ (Merck, Suprapur 99.9%) or H_2SO_4 (96%, Merck, Suprapur). The pH of all electrolyte solutions was determined on a HI 4222 pH meter (Hanna Instruments). The concentration of H_2O_2 in aqueous solution was determined with H_2O_2 test strips by use of photometric determination with the Merck Reflectoquant system.

1.2 Electrochemical experiments

All electrochemical measurements were recorded using Autolab PGSTAT 12, 204, or 128N potentiostats and Autolab NOVA 2 software. In all stationary CV experiments, except EQCM, the working electrode was a PEEK encapsulated glassy carbon (GC) disk (A = 0.071 cm^2, Metrohm) submerged in the electrolyte solution. This GC electrode was polished prior to every experiment on a Struers LaboPol-30 polishing machine with diamond polish (1.0 µm, 30 seconds) and silica suspension (0.04 µm, 2 minutes) on polishing cloths (Dur-type). Afterwards, the GC electrode was sonicated in Mili-Q water for 15 minutes to remove excess polishing solution. In all experiments a gold wire counter electrode was used, which was flame annealed and rinsed with Mili-Q before every experiment. For all measurements in aqueous solutions, a reversible hydrogen electrode (RHE) was used. This RHE was made from a Pt mesh and filled with electrolyte solution. H_2 was bubbled though the solution during the experiment and the RHE was connected to the main cell compartment via Luggin capillary. Before every electrochemical experiment, the electrolyte solution was bubbled with either Ar or O_2 gas for at least 15 minutes. During the experiment a flow of the gas was applied over the solution. All electrochemical experiments were carried out in custom-built glass cells that were boiled and rinsed with Mili-Q water prior to every experiment. In addition, the glassware was routinely cleaned by soaking in a solution of $KMnO_4$ (1 g/L) in 0.5 M H_2SO_4 overnight. Afterwards, the glassware was placed in a diluted solution of H_2SO_4 and H_2O_2, rinsed and boiled three times in Mili-Q. Unless stated otherwise, background corrected currents are obtained by subtracting the currents observed in the blank electrolyte solution with no catalyst present under identical conditions, measured directly before measuring the analyte solution.

For pH studies, a 0.01 M Britton-Robinson buffer was prepared by making a solution of 0.01 M H_3BO_3, 0.01 M CH_3COOH, and 0.01 M H_3PO_4. In addition, 0.05 M Na_2SO_4 was added as supporting electrolyte. The pH of the solution was changed by addition of small quantities of 1 M H_2SO_4 and 1 M NaOH solutions. Between measurements, the solution was vigorously bubbled to ensure proper mixing.

For CV experiments in acetonitrile solution, the glassware was dried in an oven at 140 °C overnight after boiling in MiliQ. The acetonitrile used in electrochemical experiments was either used as received (ACROS, 99.9%, dry) or taken from a PureSolv MD5 solvent dispenser (Demaco) and stored on molecular sieves. Tetrabutylammonium hexafluorphosphate ($TBAPF_6$, Sigma-Aldrich, ≥99.0 %) was used to make a 0.1 M electrolyte solution. Triethylammonium hexafluorphosphate ($TEAPF_6$) was synthesized from NH_4PF_6 and triethylamine. Acetic acid was obtained from Sigma-Aldrich (≥99.99 %). Instead of an RHE, Ag/AgCl (3 M

KCl) was used as a reference electrode. After the experiments, this Ag/AgCl electrode was referenced to Fc/Fc^+ by recording the ferrocene/ferrocenium redox couple directly in the electrolyte solution.

EQCM experiments were performed in a PEEK electrochemical cell. A gold-coated quartz crystal EQCM electrode (Metrohm, 0.35 cm^2) was used as WE in combination with the Metrohm EQCM potentiostat module. A gold counter electrode and RHE reference electrode were used in the same manner as described for general CV experiments. All EQCM measurements were conducted on a new EQCM electrode and a blank measurement of the electrode was recorded prior to measurements of the catalyst.

For all R(R)DE experiments a Pine Instruments MSR rotator was used in combination with an Autolab PGSTAT 12 potentiostat. R(R)DE measurements were performed in a 40 mL custom-built three-electrode electrochemical cell, with a Au counter electrode and RHE reference electrode prepared in the same manner as for stationary CV experiments. In the R(R)DE cell the gold counter electrode is separated from the main compartment by a glass frit. A Pine Instruments ChangeDisk RRDE electrode (E6R1PK) was used as working electrode. This electrode consists of a PEEK shroud with a GC disk (A = 0.196 cm^2) surrounded by a Pt ring. Prior to every experiment, the GC disk and Pt ring were polished separately on a Struers LaboPol-30 polishing machine with diamond polish (1.0 µm, 3 minutes) and silica suspension (0.04 µm, 3 minutes) on polishing cloths (Dur-type). Afterwards, the electrode was sonicated in Mili-Q water for 15 minutes. Before every R(R)DE experiment, the electrolyte solution was bubbled with either Ar or O_2 gas for at least 25 minutes. During the experiment the gas was bubbled through the solution.

2. Synthesis

2.1 Synthesis of 2,7-bis(chloromethyl)-1,8-naphthyridine

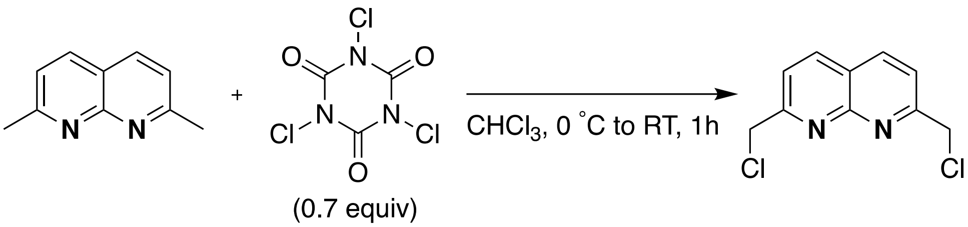

The compound was synthesized according to a modified literature procedure.[1] A suspension of trichloroisocyanuric acid (1.03 g, 4.42 mmol) in chloroform (15 mL) was added in portions to a cooled (0 °C), stirring solution of 2,7-dimethyl-1,8-naphthyridine (1.00 g, 6.32 mmol) in chloroform (15 mL), resulting in a turbid, canary yellow mixture. After stirring for 15 min, the cooling was removed, and the mixture was allowed to warm up to room temperature. After 50 min the starting material was consumed (monitored by TLC) and water was added (20 mL) causing a slurry to form. The solids were removed using a glass frit and the filtrate was washed with water (2 x 20 mL) and brine (20 mL). The organic layers were dried over Na_2SO_4, filtrated and concentrated under vacuum to a pale-yellow solid. The compound could be purified by flash column chromatography (silica, DCM:MeOH, 97:3), which after drying over Na_2SO_4, filtration and drying under vacuum resulted in the formation of the title compound as an off-white powder in 33% yield (0.48 g, 2.09 mmol). Spectroscopic data is consistent with the literature.[1]

Note: The monochlorinated compound is also formed in appreciable yields during the reaction and can be isolated during flash column chromatography and chlorinated further.

^{1}H NMR (400 MHz, $CDCl_3$, 298 K): δ = 8.25 (d, $^3J_{H,H}$ = 8.4 Hz, 2H), 7.76 (d, $^3J_{H,H}$ = 8.4 Hz, 2H), 4.88 (s, 4H) ppm.

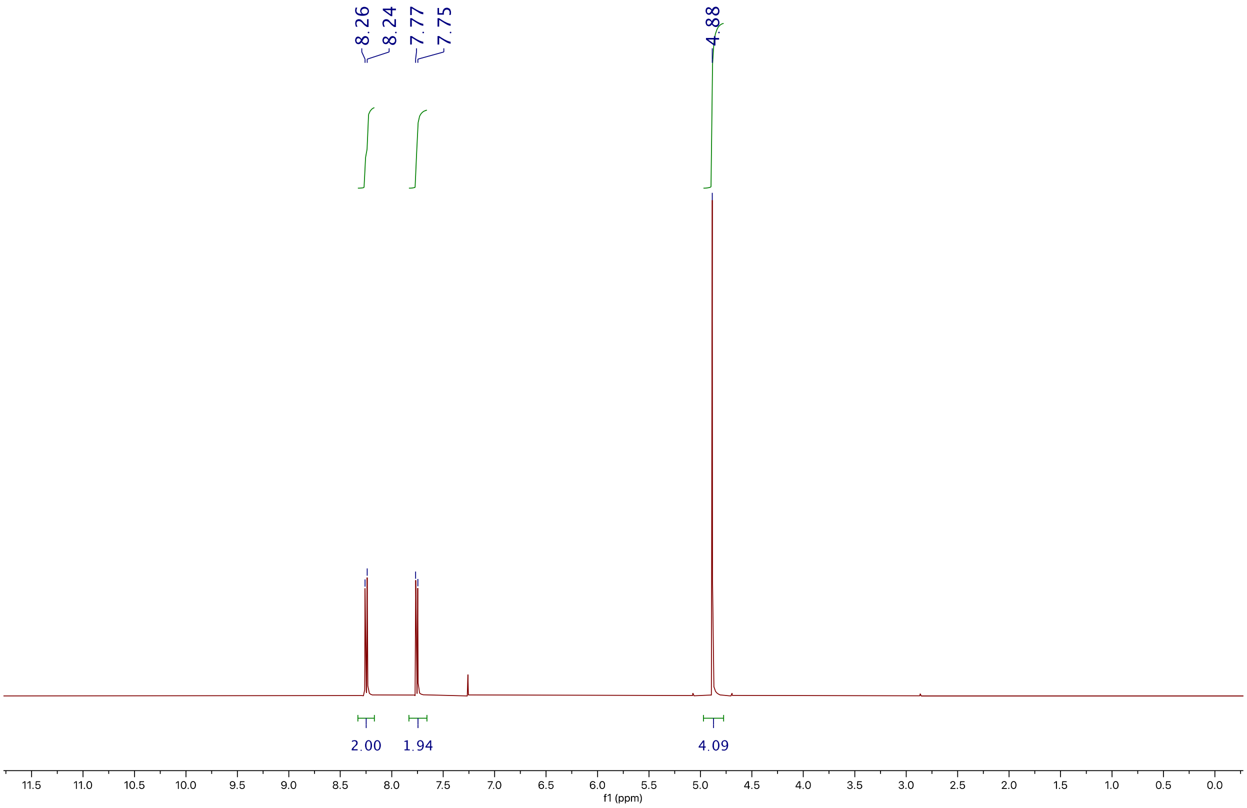

Figure S1 The ^1H NMR spectrum of 2,7-bis(chloromethyl)-1,8-naphthyridine in $CDCl_3$ at 25 °C

2.2 Synthesis of 2,7-bis[bis(2-pyridylmethyl)aminomethyl]-1,8-naphthyridine (BPMAN)

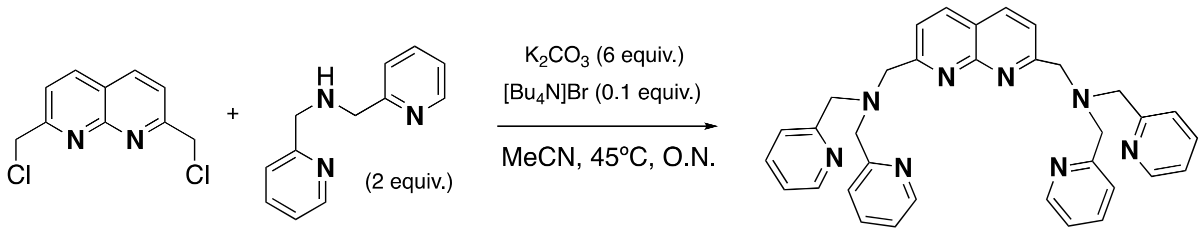

In a flame-dried 3-neck round-bottomed flask, bis(2,7-dichloromethyl)-1,8-naphthyridine (1.0 g, 4.4 mmol), K_2CO_3 (3.6 g, 26 mmol, 6 eq.) and $[Bu_4N]Br$ (145 mg, 0.45 mmol, 0.1 eq.) were suspended in 200 mL MeCN (anhydrous) under N_2 atmosphere. Next, bis(2-methylpyridyl)amine (1.61 mL, 8.9 mmol, 2 eq.) was added in one portion and the reaction mixture was heated to 45 °C and stirred overnight. During this time, the brown reaction mixture became progressively cloudier to give a pink-ish suspension. After 15 h, the reaction mixture was left to cool to room temperature and transferred to a separatory funnel using CH_2Cl_2 (3 x 150 mL). This mixture was washed sequentially with aqueous NaOH solution (10% in H_2O), and water. The organics were dried over Na_2SO_4, and all solvents were removed under a dynamic vacuum. The crude solid was washed with petroleum ether (40-60) by vigorous stirring for 15 min and was subsequently collected by Büchner filtration, affording the title compound as a pale, cream colored solid in 64% yield (1.56 g, 2.81 mmol). Spectroscopic data is consistent with the literature.[2]

^{1}H NMR (400 MHz, $CDCl_3$, 298 K): δ = 8.54 (ddd, $^3J_{H,H}$ = 4.9 Hz, $J_{H,H}$ = 1.9 Hz, $J_{H,H}$ = 0.9 Hz, 4H), 8.12 (d, $^3J_{H,H}$ = 8.4 Hz, 2H), 7.87 (d, $^3J_{H,H}$ = 8.4 Hz, 2H), 7.64 (td, $^3J_{H,H}$ = 7.6 Hz, $^4J_{H,H}$ = 1.8 Hz, 4H), 7.56 (d, $^3J_{H,H}$ = 7.8 Hz, 4H), 7.14 (ddd, $^3J_{H,H}$ = 7.4 Hz, $J_{H,H}$ = 4.9 Hz, $J_{H,H}$ = 1.3 Hz, 4H), 4.11 (s, 4H), 3.93 (s, 8H) ppm.

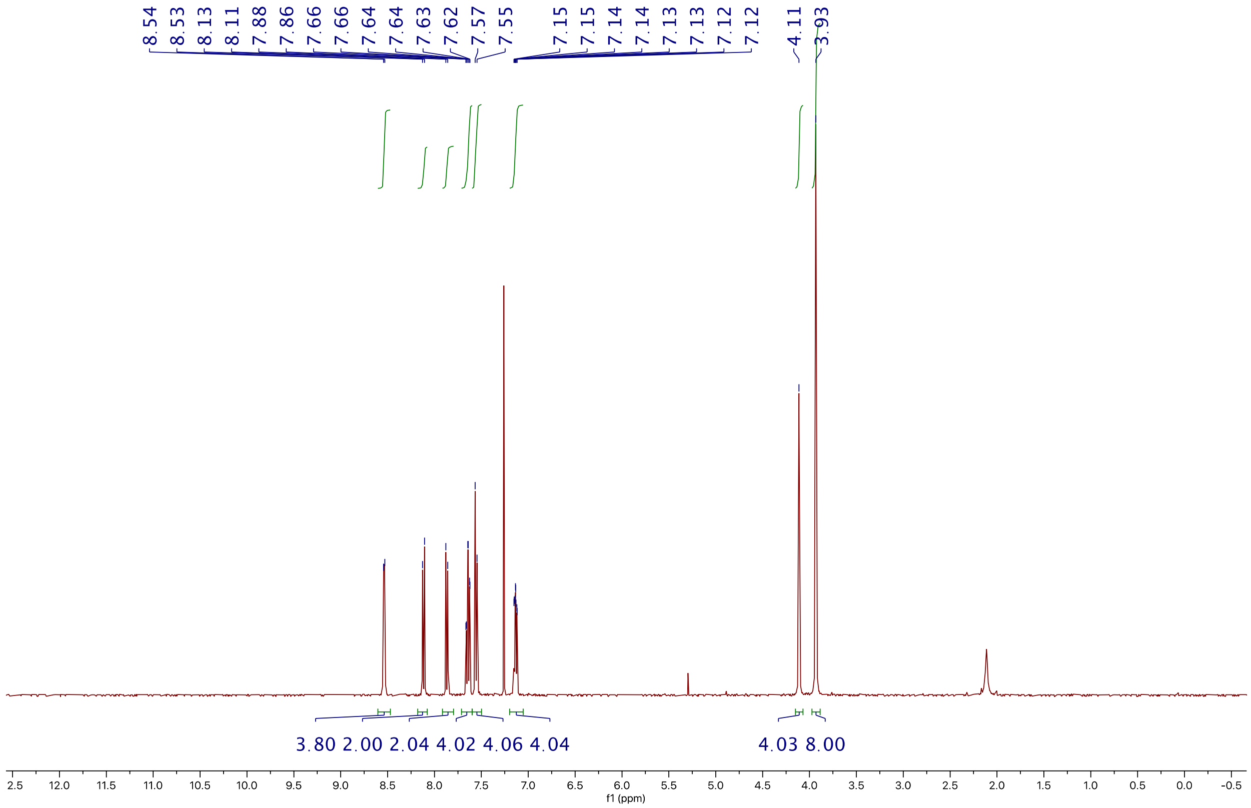

Figure S2 The ^1H NMR spectrum of BPMAN in $CDCl_3$ at 25 ℃

2.3 Synthesis of [BPMANCu₂(μ-OH)](OTf)₃

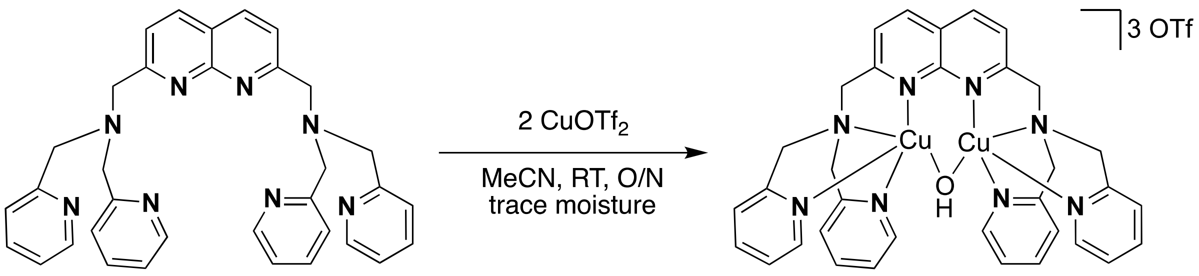

The compound was synthesized according to a modified literature procedure.[3] A suspension of BPMAN (442.1 mg, 0.8 mmol) in MeCN (75 mL) was added dropwise over the course of 30 min to a stirring, light blue solution of $Cu(OTf)_2$ (593.6 mg, 1.64 mmol) in MeCN (20 mL). The resulting dark blue solution was stirred overnight (18 h) under N_2 flow. Afterwards, the solution was concentrated to 10 mL under reduced pressure. Et_2O (100 mL) was added and the solution was placed in an ultrasonic bath for 15 minutes. The blue oil was washed with more Et_2O (4 x 50 mL), dissolved in EtOH (25 mL) and stored at -18 °C for 2 days, resulting in the formation of green crystals. The supernatant was decanted and the green crystals were washed with Et_2O (2 x 10 mL). Next, Et_2O (5 mL) was added to the supernatant which prompted the formation of a green oil within minutes. The supernatant was again decanted, Et_2O (5 mL) was added and a green oil formed again. The blue supernatant was discarded, and the green oils were combined and stripped of solvent by evaporating copious amounts of diethyl ether under reduced pressure. After drying overnight under vacuum, the title compound was obtained as a green solid in 40% yield (364.7 mg, 319 μmol). Elemental analysis calcd (%) for $\mathbf{C_{37}H_{33}Cu_2F_9N_8O_{11}S_3}$ + 1 H_2O: C 38.25, H 3.04, N 9.64; found: C 38.15, H 2.97, N 9.61.

^{1}H NMR (400 MHz, D_2O, 298 K): δ = 70.3 (s, 4H), 65.9 (s, 4H), 59.9 (s, 4H), 49.3 (s, 4H), 21.5 (s, 4H), 19.9 (s, 4H), 13.9 (s, 2H), 11.5 (s, 2H), 9.4 (s, 4H) ppm.

^{19}F{^{1}H} NMR (400 MHz, D_2O, 298 K): δ = -78.9 ppm.

^{1}H NMR (400 MHz, CD_2Cl_2, 298 K): δ = 66.7 (s, 4H), 61.1 (s, 4H), 59.6 (s, 4H), 46.5 (s, 4H), 20.5 (s, 4H), 19.1 (s, 4H), 13.9 (s, 2H), 11.6 (s, 2H), 9.3 (s, 4H), -70.9 (br, 1H) ppm.

^{19}F{^{1}H} NMR (400 MHz, CD_2Cl_2, 298 K): δ = -78.6 ppm.

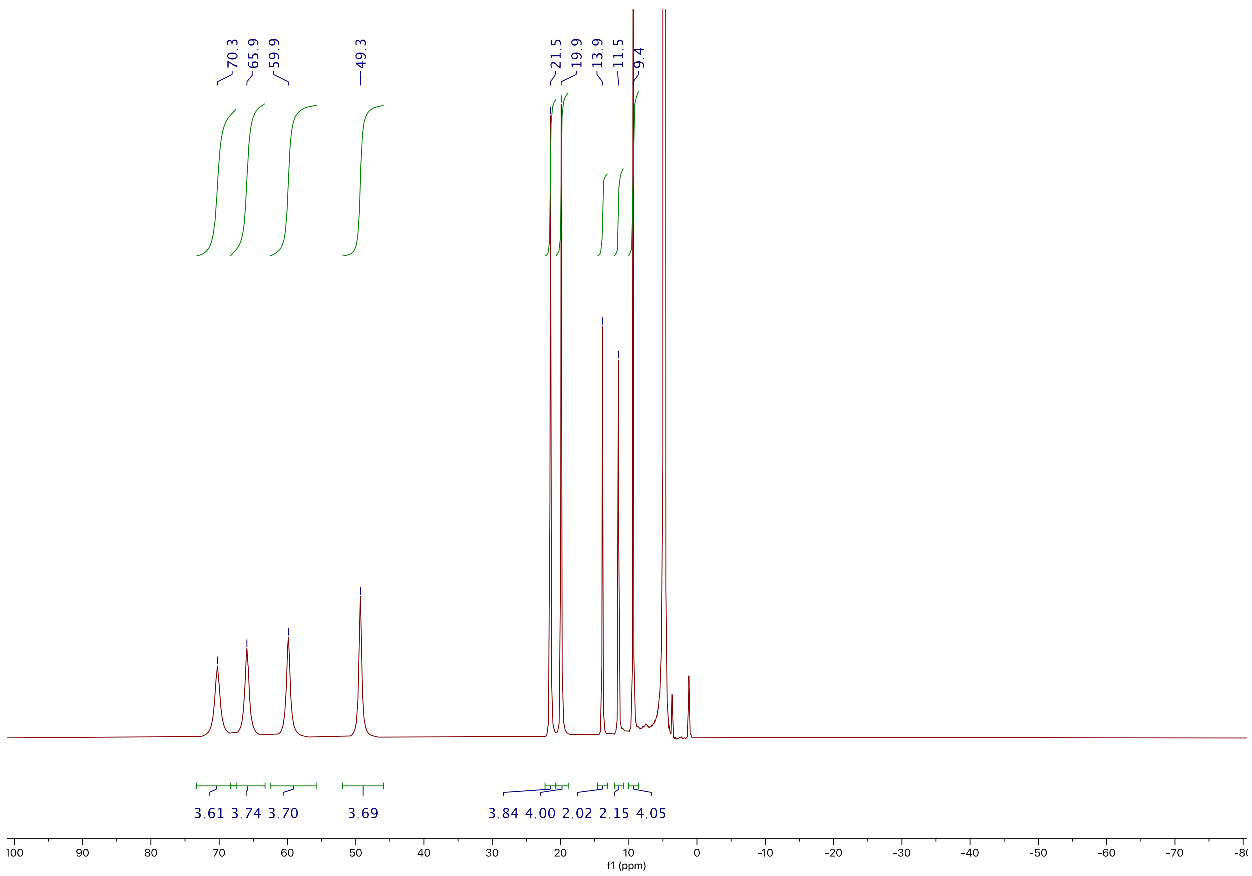

Figure S3 The ^{1}H NMR spectrum of **[BPMANCu$_2$(μ-OH)](OTf)$_3$** in D_2O at 25 ℃

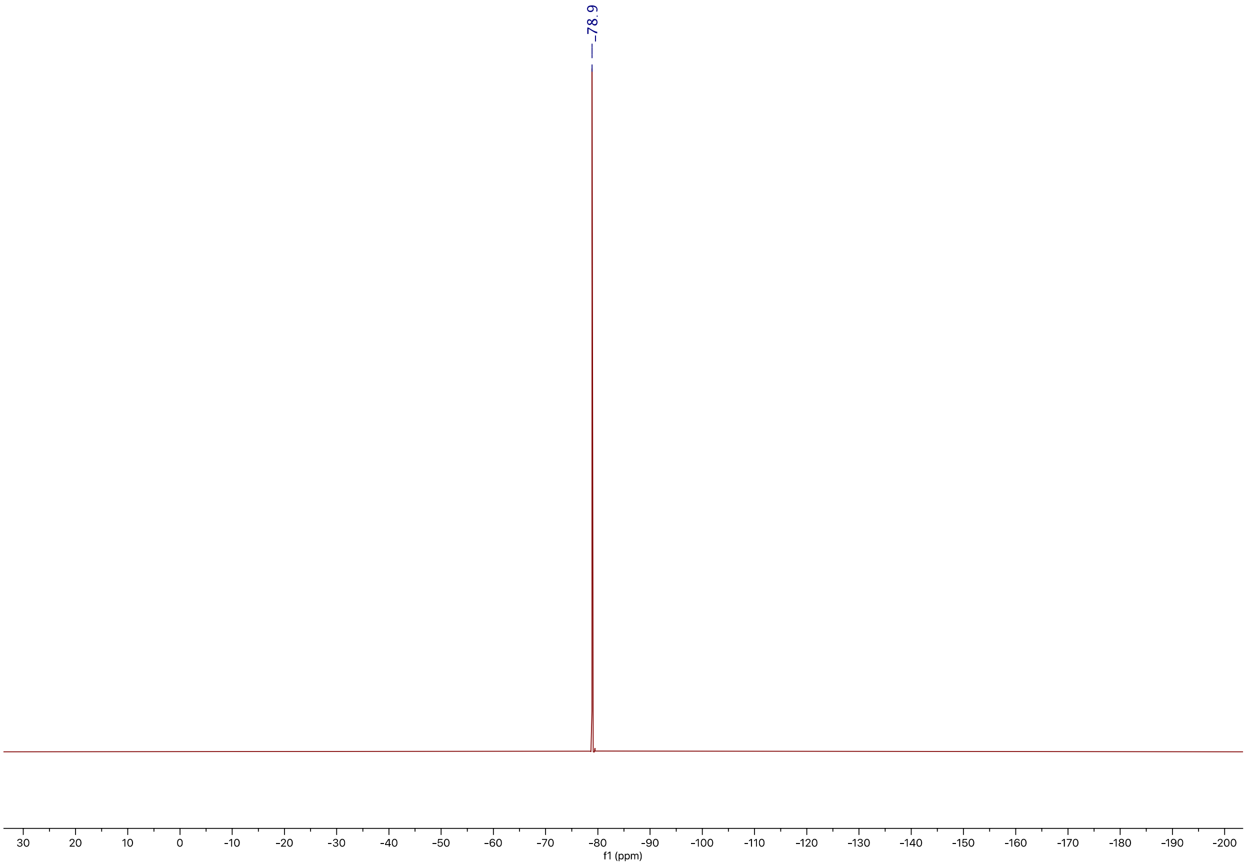

Figure S4 The ^{19}F NMR spectrum of **[BPMANCu$_2$(μ-OH)](OTf)$_3$** in D_2O at 25 ℃

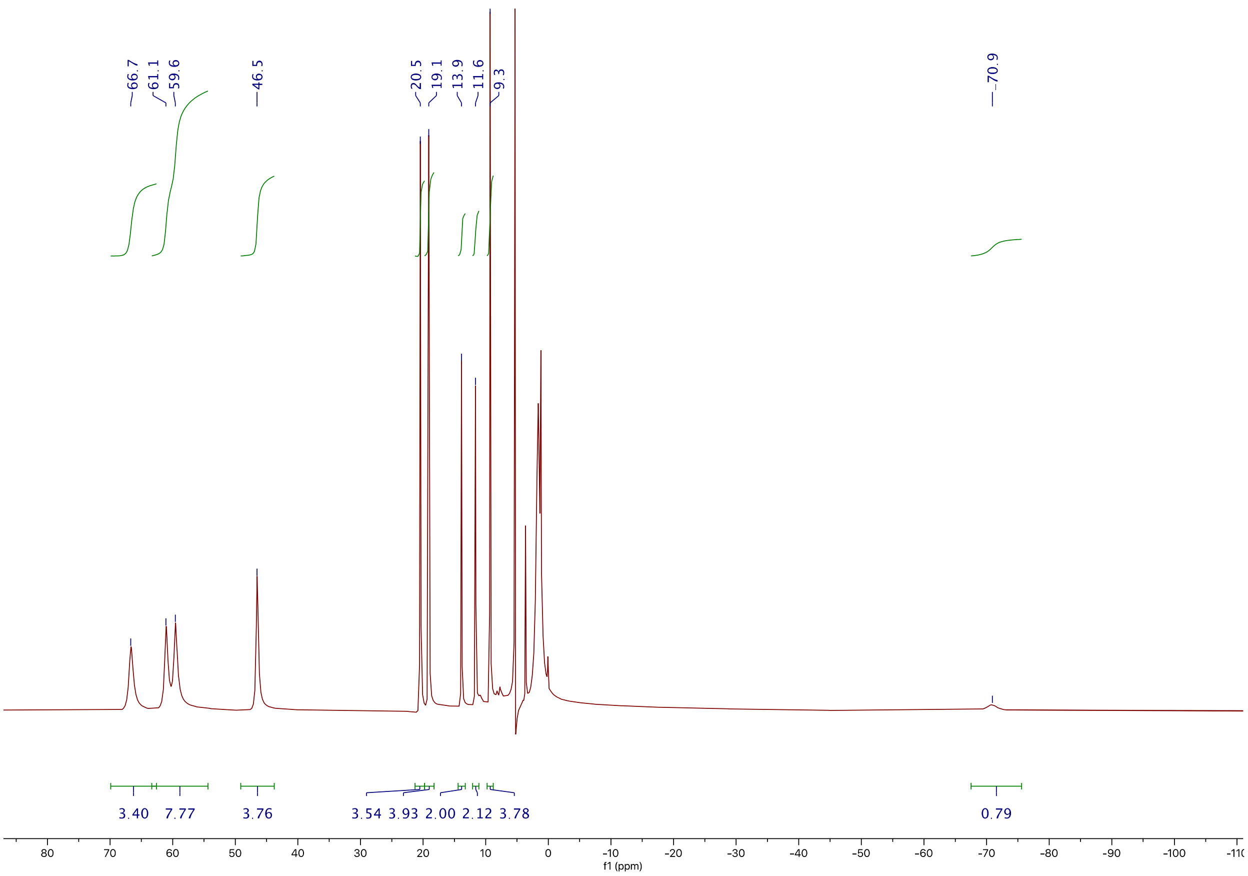

Figure S5 The ^{1}H NMR spectrum of **[BPMANCu$_2$(μ-OH)](OTf)$_3$** in CD_2Cl_2 at 25 ℃

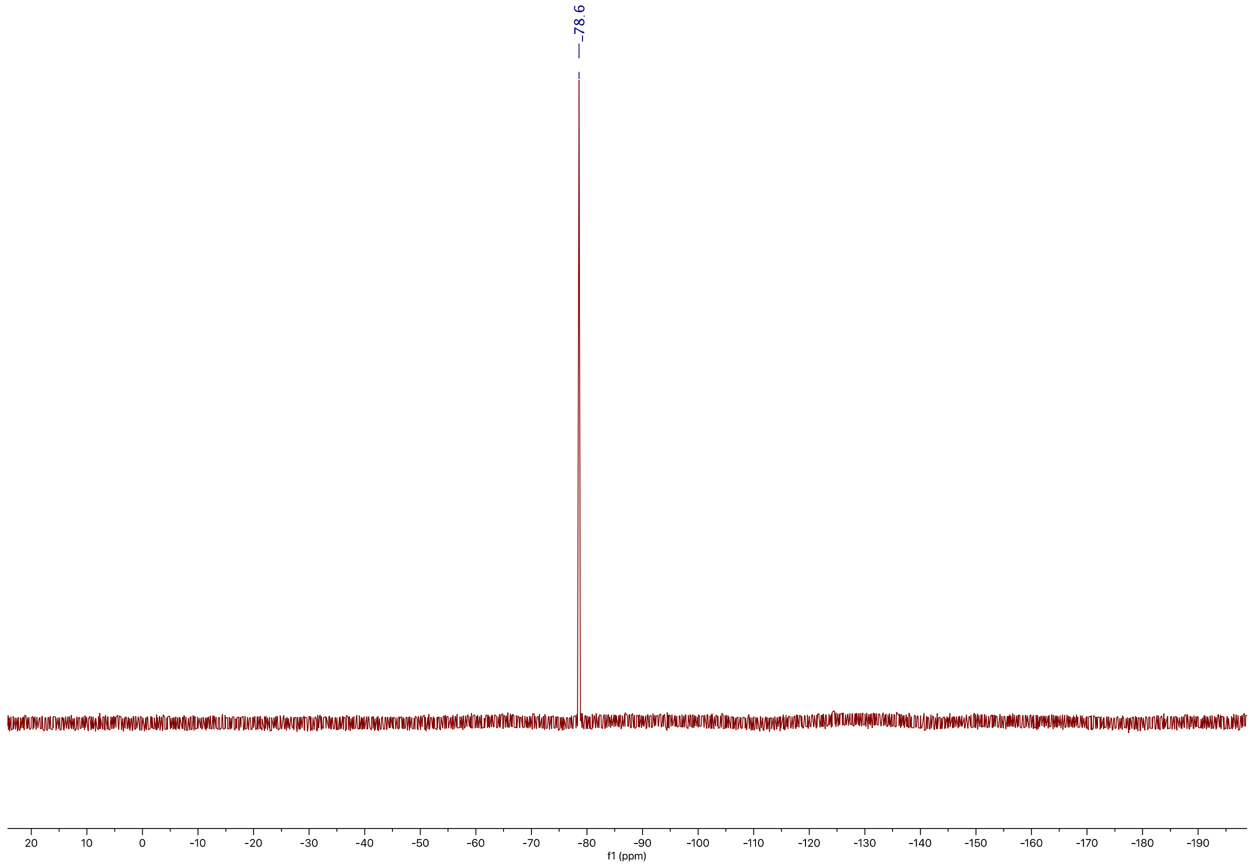

Figure S6 The ^{19}F NMR spectrum of **[BPMANCu$_2$(μ-OH)](OTf)$_3$** in CD_2Cl_2 at 25 ℃

2.4 Synthesis of [BPMANCu$_2$](OTf)$_2$

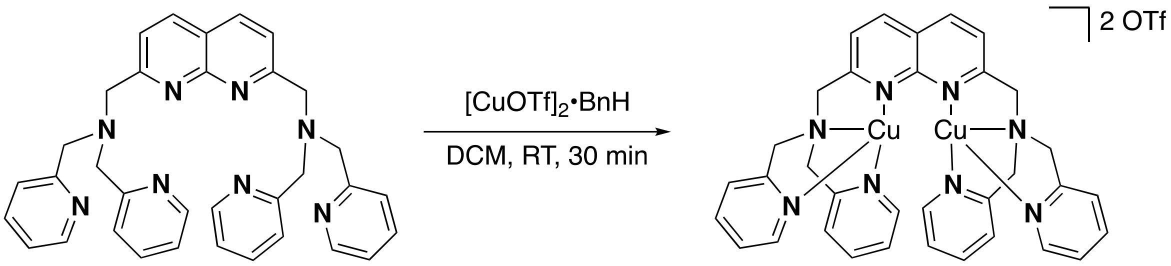

The compound was synthesized according to a modified literature procedure.[4] A solution of BPMAN (100.0 mg, 181 µmol) in DCM (2 mL) was added dropwise to a stirring suspension of [CuOTf]$_2$•BnH (93.6 mg, 181 µmol) in DCM (2 mL), turning the reaction mixture to a dark color. After stirring at ambient temperature for 30 min, the mixture was filtered over pipette filter and the filtrate was layered with Et_2O, resulting in the formation of dark colored needles overnight. The needles were isolated, washed with Et_2O (5 x 1mL) and dried under vacuum to give the title compound in 91% yield (165 mg, 165 µmol). Data is consistent with the literature.[4]

^{1}H NMR (400 MHz, CD_2Cl_2, 298 K): δ = 8.37 (m, 6H), 7.83 (t, $^3J_{H,H}$ = 7.6 Hz, 4H), 7.58 (m, 6H), 7.18 (t, $^3J_{H,H}$ = 6.3 Hz, 4H), 4.71 (s, 4H), 4.51 (s, 8H) ppm.

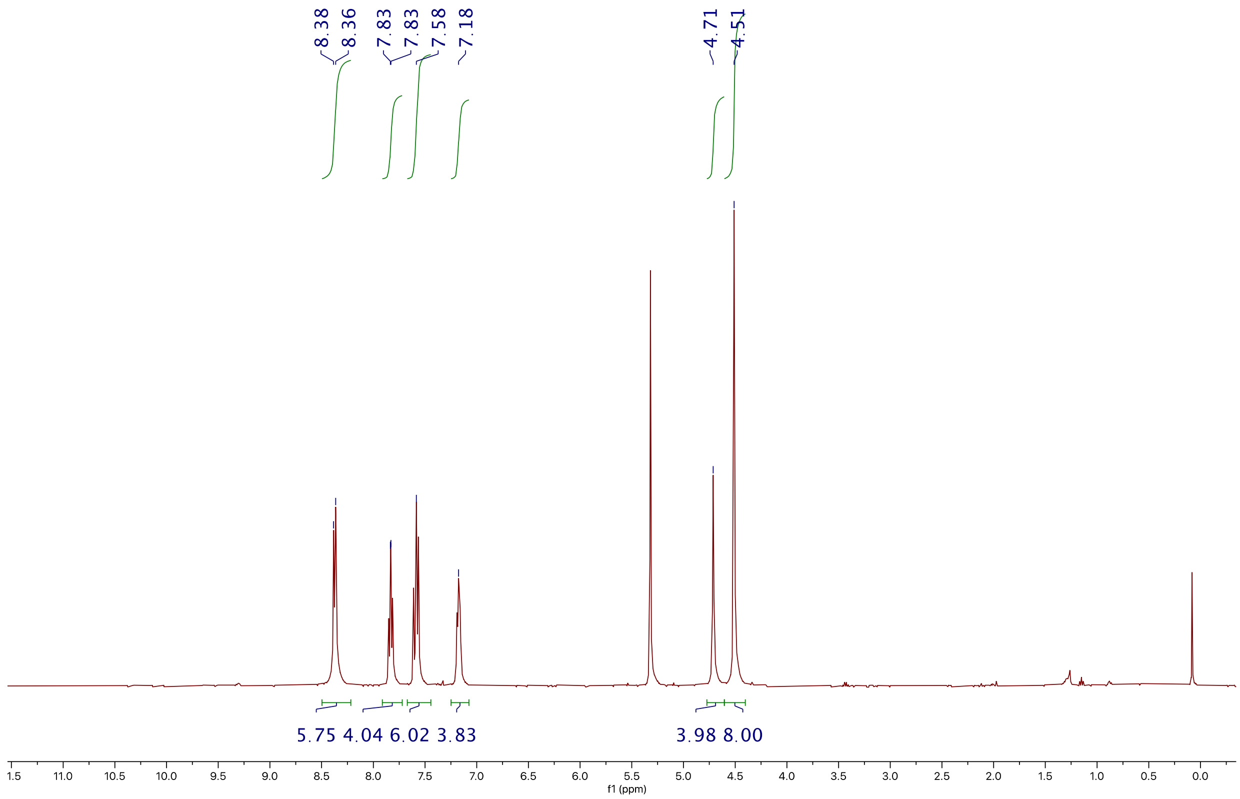

Figure S7 The ^{1}H NMR spectrum of **[BPMANCu$_2$](OTf)$_2$** in CD_2Cl_2 at 25 ℃

3. Hydrogen peroxide reduction by $[Cu_2L(\mu\text{-}OH)]^{3+}$

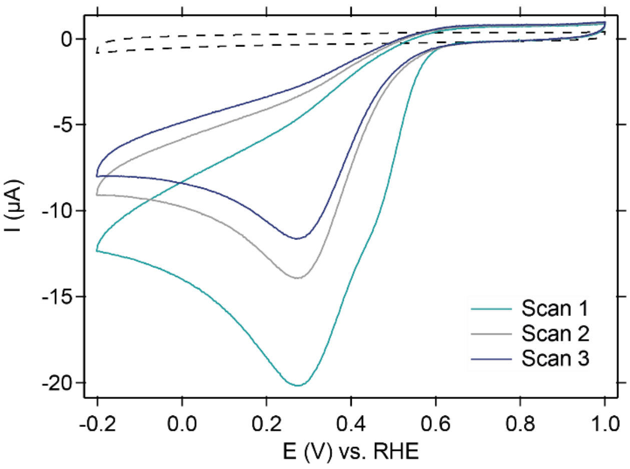

Figure S8 CV of the HPRR catalysed by $\mathbf{[Cu_2L(\mu\text{-}OH)]^{3+}}$. HPRR activity of the bare GC electrode shown as reference (dashed line). Conditions: 0.15 mM $\mathbf{[Cu_2L(\mu\text{-}OH)]^{3+}}$, 0.1 M PB pH 7, 1.1 mM H_2O_2, 293 K, 100 mV/s scan rate.

4. Catalyst stability during CV measurements

4.1 Deposit tests in CV

The possible formation of deposited species on the GC electrode and their electrocatalytic activity were studied. To do so, the same procedure was followed at all times:

1. A CV in a **solution of $[Cu_2L(\mu\text{-}OH)]^{3+}$** was measured under non-catalytic (Ar atmosphere) or catalytic conditions (O_2 atmosphere / 1.1 mM H_2O_2).
2. The electrode was thoroughly **rinsed** with Mili-Q water.
3. The **rinsed electrode** was transferred to a **blank solution** in the absence of catalyst and a CV was measured under non-catalytic (Ar atmosphere) or catalytic conditions (O_2 atmosphere / 1.1 mM H_2O_2).

In this manner, the experiments shown in **Table S1** were carried out and the results are discussed in the text hereafter.

Table S1 Overview of the experiments conducted to investigate the stability of **$[Cu_2L(\mu\text{-}OH)]^{3+}$** under non-catalytic and catalytic conditions.

**Experiment**	**Conditions catalyst cell**	**Conditions blank solution (no catalyst)**	**Main observation in blank solution**
**Figure S9a**	Non-catalytic conditions (Ar atmosphere), 0.3 mM catalyst, PB pH 7	Non-catalytic conditions (Ar atmosphere), PB pH 7	Small redox couple at the same position as **$[Cu_2L(\mu\text{-}OH)]^{3+}$**
**Figure S9b**	Catalytic conditions (O_2 atmosphere), 0.3 mM catalyst, PB pH 7	Non-catalytic conditions (Ar atmosphere), PB pH 7	Small redox couple at the same position as **$[Cu_2L(\mu\text{-}OH)]^{3+}$** + additional redox event around 0.1 V vs. RHE.
**Figure S9c**	Catalytic conditions (O_2 atmosphere), 0.3 mM catalyst, PB pH7	Catalytic conditions (O_2 atmosphere), PB pH 7	Deposit formed that is active for the ORR
**Figure S9d**	Catalytic conditions (1.1 mM H_2O_2), 0.3 mM catalyst, PB pH 7	Catalytic conditions (1.1 mM H_2O_2), PB pH 7	Deposit formed that is active for the HPRR
**Figure S10a**	Non-catalytic conditions (Ar atmosphere), 0.3 mM catalyst, PB pH7	Non-catalytic conditions (Ar atmosphere), Acetate Buffer pH 4.8 or PB pH7	Redox couple of the adsorbed species shifts in the same manner as the redox couple of **$[Cu_2L(\mu\text{-}OH)]^{3+}$** when the pH is changed
**Figure S10b**	Non-catalytic or catalytic conditions (Ar or O_2 atmosphere), 0.3 mM catalyst, PB pH7	Catalytic conditions (O_2 atmosphere), PB pH 7	Deposit formed under catalytic conditions is more active for the ORR

**Figure 11a**	Catalytic conditions (O_2 atmosphere), **2.0 μM catalyst**, PB pH 7	Catalytic conditions (O_2 atmosphere), PB pH 7	Formed deposit is clearly a lot more active than the homogeneous catalyst
**Figure 12**	Catalytic conditions (O_2 atmosphere), 0.3 mM catalyst, PB pH 7, **linear-sweep voltammetry (0.8 -0.1 V vs. RHE)**	Catalytic conditions (O_2 atmosphere), PB pH 7	The activity of the deposition that forms during linear-sweep voltammetry is low compared to the activity of the deposit that forms during 3 CV scans.

First, we investigated the possible formation of deposit on the electrode surface when CV scans of **[Cu$_2$L(μ-OH)]$^{3+}$** are recorded, both under non-catalytic and catalytic conditions. First, three CVs of **[Cu$_2$L(μ-OH)]$^{3+}$** were recorded under Ar atmosphere, and subsequently CV scans of the rinsed electrode were recorded in blank solution in absence of substrate. These measurements show that a deposit has formed on the electrode surface which has a redox couple at similar potentials as the **[Cu$_2$L(μ-OH)]$^{3+}$** redox couple (**Figure S9a**). This observation suggests adsorption of the catalyst onto the electrode surface. Next, six CV scans of a **[Cu$_2$L(μ-OH)]$^{3+}$** solution were recorded in presence of O_2, and subsequently CV scans of the rinsed electrode were recorded in blank solution under Ar atmosphere. These measurements show that a deposit has formed on the electrode surface which has a redox couple at similar potentials as the **[Cu$_2$L(μ-OH)]$^{3+}$** redox couple, but also additional redox events at lower potentials (~0.1 V vs. RHE) are observed (**Figure S9b**). It is hypothesized that in this case the catalyst partly decomposes on the electrode surface during catalysis, giving rise to these additional redox events.

Next, the activity of the formed deposits for the ORR and HPRR was further analyzed. To do so, CV measurements of **[Cu$_2$L(μ-OH)]$^{3+}$** were recorded in presence of O_2, whereafter the rinsed electrode was transferred to a blank solution under O_2 atmosphere. These measurements indicate that the deposit that forms during catalysis of the ORR by the catalyst, is in turn an active catalyst for the ORR (**Figure S9c**). In a similar way, CV scans of **[Cu$_2$L(μ-OH)]$^{3+}$** in presence of H_2O_2 were recorded, and the rinsed electrode was transferred to a blank solution containing H_2O_2. This measurement indicates that during catalysis of the HPRR by the catalyst, a catalytically active deposit is formed on the surface of the electrode (**Figure S9d**). The deposited species that forms during HPRR behaves differently from the homogeneous **[Cu$_2$L(μ-OH)]$^{3+}$** in solution, as the onset potential of the catalytic wave shifts. Taken together, the experiments in **Figure S9** suggest that a catalytically active, heterogeneous deposit forms on the electrode during catalysis of the ORR and HPRR by **[Cu$_2$L(μ-OH)]$^{3+}$**, whilst the catalyst only

adsorbs onto the electrode during CV measurements under Ar atmosphere. This hypothesis was further investigated in additional experiments below.

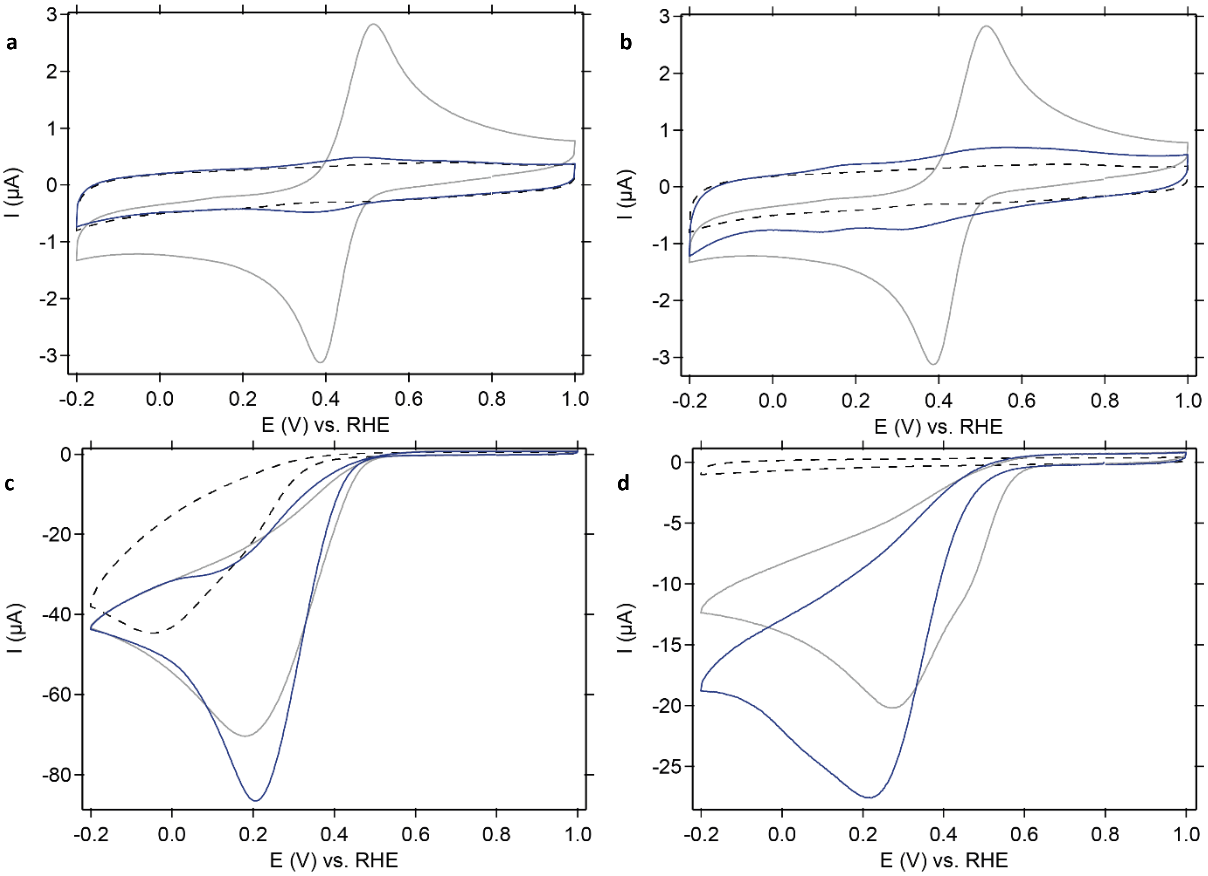

Figure S9 Deposit tests of electrochemical measurements with **[Cu$_2$L(µ-OH)]$^{3+}$**. **a)** CVs under Ar atmosphere of the deposit formed on the electrode after recording 3 scans of the **[Cu$_2$L(µ-OH)]$^{3+}$** redox couple (dark blue) compared to the **[Cu$_2$L(µ-OH)]$^{3+}$** redox couple itself (grey) and the bare GC electrode (black dashed line). **b)** CV under Ar atmosphere ofthe deposit formed on the electrode after 6 ORR scans of **[Cu$_2$L(µ-OH)]$^{3+}$** (dark blue), compared to the **[Cu$_2$L(µ-OH)]$^{3+}$** redox couple itself (grey) and the bare GC electrode (black dashed line). **c)** CV under O_2 atmosphere of the deposit formed after 3 ORR scans of **[Cu$_2$L(µ-OH)]$^{3+}$** (dark blue), compared to the ORR activity of homogeneous **[Cu$_2$L(µ-OH)]$^{3+}$** (grey) and the bare GC electrode (black dashed line). **d)** CVs in presence of 1.1 mM H_2O_2 of deposit formed on the electrode after 3 HPRR scans (40 mM H_2O_2) of **[Cu$_2$L(µ-OH)]$^{3+}$** recorded (dark blue) compared to the HPRR activity of homogeneous **[Cu$_2$L(µ-OH)]$^{3+}$** in presence of 1.1 mM H_2O_2 (grey) and the bare GC electrode. Conditions: 0.15 mM **[Cu$_2$L(µ-OH)]$^{3+}$**, 0.1 M PB pH 7, Ar or O_2 atmosphere or 1.1 mM H_2O_2, 293 K, 100 mV/s scan rate.

Next, experiments were carried out to investigate if the deposit that forms on the electrode during CVs scans of **[Cu$_2$L(µ-OH)]$^{3+}$** under Ar atmosphere, is indeed the adsorbed catalyst. To so, CV measurements of **[Cu$_2$L(µ-OH)]$^{3+}$** were recorded in absence of substrate, whereafter CV scans of the rinsed electrode were recorded in a blank solution of two different buffers. Subsequently, the CVs of the rinsed electrode were

compared to CVs of the redox couple **[$Cu_2L(\mu$-OH)]$^{3+}$** in these two buffered solutions. If the redox couple of the deposit on the electrode belongs to **[$Cu_2L(\mu$-OH)]$^{3+}$**, both redox couples should shift in a similar way upon changing the pH of the solution. In line with this hypothesis, **Figure S10a** shows that both the redox couple of **[$Cu_2L(\mu$-OH)]$^{3+}$** and of the deposited species shift to more positive potentials when the buffer is changed from phosphate buffer pH 7 to acetate buffer pH 4.85.

As it is evident that the catalyst adsorbs on the electrode during CV measurements under Ar atmosphere, we further investigated if the catalyst does not also decompose. To do so, CVs of a **[$Cu_2L(\mu$-OH)]$^{3+}$** solution under Ar or O_2 atmosphere were recorded. Subsequently, CV scans of the rinsed electrode were recorded in a blank solution in the presence of O_2. From **Figure S10b** it is evident that the deposit that formed on the GC electrode during measurements of **[$Cu_2L(\mu$-OH)]$^{3+}$** under Ar atmosphere is only slightly more active than the bare GC electrode. Together with the observation that the redox couple of the deposit is only small compared to the homogeneous catalyst in solution (see **Figure S9a**), this is an indication that when **[$Cu_2L(\mu$-OH)]$^{3+}$** is studied under Ar atmosphere only a small amount of catalyst adsorbs on the electrode. On the contrary, the deposit that forms on the electrode during catalysis of the ORR by **[$Cu_2L(\mu$-OH)]$^{3+}$** is clearly more catalytically active than the bare GC electrode (see **Figure S10**b).

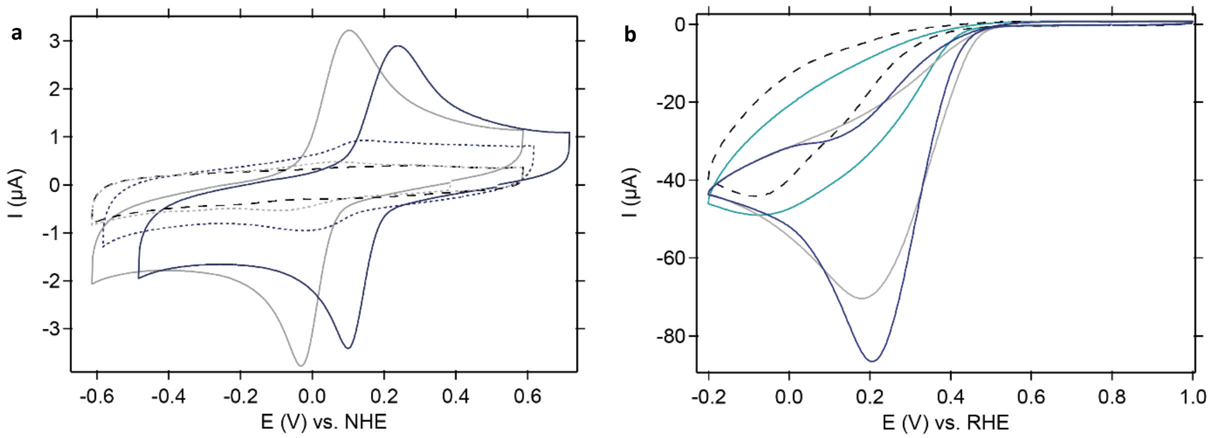

Figure S10 Deposit tests of electrochemical measurements with **[$Cu_2L(\mu$-OH)]$^{3+}$**. **a)** CVs under Ar atmosphere of the GC electrode (dotted lines) after 6 scans of the **[$Cu_2L(\mu$-OH)]$^{3+}$** redox couple, measured in PB pH 7 (dark blue) and acetate buffer pH 4.8 (grey). CV of homogeneous catalyst in solution (solid lines) and the bare GC electrode in PB under Ar atmosphere shown for comparison (black dashed line). **b)** CVs under O_2 atmosphere of the GC electrode after 3 scans of the **[$Cu_2L(\mu$-OH)]$^{3+}$** redox couple (light blue line) and after 3 scans of the ORR activity of **[$Cu_2L(\mu$-OH)]$^{3+}$** (dark blue). ORR activity of the homogeneous catalyst in solution (grey line) and of the bare GC electrode (black dashed line) shown for comparison. Conditions: 0.15 mM **[$Cu_2L(\mu$-OH)]$^{3+}$**, 0.1 M PB pH 7 or 0.1 M acetate buffer pH 4.8, Ar or O_2 atmosphere, 293 K, 100 mV/s scan rate.

Next, we investigated the ORR activity of the deposit that forms on the electrode surface when **[$Cu_2L(\mu$-OH)]$^{3+}$** is measured in presence of O_2. These CV measurements were recorded at low catalyst

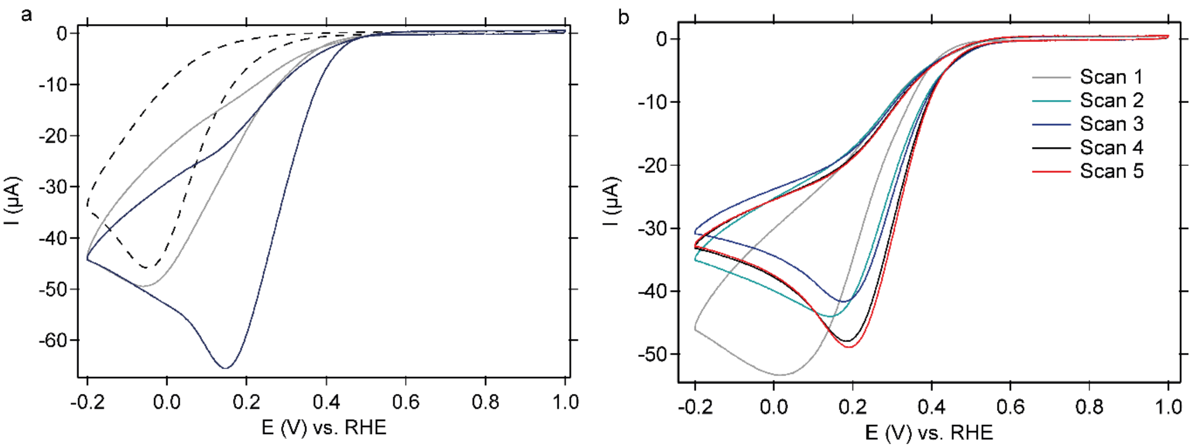

Figure S11 a) Deposit tests of electrochemical measurements with **[Cu_2L(μ-OH)]$^{3+}$**. CV in O_2 atmosphere of the GC electrode after 3 ORR scans of **[Cu_2L(μ-OH)]$^{3+}$** (dark blue), compared to the ORR activity of homogeneous **[Cu_2L(μ-OH)]$^{3+}$** (grey line) and the bare GC electrode (black dashed line). **b)** 5 CV scans of **[Cu_2L(μ-OH)]$^{3+}$** under O_2 atmosphere. Conditions: 2.0 μM **[Cu_2L(μ-OH)]$^{3+}$**, 0.1 M PB pH 7, O_2 atmosphere, 293 K, 100 mV/s scan rate.

concentrations, as this will lead to non-substrate-limited conditions. Under these conditions, depletion of oxygen will not limit the catalytic current that is recorded, making it is possible to accurately compare the catalytic activity of the **[Cu_2L(μ-OH)]$^{3+}$** in solution and the formed deposit. CVs in a 2 μM **[Cu_2L(μ-OH)]$^{3+}$** solution under O_2 atmosphere were recorded, and subsequently a CV of the rinsed electrode was recorded in a blank solution in the presence of O_2. **Figure S11a** shows the catalytic current of the deposit that formed on the electrode during catalysis and compares this to the catalytic current of a 2 μM **[Cu_2L(μ-OH)]$^{3+}$** solution. From this figure it is evident that the formed deposit has a much higher catalytic activity than the homogeneous catalyst in solution. If the heterogeneous deposits would have formed immediately upon recording a CV of **[Cu_2L(μ-OH)]$^{3+}$**, a catalytic activity that more closely resembles that of the deposit would be expected. In addition, **Figure S11b** shows 5 CV scans of a 2 μM **[Cu_2L(μ-OH)]$^{3+}$** solution under O_2 atmosphere. This shows that in 5 consecutive scans the shape of the CV changes to a CV with a sharper and earlier peak current, which indicates that a catalytic active deposit forms on the surface of the electrode. Subsequently, an experiment was conducted to analyze any contribution of catalytically active deposit during measurements of the homogeneous catalyst in solution. In this case, a linear sweep voltammogram of **[Cu_2L(μ-OH)]$^{3+}$** solution under O_2 atmosphere was recorded between 0.8 and 0.1 V vs. RHE and the rinsed electrode was transferred to a blank solution in presence of O_2 (**Figure S12**). From this measurement it is clear that the deposit that forms on the electrode during the linear sweep voltammetry is less active than the homogeneous catalyst in solution and is also less active than the deposit that forms in a similar experiment over 3 CV scans (See **Figure S9c**). This observation indicates that the contribution

of deposit is minimal in the first part of the catalytic CV measurement of the homogeneous catalyst and is an indication that the deposit builds up over multiple CV scans.

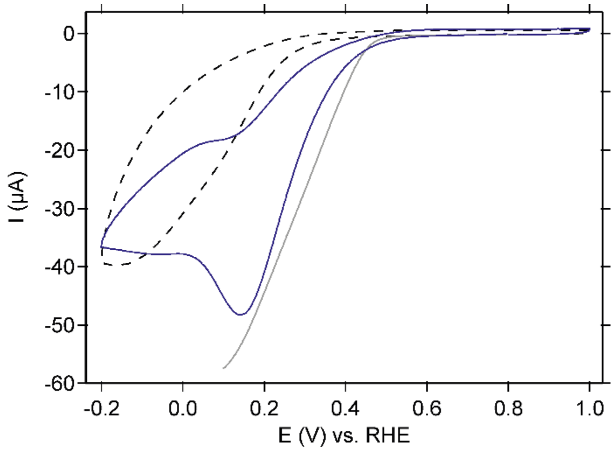

Figure S12 Linear sweep voltammetry of the ORR activity of **[Cu_2L(µ-OH)]$^{3+}$** (grey line) compared to the ORR activity of the GC electrode after recording this linear sweep voltammetry (dark blue). ORR activity of the bare GC electrode (black dashed line) shown for comparison. Conditions: 0.15 mM **[Cu_2L(µ-OH)]$^{3+}$**, 0.1 M PB pH 7, O_2 atmosphere, 293 K, 100 mV/s scan rate.

4.2 Electrochemical quartz crystal microbalance measurements

Measurements using an electrochemical quartz crystal microbalance (EQCM) setup were used to further investigate the formation of deposits during CV measurements of **[Cu_2L(µ-OH)]$^{3+}$** under Ar and O_2 atmosphere. The EQCM system comprises of an oscillating quartz crystal that is gold-coated. During an electrochemical experiment any mass change of this working electrode directly corresponds to a frequency change of the vibrating quartz crystal. In this way, the formation of deposits on the gold surface can be measured as a function of frequency change (ΔHz). In EQCM experiments the frequency decreases when the mass of the electrode increases, while the frequency increases when the mass of the electrode decreases. EQCM measurements of **[Cu_2L(µ-OH)]$^{3+}$** under argon show that no detectable deposits are formed under these conditions (**Figure S13c**). The small frequency change of <10 Hz is comparable to the frequency change measured in the absence of catalyst (**Figure S13a-b**), and can also indicate the adsorption of catalyst on the electrode. The EQCM experiments of **[Cu_2L(µ-OH)]$^{3+}$** under ORR conditions show a clear build-up of mass on the electrode (**Figure S13d**), which slows down over the course of the experiment due to the depletion of oxygen near the electrode. In general, the EQCM measurements confirm the trends observed in the deposit tests. In addition, from the first scan **Figure S13d** (dark blue trace) it is evident that the largest change in frequency occurs when the catalytic wave reaches a potential

below 0.2 V vs RHE. It is therefore assumed that most deposits are formed after the peak in the catalytic current and hence, the catalytic activity of the first scan is not influenced by active deposits that can form.

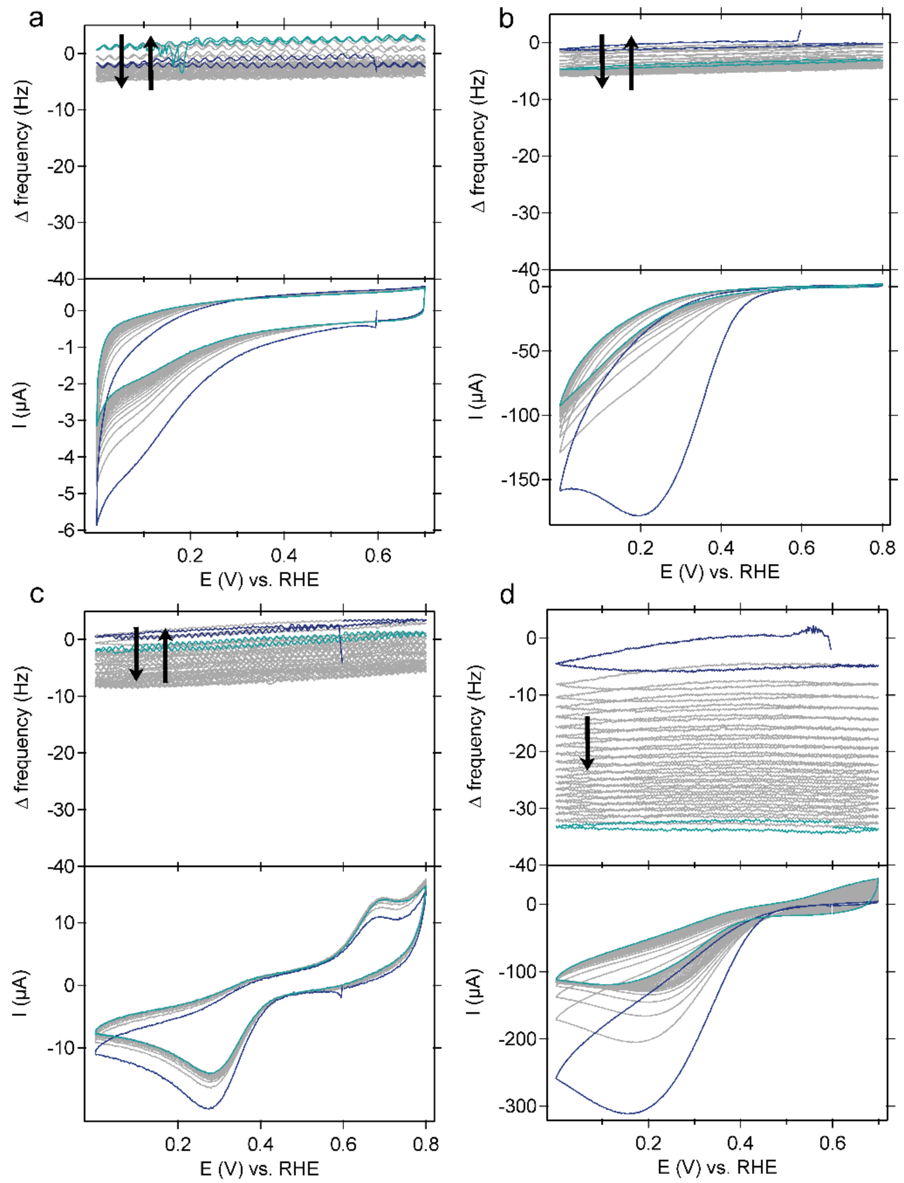

Figure S13 EQCM measurements showing CVs (bottom) and corresponding frequency changes (top) for **a)** a bare gold electrode under Ar atmosphere, **b)** a bare gold electrode under O_2 atmosphere, **c)** 0.15 mM **[Cu$_2$L(μ-OH)]$^{3+}$** under Ar atmosphere, and **d)** 0.15 mM **[Cu$_2$L(μ-OH)]$^{3+}$** under O_2 atmosphere. Scan 1 = dark blue trace, scan 20 = light blue trace. Arrows indicate the frequency change over 20 scans and show that the frequency oscillates in a 10 Hz window in case of a, b and c, and significantly increases in d. Conditions: 0.1 M PB pH 7, Ar or O_2 atmosphere, 293 K, 100 mV/s scan rate.

5. Scan rate dependence and diffusion coefficient calculations

The **$[Cu_2L(\mu\text{-}OH)]^{3+}$** redox couple was recorded at varying scan rates between 10 mV/s and 500 mV/s (**Figure S14a**) in a randomized order that allows disentanglement of time and scan rate dependent processes. The diffusion coefficient of **$[Cu_2L(\mu\text{-}OH)]^{3+}$** and its reduced form can be calculated from the slopes of the Randles-Sevcik plot (**Figure S14b**) using the background-corrected peak currents (I_p), and **Equation 1** where *n* is the number of electrons transferred (*n* = 2), T is the temperature (T = 293 K), A is the surface of the electrode (A = 0.0707 cm^2), C^0 is the catalyst concentration in the bulk, and v is the scan rate. The diffusion coefficients were determined to be 7.3 × 10^{-7} cm^2/s for the oxidized species, and 9.6 × 10^{-7} cm^2/s for the reduced species. The peak current corresponding to the reduction of **$[Cu_2L(\mu\text{-}OH)]^{3+}$** at low catalyst concentrations can be derived from equation 1 and the calculated diffusion coefficient.

$$i_p = 0.446nFAC^0\left(\frac{nFvD}{RT}\right)^{\frac{1}{2}} \qquad \textbf{(1)}$$

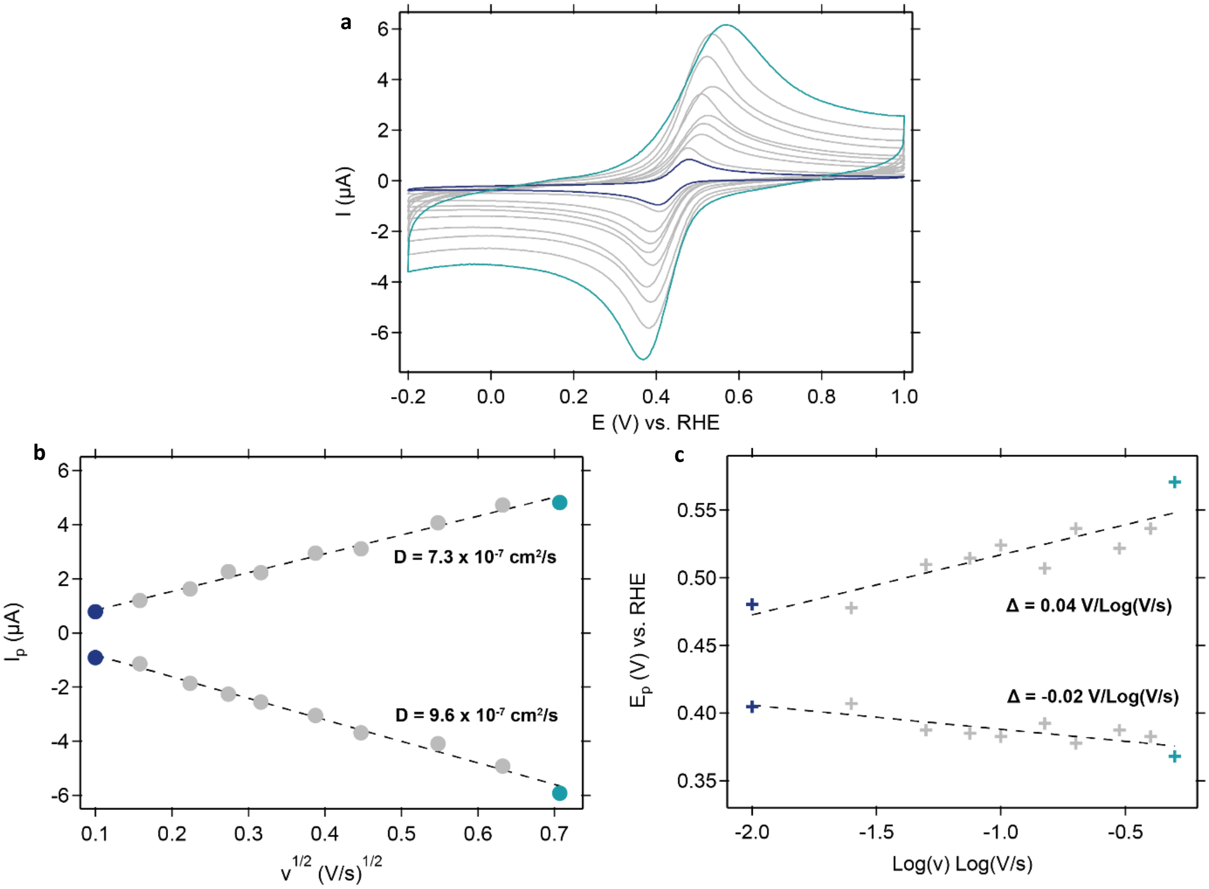

Figure S14 a) Scan rate dependence of the **$[Cu_2L(\mu\text{-}OH)]^{3+}$** redox couple at varying scan rates between 10 mV/s (dark blue) and 500 mV/s (light blue). **b)** Randles-Sevcik plot of the anodic (top, $R^2 = 0.99$) and cathodic (bottom, $R^2 = 0.99$) peak currents of **$[Cu_2L(\mu\text{-}OH)]^{3+}$** as a function of the square root of the scan rate. **c)** Laviron plot of the anodic (top) and cathodic (bottom) peak potentials of the **$[Cu_2L(\mu\text{-}OH)]^{3+}$** redox couple. Conditions: 0.1 M PB pH 7, Ar atmosphere, 293 K, 0.15 mM **$[Cu_2L(\mu\text{-}OH)]^{3+}$**.

6. pH dependence studies

6.1 CV measurements

The CV response of **[Cu₂L(μ-OH)]³⁺** was measured in a buffered Britton-Robinson solution over a wide pH range (See **Figure S15**a). A corresponding plot of the $E_{1/2}$ of these CV scans as a function of the pH is shown in **Figure S15b**. In general, the data points can be reproduced upon changing the pH from low to high and back.As seen in this figure, four points clearly resulted in a species with a very different $E_{1/2}$ value than expected. For clarity, these points were not included in the differential pulse voltammetry (DPV) analysis discussed below.

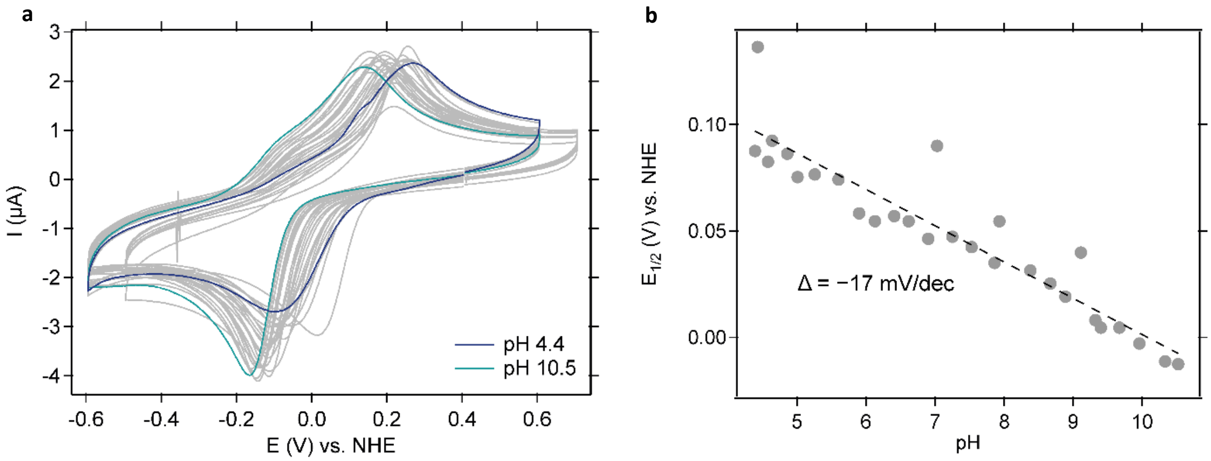

Figure S15 a) CV measurements of the **$[Cu_2L(\mu\text{-}OH)]^3$** redox couple at varying pH values and b) the corresponding $E_{1/2}$ values found for the **$[Cu_2L(\mu\text{-}OH)]^3$** redox couple as a function of pH, including the slope of the graph. Conditions: 0.15 mM **$[Cu_2L(\mu\text{-}OH)]^{3+}$**, 0.01 M Britton-Robinson buffer and 0.05 M Na_2SO_4, Ar atmosphere, 293 K, 100 mV/s scan rate.

6.2 DPV measurements

DPV measurements of the anodic and cathodic waves of **$[Cu_2L(\mu\text{-}OH)]^{3+}$** were recorded in Britton-Robinson buffer of different pH values (See **Figure S16**). The reduction peak shifts to more negative potentials upon changing the pH of the solution from low to high. In case of the oxidation, two peaks are observed. At low pH, these two peaks have approximately the same intensity, and their overlap causes the formation of a third peak in between the other two peaks at certain pH values. Upon changing the pH from low to high, the oxidation peak at high potential decreases, while the peak at lower potential increases and shifts to more negative potentials. This observation indicates the presence of an equilibrium between two different oxidation events that are strongly dependent on the pH. The oxidation peak at lower potential can be found at approximately the same potential as the reduction peak, indicating that these peaks belong to the reduction and oxidation of the same species.

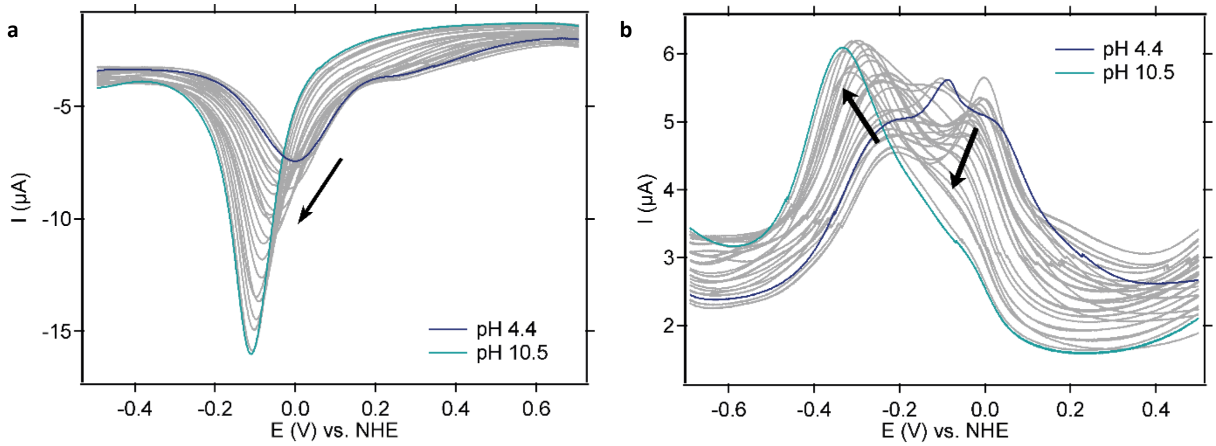

Figure S16 DPV measurements of the **a)** reduction and **b)** oxidation of **$[Cu_2L(\mu\text{-}OH)]^3$** at varying pH. The arrows indicate how the shape of the peak changes from low to high pH. Conditions: 0.15 mM **$[Cu_2L(\mu\text{-}OH)]^{3+}$**, 0.01 M Britton-Robinson buffer and 0.05 M Na_2SO_4, Ar atmosphere, 293 K, regular voltage pulses of 3 mV, a step potential of 0.3 mV, a modulation time of 3 ms, and a time interval of 50 ms.

6.3 UV-vis at varying pH

UV-vis spectra of a **$[Cu_2L(\mu\text{-}OH)]^{3+}$** solution at varying pH were recorded (see **Figure S17**). A 50 mL solution of 0.025 mM **$[Cu_2L(\mu\text{-}OH)]^{3+}$** in 0.1 M Na_2SO_4 was stirred vigorously. The pH of the solution was adjusted by addition of small quantities of 0.1 or 0.01 mM NaOH and H_2SO_4 solutions. The pH of the solution was determined prior to every UV-vis measurement. Between pH 7.9 and pH 2.8 the peak around 350 nm disappears, which reappears upon increasing the pH again.

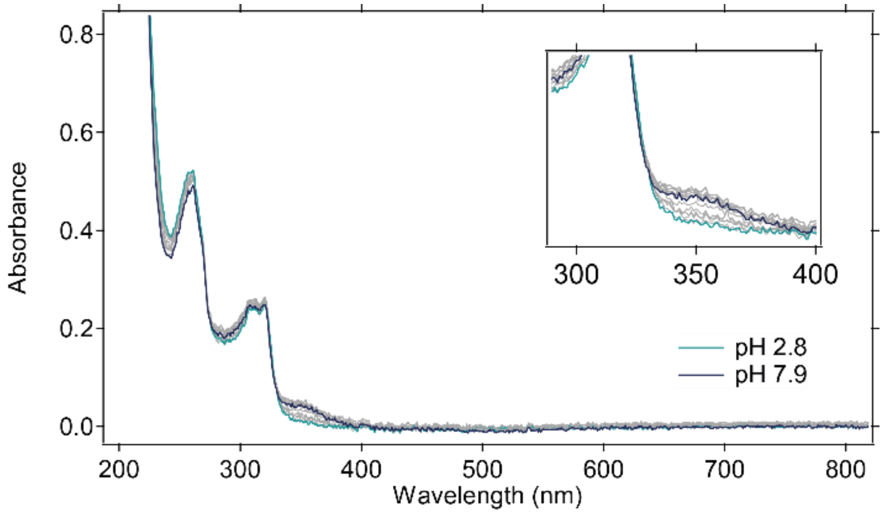

Figure S17 UV-vis spectrum of a 0.025 mM **$[Cu_2L(\mu\text{-}OH)]^{3+}$** solution in 0.1 M Na_2SO_4 between pH 3.3 (dark blue) and pH 10 (light blue) at 25 °C. The inset shows the peak around 350 nm.

7. Reactivity studies

7.1 Air exposure experiment with $[Cu_2L]^{2+}$

A solution of **$[Cu_2L](OTf)_2$** (5.4 mg, 5.5 µmol) in DCM (0.6 mL), containing a calibrated cobaltocene (in benzene) capillary as internal standard, was exposed to air. The initial yellowish solution turned green first and then progressively to a dark orange color. ^1H NMR spectra (paramagnetic settings) were collected after 2 minutes, 1 hour, 2 hours and 20.5 hours showing initial formation of multiple species including the **$[Cu_2L(\mu\text{-}OH)]^{3+}$** cation, with the latter being the only species observed after 20.5 h in 29% spectroscopic yield (**Figure S18**). UV-vis spectra recorded during the first 60 minutes of the experiment indicate the formation of a new species that has a Cu(II) oxidation state (**Figure S19**).

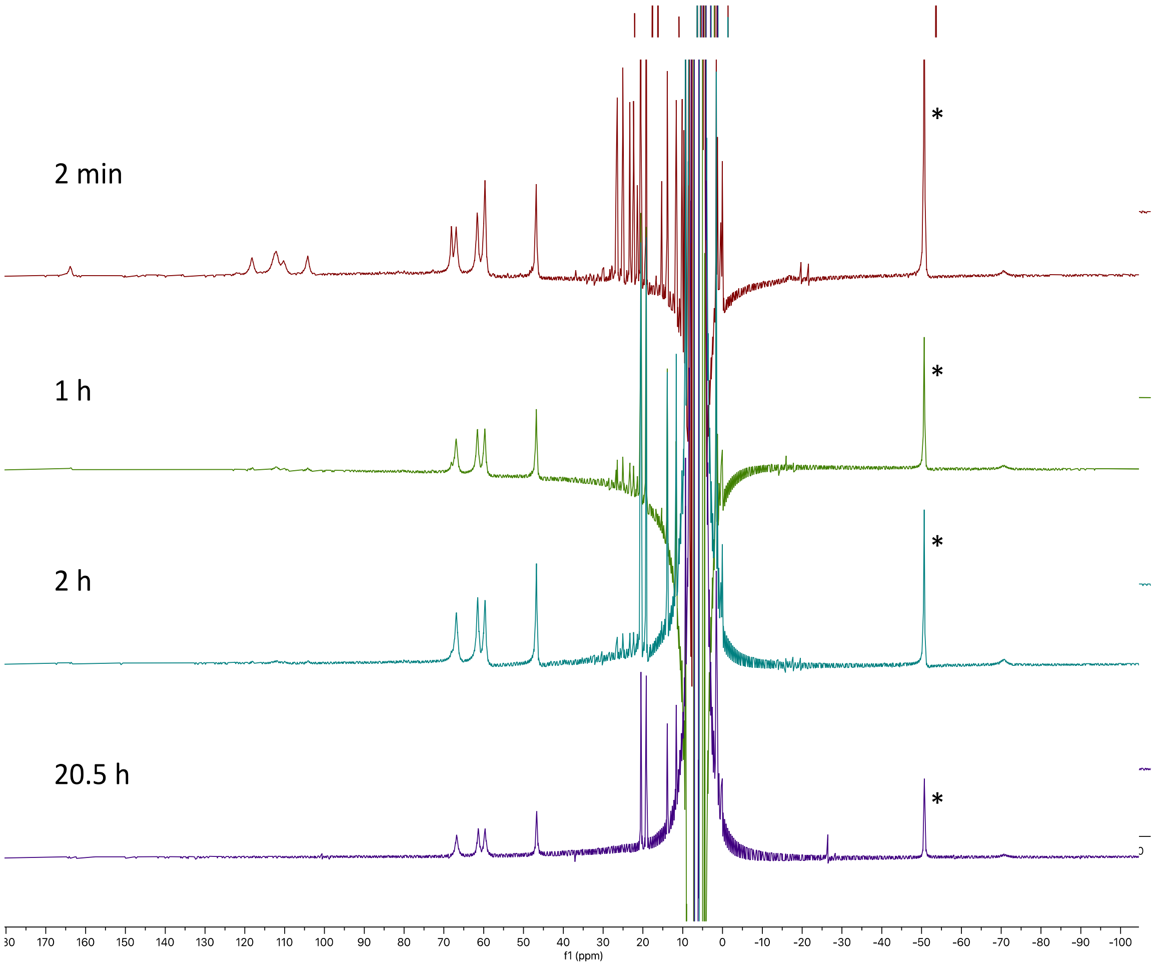

Figure S18 Stacked ^1H NMR (paramagnetic settings) spectra of the exposure of **$[Cu_2L](OTf)_2$** in DCM to air at 25 °C after 2 min, 1 h, 2h and 20.5 h. The asterisk marked resonance belongs to the cobaltocene (benzene solution in a capillary) internal standard.

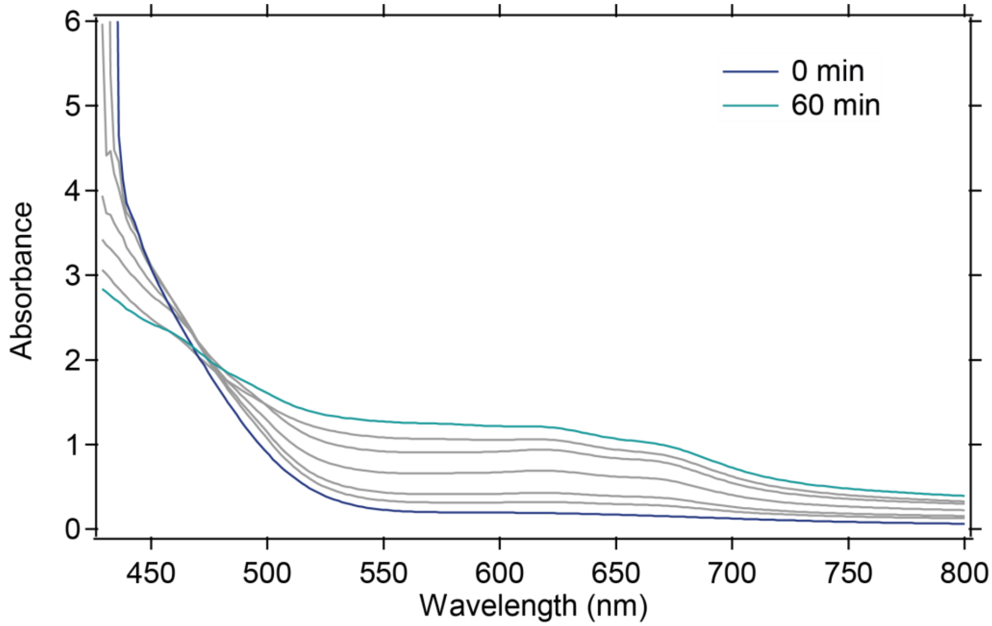

Figure S19 UV-vis spectra of the exposure of **$[Cu_2L](OTf)_2$** in DCM to air at the beginning of the experiment (dark blue) and after every 10 minutes (grey) up to 60 minutes (light blue).

7.2 Experiment with $[Cu_2L]^{2+}$and PhIO

A slurry of iodosylbenzene (PhIO) (2.2 mg, 5.1 µmol) in DCM (0.6 mL) was added to a vial containing **$[Cu_2L](OTf)_2$** (5.0 mg, 5.1 µmol) and the mixture was stirred for 5 minutes until homogeneous, with the color progressing from yellow to dark orange. Subsequently the dark solution was transferred to a J Young's NMR tube containing a calibrated cobaltocene (in benzene) capillary as internal standard. ^{1}H NMR spectra (paramagnetic settings) were collected after 15 minutes, 1.5 hour, 4 hours and 21.5 hours showing the formation of the **$[Cu_2L(\mu\text{-}OH)]^{3+}$** cation in 14% spectroscopic yield (Note: Over the course of the reaction, dark, insoluble solids formed in the mixture) (**Figure S20**). After the last measurement, the volatiles were removed to yield 6.6 mg of a dark orange film. A UV-vis spectrum of the reaction mixture was recorded at the end of the experiment, indicating the presence of a Cu(II) species (**Figure S21**).

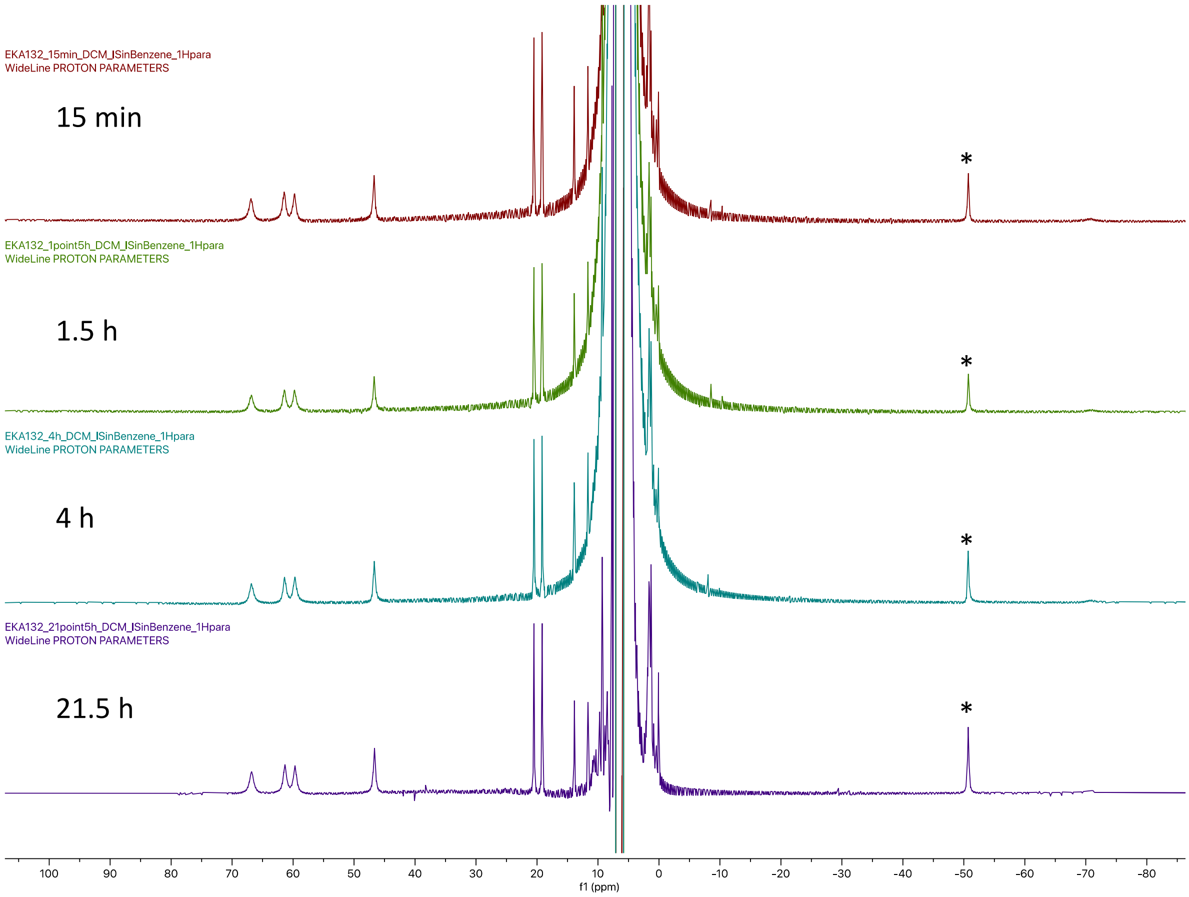

Figure S20 Stacked ^{1}H NMR (paramagnetic settings) spectra of the reaction between **[Cu_2L](OTf)$_2$** and PhIO in DCM at 25 ℃ after 15 min, 1.5 h, 4h and 21.5 h. The asterisk marked resonance belongs to the cobaltocene (benzene solution in a capillary) internal standard.

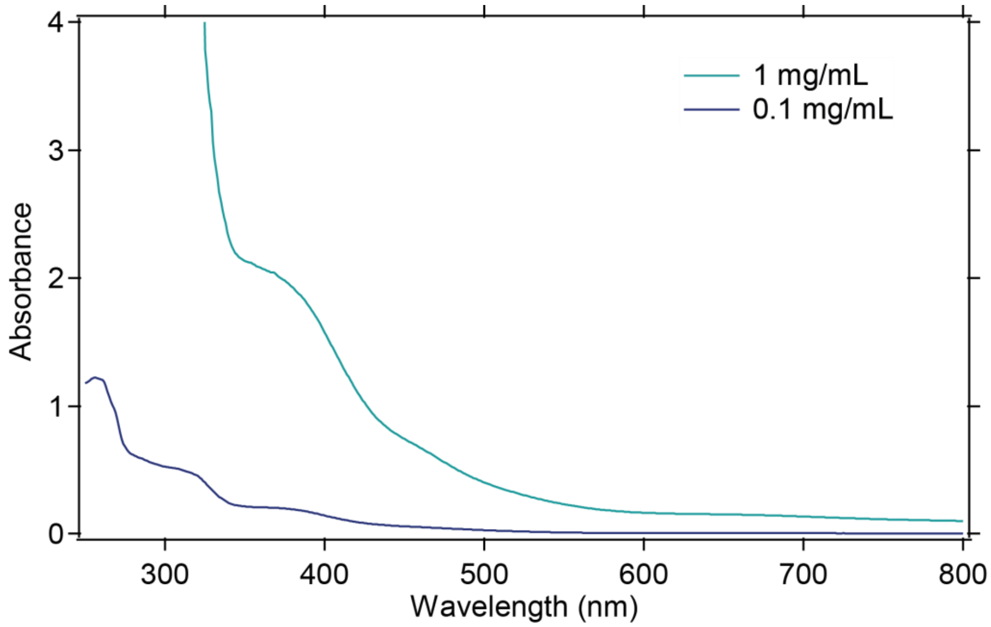

Figure S21 UV-vis spectra of **[Cu_2L](OTf)$_2$** and PhIO in DCM collected after 24 hours for a solution of 1 mg/mL **[Cu_2L](OTf)$_2$** (light blue) and a diluted solution of 0.1 mg/mL **[Cu_2L](OTf)$_2$** (light blue).

7.3 Chemical reduction of [Cu2L(μ-OH)]$^{3+}$

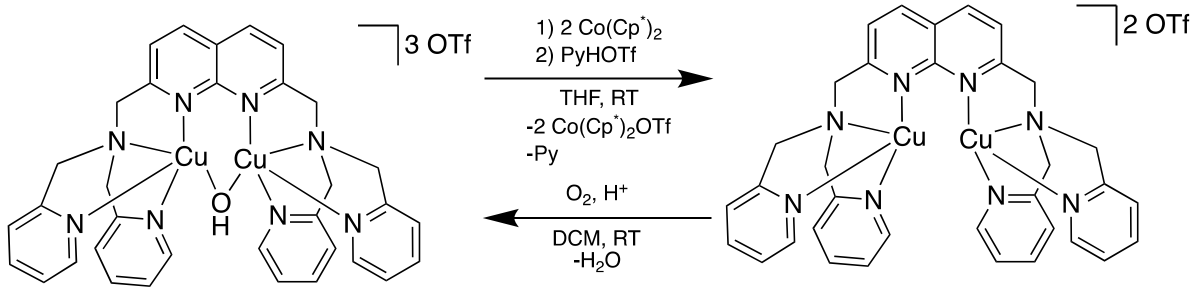

A solution of Co(Cp*)$_2$ (5.8 mg, 17.5 µmol) in THF (1.5 mL) was added dropwise to a stirring suspension of **[Cu_2L(µ-OH)](OTf)$_3$** (10.0 mg, 8.7 µmol) in THF (1.5 mL) changing the color to dark orange. After stirring at ambient temperature for 20 minutes, a solution of pyridinium triflate (2.0 mg, 8.7 µmol) in THF (1.5 mL) was added dropwise to the mixture, turning the mixture turbid and the color yellow/brown. After stirring for an additional 10 minutes at ambient temperature the mixture was concentrated under vacuum to a yellowish solid (14.5 mg).

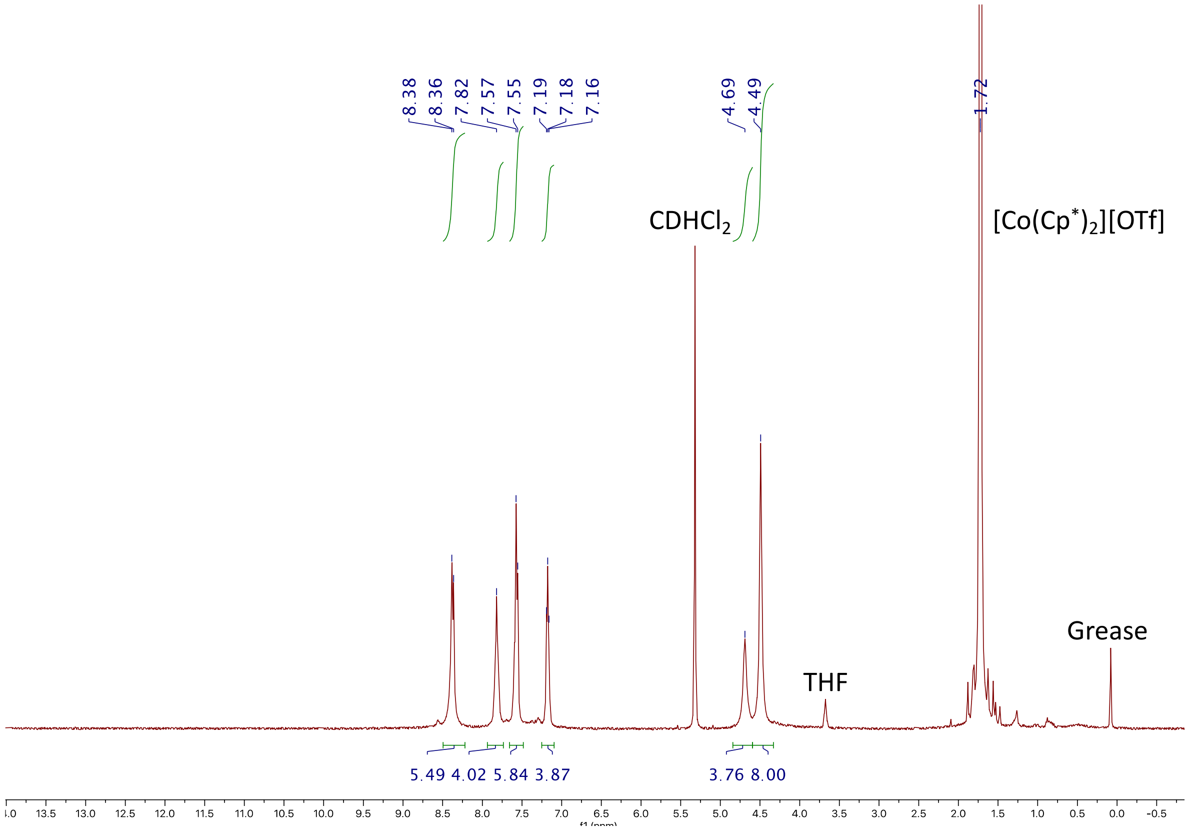

Figure S22 ^1H NMR spectrum after the addition of 2 electrons and one proton to **[Cu$_2$L(μ-OH)](OTf)$_3$** resulting in the formation of **[Cu$_2$L](OTf)$_2$**, at 25 °C.

7.4 Experiment with $[Cu_2L]^{2+}$and H_2O_2

H_2O_2 (from a 35% aqueous solution, 1.0 µL, 33 µmol) was diluted 1000-fold with D_2O and degassed. Subsequently, 0.3 mL of this solution was added slowly to a stirring suspension of **[Cu$_2$L](OTf)$_2$** (10.0 mg, 10.2 µmol) in degassed D_2O (0.6 mL), turning the color of the reaction mixture to a vivid green. The mixture was left to stir for 15 minutes and analyzed by NMR without further manipulations (See **Figure S23**) as any attempts towards aerobic work-up of the formed compound resulted in a change of color to dark orange over the course of 5-10 minutes. A UV-vis spectrum of the same reaction mixture was recorded in H_2O, indicating the formation of a Cu(II) species (See **Figure S24**).

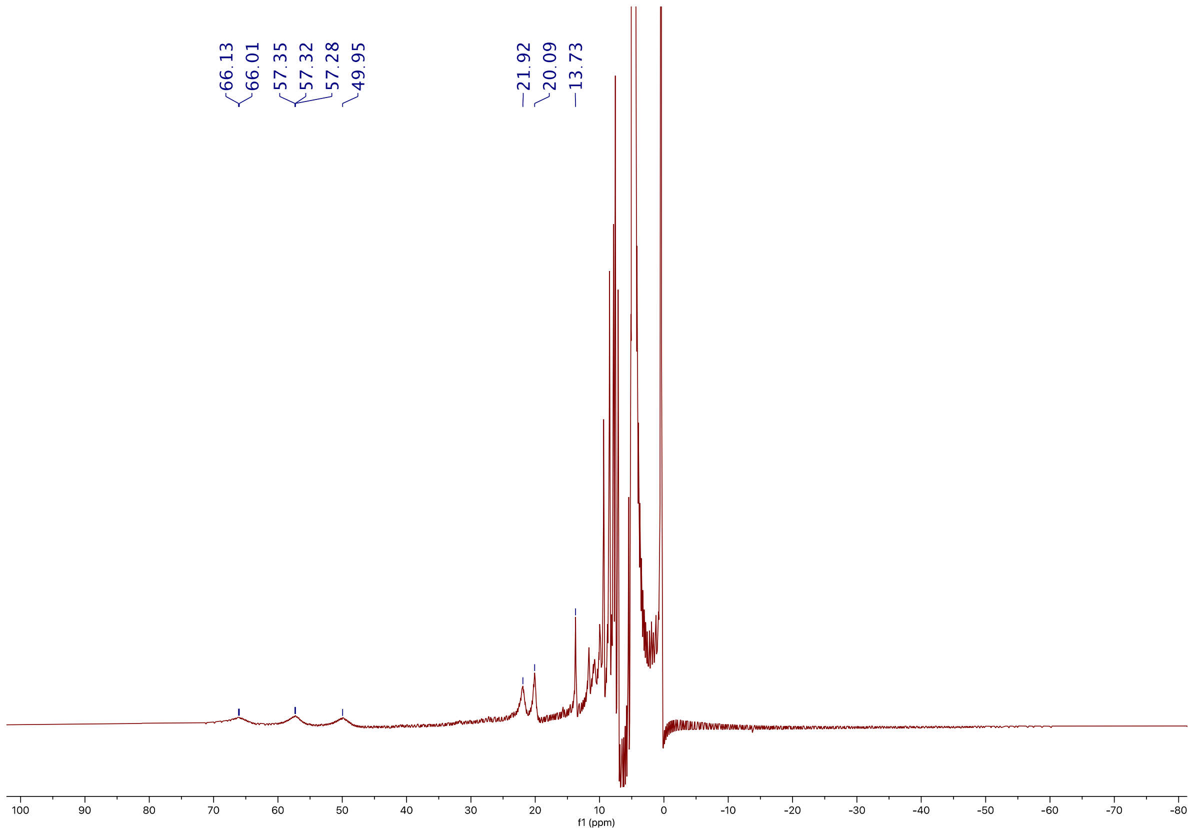

Figure S23 ^{1}H NMR (paramagnetic settings) spectrum of the reaction between **[Cu$_2$L](OTf)$_2$** and H_2O_2 in D_2O at 25 °C after 15 min.

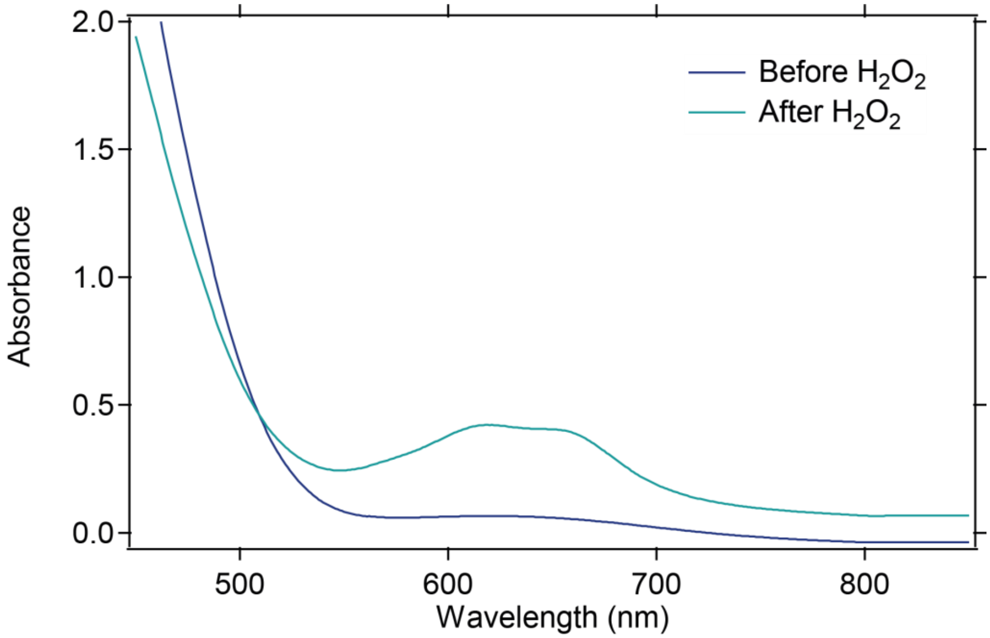

Figure S24 UV-vis spectra of 3 mM **$[Cu_2L](OTf)_2$** in degassed H_2O before (dark blue) and after addition of H_2O_2 (light blue).

7.5 Treatment of $[BPMANCu_2(\mu\text{-}OH)](OTf)_3$ with KOH

A KOH stock solution was prepared by dissolving KOH (5.0 mg, 87 µmol) in D_2O (1 mL). A portion of this stock solution (100 µL) was added to a stirring solution of $[BPMANCu_2(\mu\text{-}OH)](OTf)_3$ (10.0 mg, 8.7 µmol) in D_2O (0.6 mL), turning the solution green. The sample was left to stir for 2.5 hours at ambient conditions and was analyzed by NMR spectroscopy (See **Figure S25-26**). Attempts to remove the aqueous solvent resulted in a color change of the compound to orange.

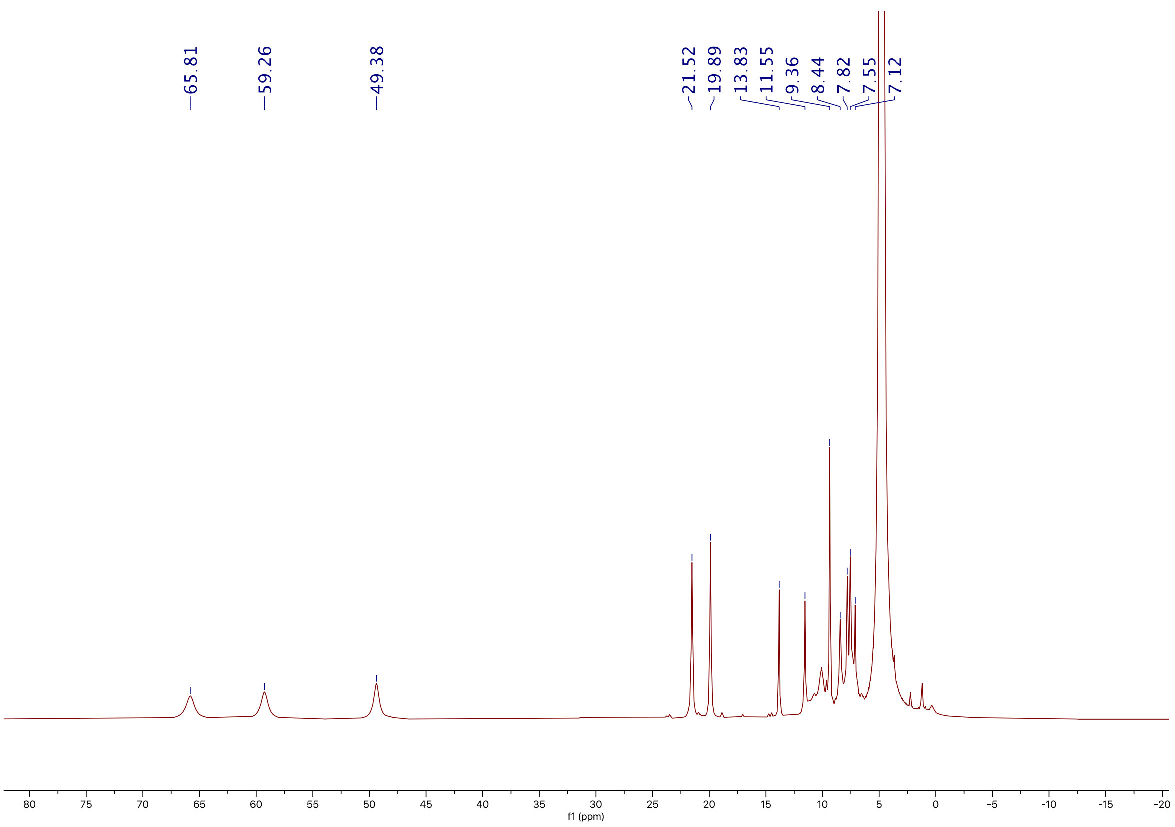

Figure S25 ^1H NMR (paramagnetic settings) spectrum of the reaction between **[Cu_2L(μ-OH)](OTf)$_3$** and KOH in D_2O at 25 °C after 15 min.

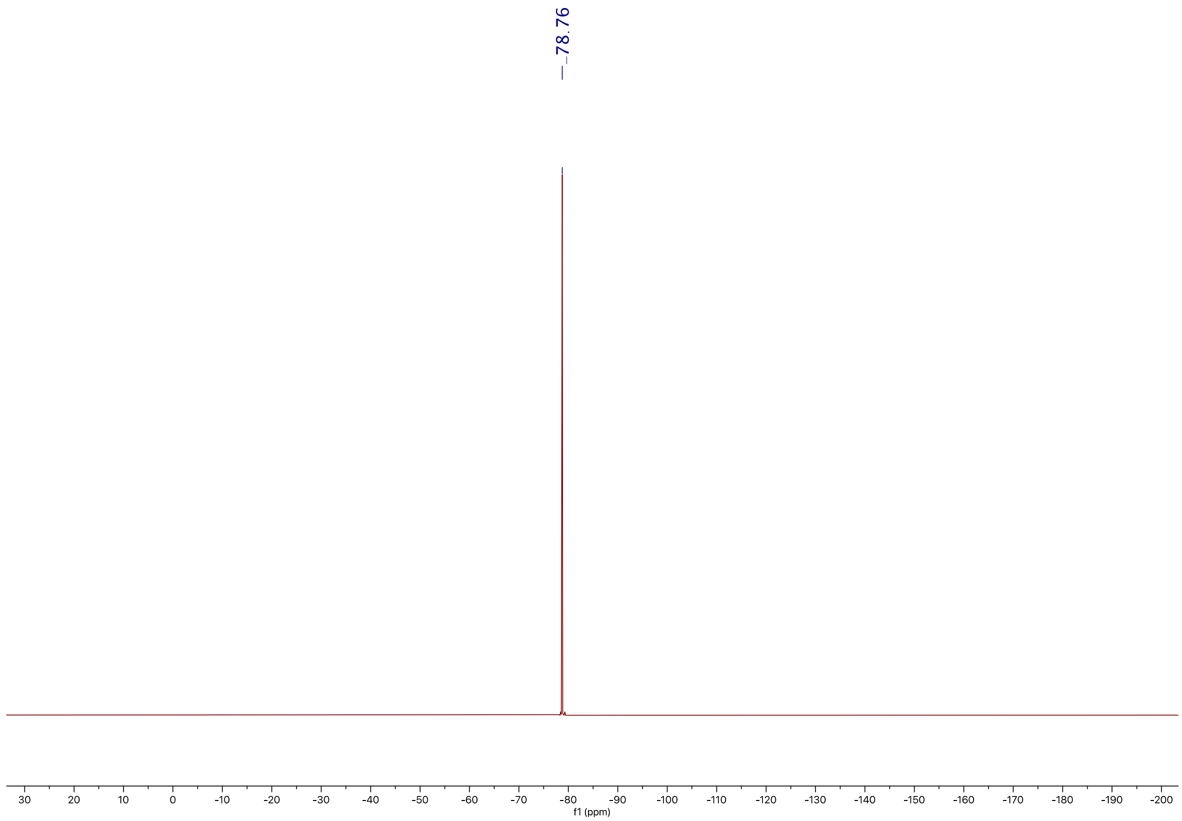

Figure S26 ^{19}F NMR spectrum of the reaction between **[Cu$_2$L(μ-OH)](OTf)$_3$** and KOH in D_2O at 25 °C after 15 min.

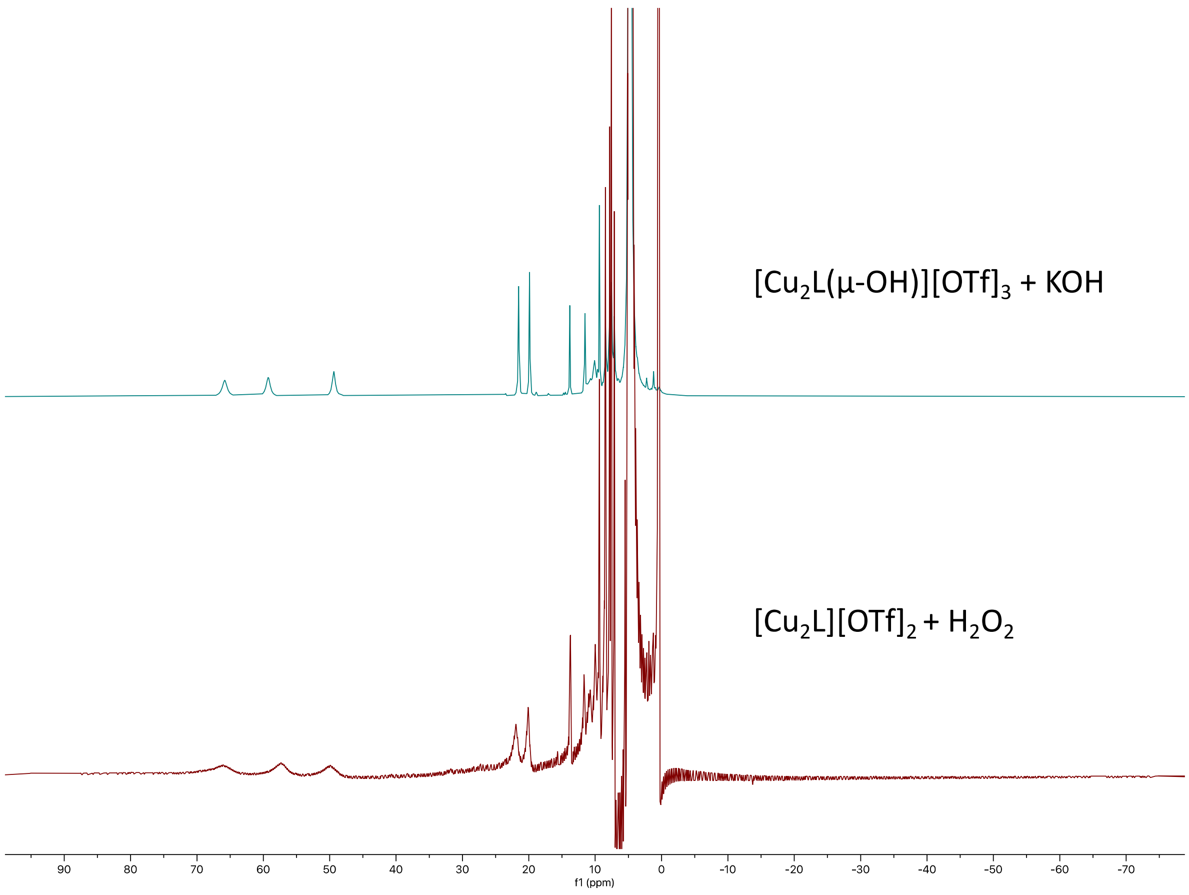

Figure S27 Stacked ^{1}H NMR spectra (paramagnetic settings) of the reaction between **[Cu$_2$L(μ-OH)](OTf)$_3$** and KOH and between **[Cu$_2$L](OTf)$_2$** and H_2O_2 in D_2O at 25 ℃.

8 Electrochemical measurements of [Cu_2L]$^{2+}$

Electrochemical measurements of **[Cu_2L]$^{2+}$** in acetonitrile were recorded in a similar way as described previously (**See section S1.2**). The measurements of **[Cu_2L]$^{2+}$** in aqueous solution were performed using a Ag/AgCl (0.3 M KCl) reference electrode instead of a RHE. Before every electrochemical experiment with **[Cu_2L]$^{2+}$**, the electrolyte solution was thoroughly sparged with argon and a flow of argon was maintained over the solution. The desired amount of **[Cu_2L](OTf)$_2$** was taken from a 0.18 mM acetonitrile solution stored under N_2 atmosphere with a syringe and transferred to the electrolyte solution.

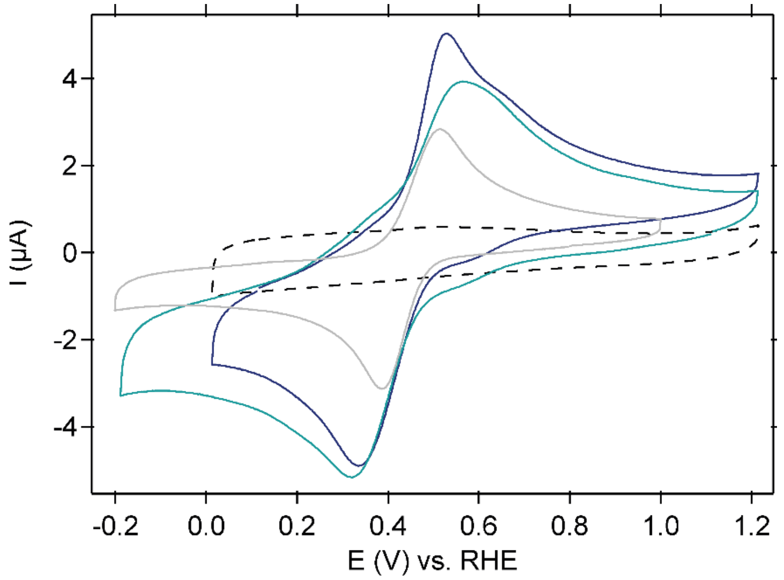

Figure S28 CVs of 0.3 mM **[Cu_2L]$^{2+}$** in PB recorded under Ar atmosphere (dark blue), and under Ar atmosphere after exposure of the solution to oxygen (light blue). CVs of a 0.15 mM **[Cu_2L(µ-OH)]$^{3+}$** solution (grey) and a bare GC electrode (dashed line) shown for reference. Conditions: 0.1 M PB pH 7, Ar atmosphere, 293 K, 100 mV/s scan rate.

When a solution of **[Cu_2L]$^{2+}$** in acetonitrile is exposed to air in the presence of $TEAPF_6$, a color change of the bulk solution from yellow to green is observed, suggesting the formation of a Cu(II) species (**Figure S29**). In the absence of any proton source, the color change is different, and a greyish-green solution forms. UV-vis measurements confirm that in the presence of air and protons **[Cu_2L]$^{2+}$** is converted to **[Cu_2L(µ-OH)]$^{3+}$**, while in the absence of protons a different species forms **(Figure S30**). Electrochemical measurements in acetonitrile confirm the formation of two distinct species (**Figure S31**). The almost complete disappearance of the Cu^{II}/Cu^{I} redox couple suggests that in the absence of protons **[Cu_2L]$^{2+}$** decomposes upon exposure to air.

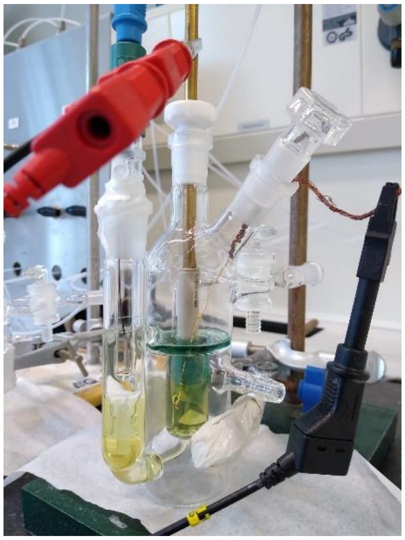

Figure S29 Observed color change in the electrochemical cell when a solution of **[Cu_2L]**$^{2+}$ (yellow) is exposed to air and the Cu(II) species forms (green) in the presence of 10 mM $TEAPF_6$.

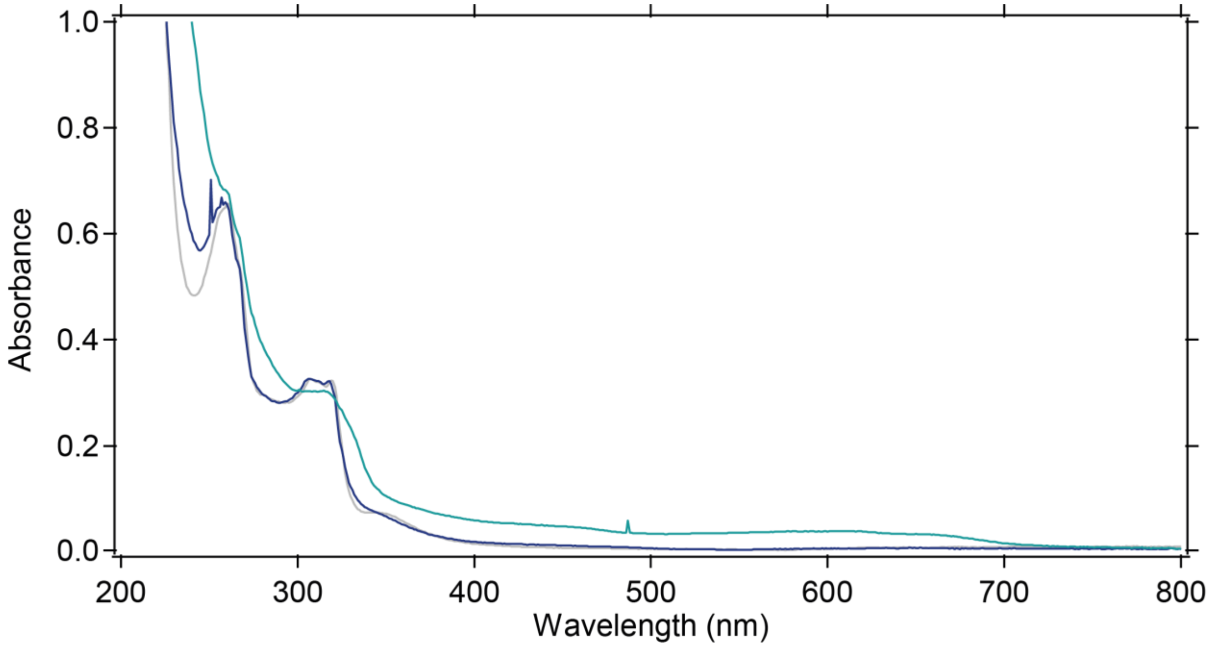

Figure S30 UV-vis spectrum of a 0.03 mM **[Cu_2L]**$^{2+}$ solution in MeCN with 0.1 M $TBAPF_6$ after exposing the solution to air in the presence of 20 mM $TEAPF_6$ (dark blue) and in the absence of $TEAPF_6$ (light blue). UV-vis of a 0.03 mM **[Cu_2L(μ-OH)]**$^{3+}$ solution in MeCN with 0.1 M $TBAPF_6$ (grey) shown as reference.

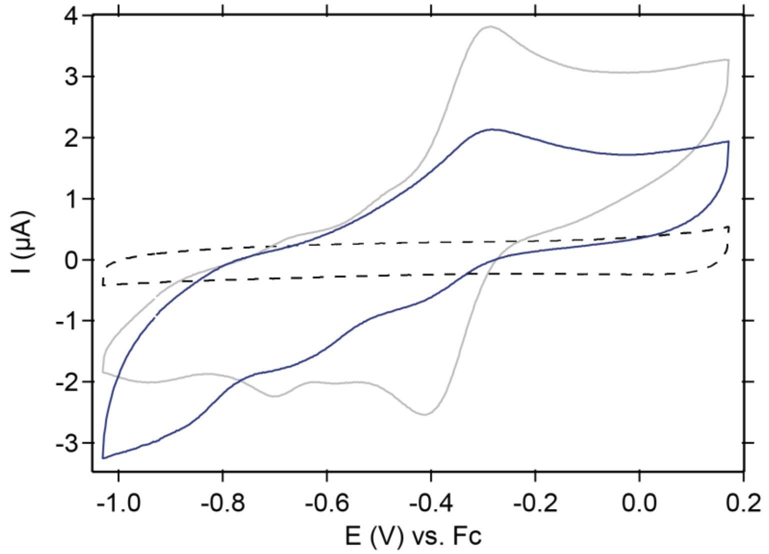

Figure S31 CVs of a 0.3 mM **[$Cu_2L]^{2+}$** solution in MeCN under Ar after exposure to air in the absence of any proton source (dark blue). CV of 0.15 mM **[Cu_2L(µ-OH)]$^{3+}$** in MeCN (grey) and the bare GC electrode (dashed line) for comparison. Conditions: 0.1 M TBAPF6 in MeCN, Ar atmosphere, 293 K, 100 mV/s scan rate.

9. K_{obs} for ORR and HPRR in different buffers

Values for the k_{obs} were determined from the background corrected currents of the ORR and HPRR measurements under non-substrate limited conditions. The measurements from the concentration dependence studies (**Figure 7,** main text) were used to determine the values in PB of pH 7. To determine the k_{obs} values in acetate buffer of pH 4.85, measurements with 3 µM catalyst concentrations were recorded. All background-corrected CV measurements in both buffers are shown in **Figure S32**. The diffusion coefficient of **[Cu$_2$L(µ-OH)]$^{3+}$** in acetate buffer was determined in the same manner as described in section SI 5, and is 9.2 × 10^{-7} cm^2/s for the reduced species. The final values of k_{obs} in **Table S2** were calculated as the average value determined for all catalytic waves in **Figure S32**.

Table S2 Overview of the average calculated values of k_{obs} for catalysis of the ORR and HPRR by **[Cu$_2$L(µ-OH)]$^{3+}$** in phosphate buffer and acetate buffer.

	Phosphate buffer pH 7.0	*Acetate buffer pH 4.85*
Reaction	**k_{obs} (×10^3 s^{-1})**	**k_{obs} (×10^3 s^{-1})**
ORR 2e$^-$	14.7 ± 4.2	48.4 ± 8
ORR 4e$^-$	3.7 ± 1.0	12.1 ± 2
HPRR	4.8 ± 1.4	4.6 ± 0.03

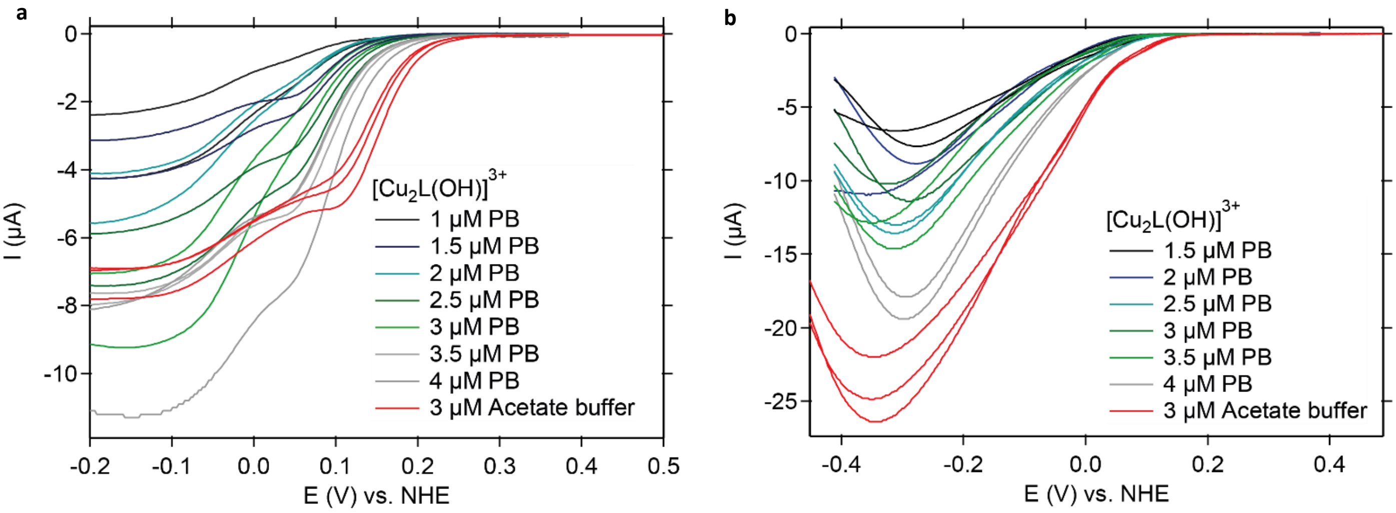

Figure S32 Background-corrected CVs of different concentrations of **[Cu$_2$L(µ-OH)]$^{3+}$** recorded in PB (grey, blue, green traces) and acetate buffer (red traces) **a)** in the presence of 1.1 mM H_2O_2 or **b)** under O_2 atmosphere. Conditions: 0.1 M PB pH 7 or 0.1 M acetate buffer pH 4.85, Ar or O_2 atmosphere, 293 K, 100 mV/s scan rate.

10. HPRR measurements in D_2O

Before every electrochemical experiment in D_2O, all glassware was dried in an oven at 140 °C overnight. The GC electrode was polished as described before and sonicated in D_2O. The counter electrode was flame annealed and rinsed with D_2O before the experiments. The electrolyte solution was prepared from non-deuterated phosphate salts in D_2O. The apparent pH of the prepared solutions was determine using a pH meter calibrated with H_2O solutions and converted to obtain pH values of 6.99 and 7.02.[5] To prepare the 1.1 mM H_2O_2 solutions, a small volume of H_2O_2 was added from a 10 M stock solution to ensure that the proton content was minimal.

Figure S33 shows the CVs of **$[Cu_2L(\mu\text{-}OH)]^{3+}$** measured in H_2O and D_2O under non-catalytic and catalytic conditions. The small shift in peak potential of the **$[Cu_2L(\mu\text{-}OH)]^{3+}$** redox couple either originates from a small discrepancy in pH between the two measurements, or the relatively easier loss of the bridging hydroxyl ligand from the copper center in its deuterated form. The kinetic isotope effect (KIE) of the HPRR was determined from measurements of 0.15 mM **$[Cu_2L(\mu\text{-}OH)]^{3+}$** in 20 mM H_2O_2, as under these conditions the substrate is not limited and the most reliable KIE can be determined. **Equation 3** was used to determine a KIE from averaged values of five catalytic measurements in D_2O and two in H_2O electrolyte. Because of the absence of a clear plateau current, the background-correct values at 0.2 V vs. RHE were used.

$$KIE = \frac{k_{obs,H}}{k_{obs,D}} \propto \quad \textbf{(3)}$$

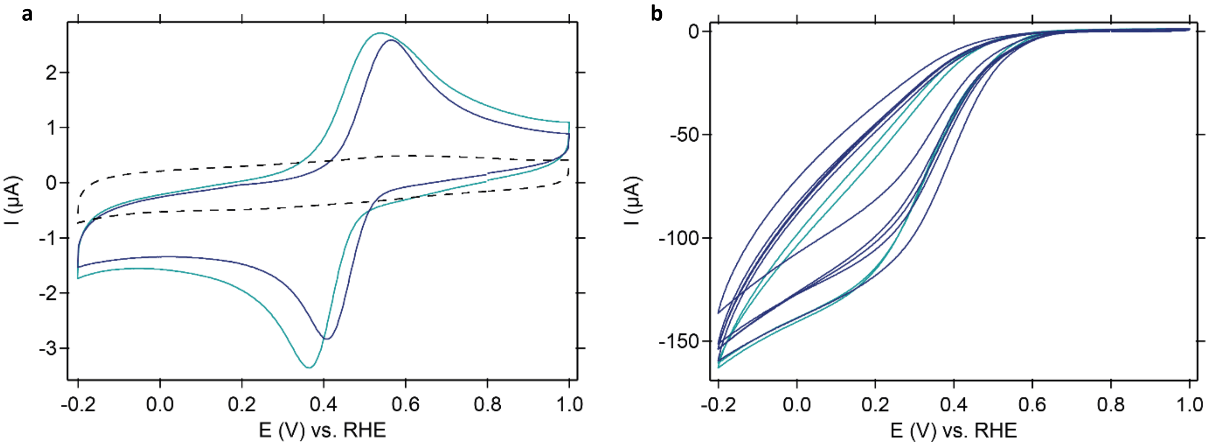

Figure S33 CVs of **$[Cu_2L(\mu\text{-}OH)]^{3+}$** recorded in H_2O (light blue traces) and D_2O (dark blue traces) under **a)** Ar atmosphere and **b)** in the presence of 20 mM H_2O_2. Conditions: 0.15 mM **$[Cu_2L(\mu\text{-}OH)]^{3+}$**, 0.1 M PB pH 7, Ar atmosphere, 293 K, 100 mV/s scan rate.

11. Radical trapping experiments

To investigate the catalytic HPRR mechanism of **$[Cu_2L(\mu\text{-}OH)]^{3+}$**, hydroxyl radical trapping experiments were performed using 5,5-dimethyl-1-pyrroline N-oxide (DMPO).[6] Treating a MeCN solution of **$[Cu_2L]^{2+}$** and DMPO with H_2O_2 in the absence of oxygen led to a change of color from dark yellow to green. The characteristic resonance of the DMPO-OH radical was not detected in the EPR spectrum, which is indicative of non-Fenton type reactivity taking place (See **Figure S34a**). As a positive control, an EPR spectrum of a DMPO solution with H_2O_2 and $FeSO_4$ was recorded, inducing a Fenton reaction and resulting in the characteristic EPR signal of the DMPO-OH radical (**See Figure S34b**).

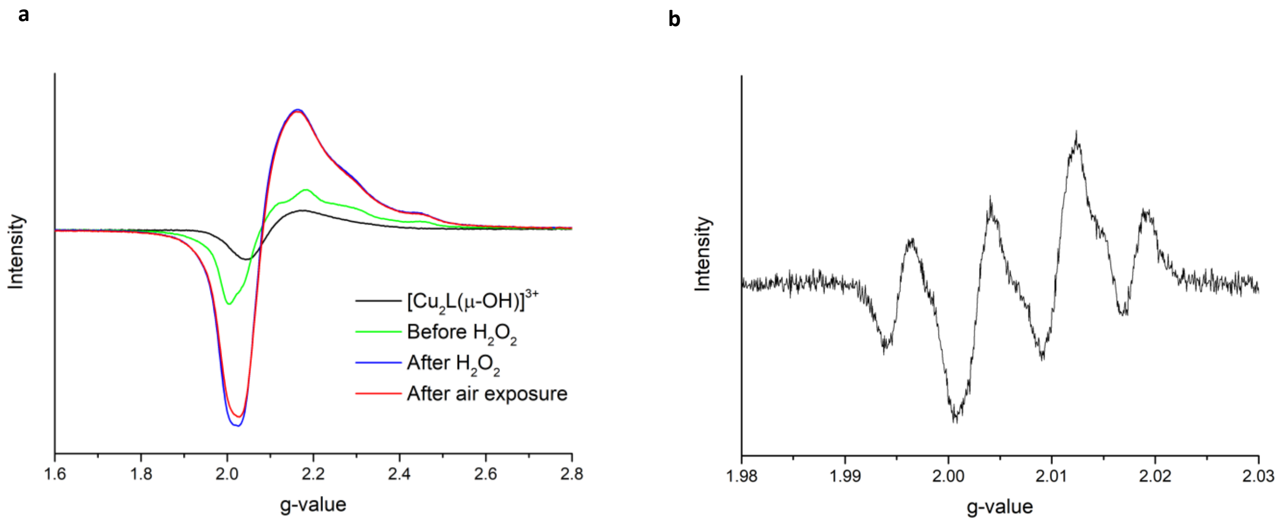

Figure S34 a) Left: EPR spectra of a solution of 7.3 mM **$[Cu_2L]^{2+}$** with 1.2 equiv. DMPO before (green) and after (blue) addition of 1.7 equiv. H_2O_2, and after exposure of the sample to air (red). EPR spectrum of a 1 mM **$[Cu_2L(\mu\text{-}OH)]^{3+}$** (black) shown for reference. All recorded in MeCN at 130 K **b)** Right: EPR spectrum of DMPO with H_2O_2 and Fe_2SO_4 recorded in MeCN at RT.

12. RRDE measurements

12.1 RDE CVs and Koutecky-Levich analysis

Catalytic RDE CVs of **[Cu$_2$L(μ-OH)]$^{3+}$** were recorded at varying rotation rates between 400 RPM and 2800 RPM (**Figure S35a**). The corresponding Koutecky-Levich plot (**Figure S35b**) shows linearity over the complete range, suggesting that the number of electrons transferred at -0.4 V vs. RHE in the ORR is independent of the rotation rate.

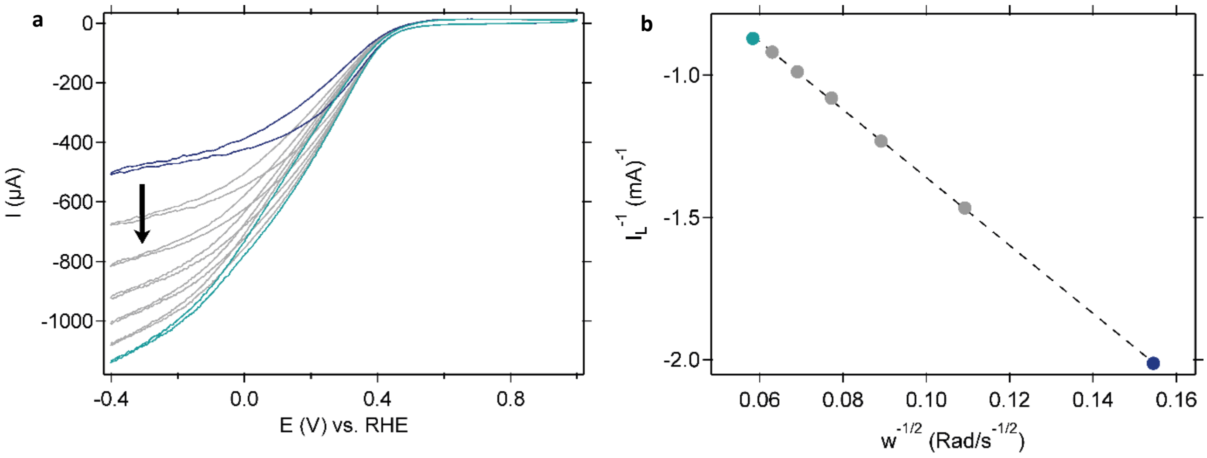

Figure S35 a) RDE CVs of 0.15 mM **[Cu$_2$L(μ-OH)]$^{3+}$** under oxygen atmosphere with varying rotation rates between 400 RPM (dark blue trace) and 2800 RPM (light blue trace). **b)** Corresponding Koutecky-Levich plot derived from the catalytic currents at -0.4 V vs. RHE. Conditions: 0.1 M PB pH 7, O_2 atmosphere, 293 K, 50 mV/s scan rate, 1600 RPM.

12.2 H_2O_2 selectivity in RRDE

To calculate the ORR product selectivity in RRDE experiments, any contribution from oxidation of the formed **[Cu$_2$L]$^{2+}$** species to the observed ring current should be measured. By fixing the Pt ring at 0.8 V vs. RHE, a potential below the oxidation potential of H_2O_2, but above the oxidation potential of **[Cu$_2$L]$^{2+}$**, it was observed that oxidation of the catalyst hardly contributes to the ring current (**Figure S36**). This denotes that the largest part of the ring current corresponds to the detection of any H_2O_2 produced in the ORR.

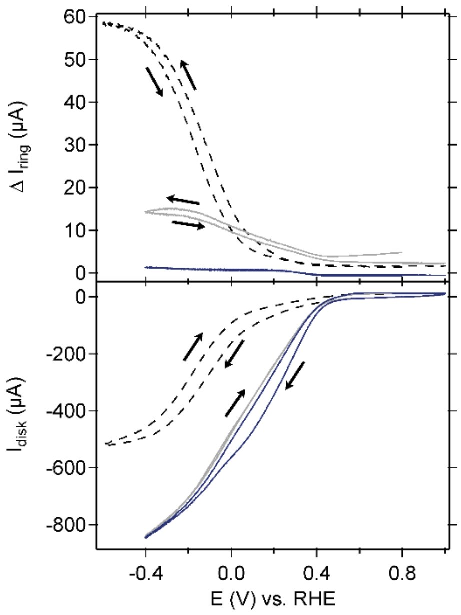

Figure S36 RRDE CVs of 0.15 mM **[Cu_2L(μ-OH)]$^{3+}$** solution under oxygen atmosphere with the Pt ring fixed at 1.2 V vs. RHE (grey) and at 0.8 V vs. RHE (dark blue). CV of the bare GC electrode under O_2 atmosphere shown for comparison (dashed lines, Pt ring at 1.2 V vs. RHE) Conditions: 0.1 M PB pH 7, O_2 atmosphere, 293 K, 50 mV/s scan rate, 1600 RPM, Pt ring at 0.8 or 1.2 V vs. RHE.

It is important to determine the collection efficiency of the Pt ring prior to every RRDE experiment in order to precisely quantify the H_2O_2 that is produced in the ORR. The collection efficiency (*N*) was determined from **Equation 5**, using the current collected at the ring (i_{ring}) and current collected at the disk (i_{disk}) obtained in a CA measurement of the ORR activity of a blank GC electrode at -0.1 V vs. RHE during 5 minutes, as shown in **Figure S37**. Since GC is a 100% selective catalyst to H_2O_2, the collection efficiency of the ring can be calculated by dividing the ring current by the disk current. The currents recorded at -0.1 V vs. RHE were corrected for the average ring current recorded for the GC double layer (at 0.8 V vs. RHE) during the 60 seconds prior to the experiment. The final collection efficiency was determined as the average collection efficiency of the RRDE in the first 30-60 seconds of the experiment, and typically a value between 9 and 15 % was found.

$$N = \frac{i_{ring}}{i_{disk}} \quad \textbf{(5)}$$

The selectivity of **[$Cu_2L(\mu\text{-}OH)]^{3+}$** to H_2O_2 during catalysis of the ORR was calculated from **Equation 6**. To increase the accuracy of the selectivity in the LSV experiment, the catalytic currents were corrected for the average currents measured between 0.8 and 1.0 V vs. RHE. In the same manner, the H_2O_2 selectivity in CA measurements (**Figure S38**) was determined by correcting for the average currents recorded at the ring with the GC disk set to 0.8 V vs. RHE during the 60 seconds prior to the experiment. The H_2O_2 selectivity in **Figure 8** (main text) was determined as the average selectivity of the first 60-90 seconds of the measurement the CA measurements in **Figure S38** using **Equation 6**.

$$\%H_2O_2 = \frac{2\left(\frac{i_{ring}}{N}\right)}{i_{disk}+\left(\frac{i_{ring}}{N}\right)} \times 100\% \quad \textbf{(6)}$$

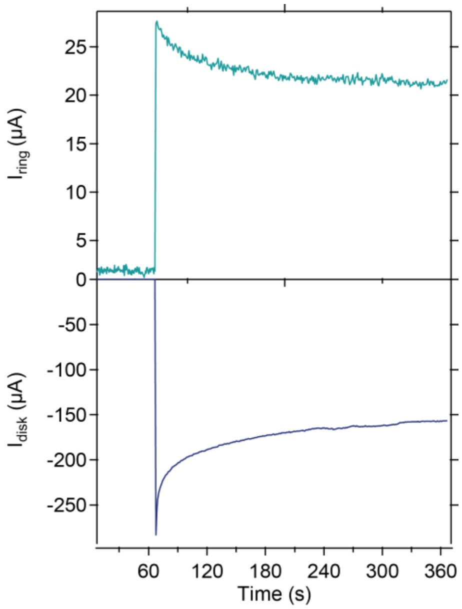

Figure S37 Typical chronoamperogram of a bare GC disk fixed at 0.8 V (0-60 s) and at -0.1 V vs. RHE (60–360 s) (dark blue) and the corresponding current recorded at the Pt ring (light blue) to determine the collection efficiency of the RRDE setup. Conditions: 0.1 M PB pH 7, O_2 atmosphere, 293 K, 1600 RPM, Pt ring at 1.2 V vs. RHE.

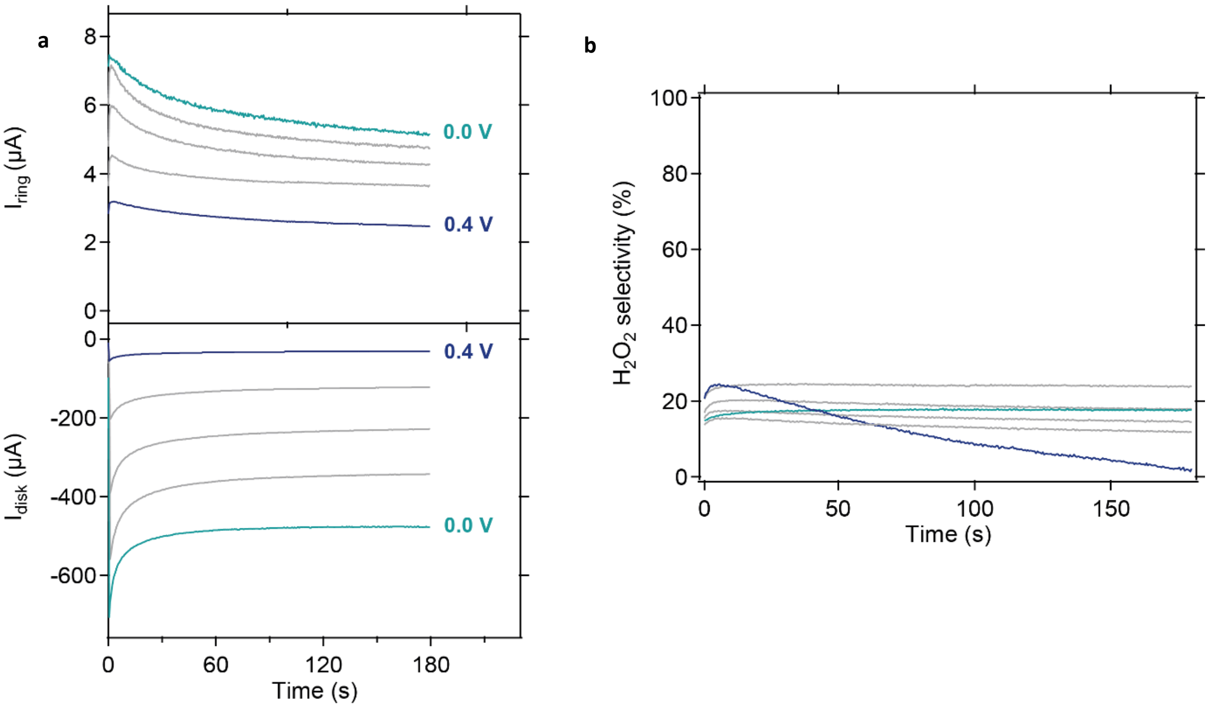

Figure S38 a) RRDE CA data of the ORR catalysed by **[Cu$_2$L(μ-OH)]$^{3+}$** at potentials of 0.0 V (light blue), 0.1 V, 0.2 V, 0.3 V (grey), and 0.4 V (dark blue) vs. RHE. **b)** H_2O_2 selectivity determined from the CA data shown in a). Conditions: 0.15 mM **[Cu$_2$L(μ-OH)]$^{3+}$**, 0.1 M PB pH 7, O_2 atmosphere, 293 K, 1600 RPM, Pt ring at 1.2 V vs. RHE.

12.3 Formation of deposits in RRDE experiments

Deposit checks for the RRDE measurements were carried out in the same manner as for stationary CV experiments. In the first experiment, three RRDE CV cycles under O_2 were recorded in a 0.15 mM **[Cu$_2$L(μ-OH)]$^{3+}$** solution, after which the RRDE electrode was rinsed with Mili-Q. Thereafter, an RRDE CV of the rinsed electrode was recorded in a blank buffer solution under O_2 atmosphere. The rinsed electrode shows a higher ORR activity than the bare GC electrode, but the activity is lower than of the homogeneous catalyst in solution (**Figure S39a**). Next the ORR selectivity to H_2O_2 was determined for the deposit. This shows that the deposit has a similar selectivity as the homogeneous **[Cu$_2$L(μ-OH)]$^{3+}$** species (**Figure S39b**).

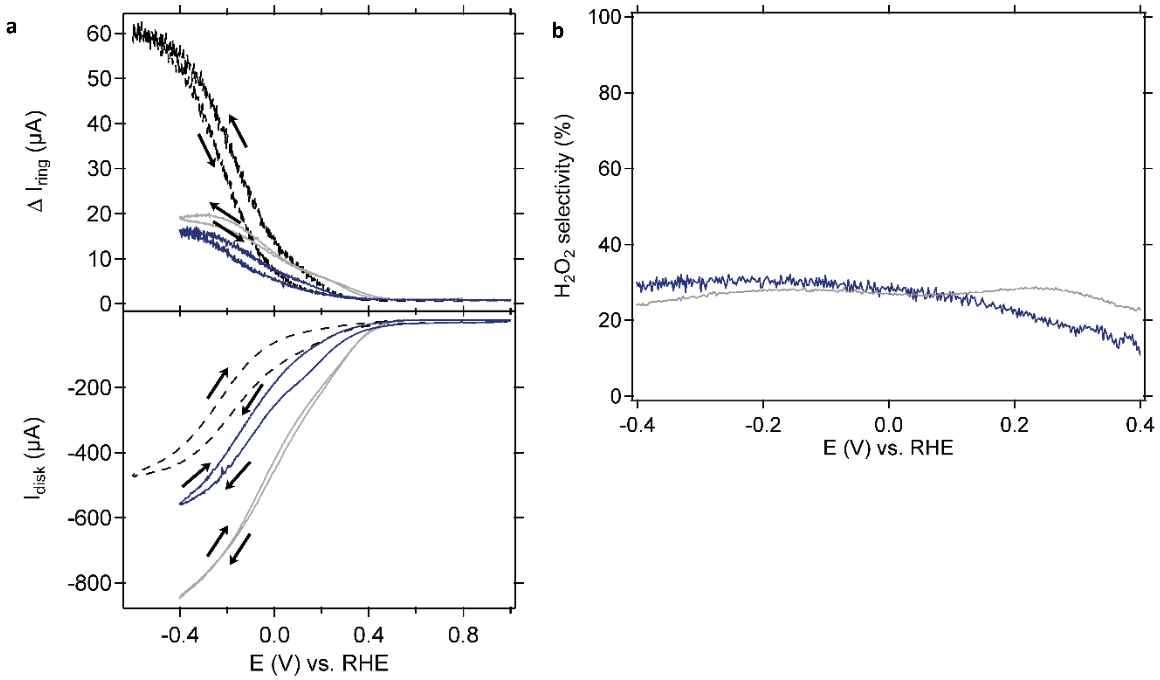

Figure S39 a) RRDE CV of the ORR activity of a 0.15 mM **[Cu$_2$L(μ-OH)]$^{3+}$** solution (grey) compared to the deposits formed after three ORR scans (blue). Bare GC electrode in O_2 atmosphere shown for comparison (black dashed trace). **b)** Corresponding H_2O_2 selectivities of 0.15 mM **[Cu$_2$L(μ-OH)]$^{3+}$** (dark blue) and that of the deposits that form during ORR (grey traces) determined from the RRDE CVs in a). Conditions: 0.1 M PB pH 7, O_2 atmosphere, 293 K, 50 mV/s scan rate, 1600 RPM, Pt ring at 1.2 V vs. RHE.

13. Stability of H_2O_2 solutions in the presence of [Cu$_2$L(μ-OH)]$^{3+}$

The disproportionation of H_2O_2 in PB in the presence of **[Cu$_2$L(μ-OH)]$^{3+}$** was monitored over time and compared to a control solution with H_2O_2 in the absence of catalyst (**Figure S40a**). This indicates that **[Cu$_2$L(μ-OH)]$^{3+}$** enhances the decomposition of H_2O_2, but at such low rates that over the time course of an RRDE experiment this will not affect the ORR selectivity. UV-vis spectra of the solution with H_2O_2 and **[Cu$_2$L(μ-OH)]$^{3+}$** were recorded simultaneously, showing that **[Cu$_2$L(μ-OH)]$^{3+}$** itself is stable in the presence of H_2O_2 (**Figure S40b**).

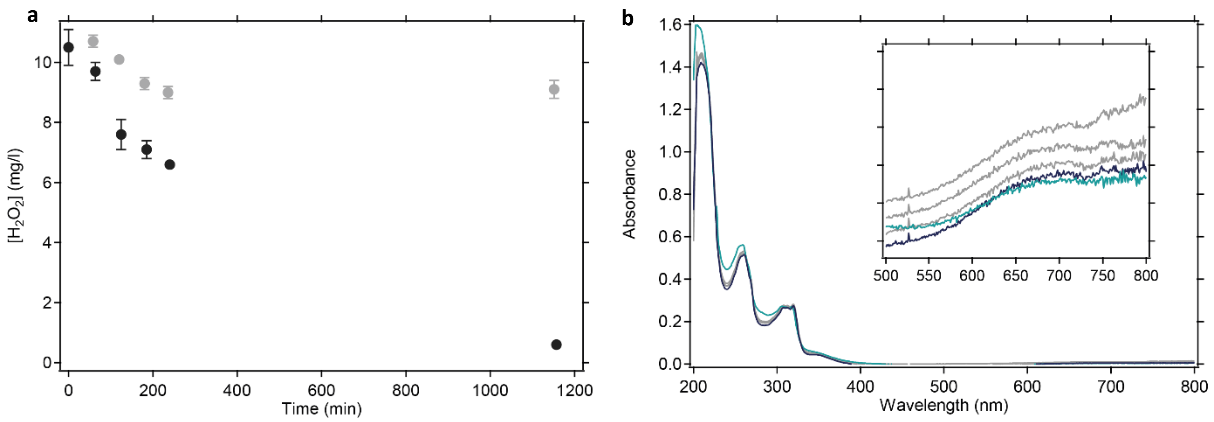

Figure S40 a) The concentration of H_2O_2 in a blank solution of 0.1 M PB (grey markers) and in a solution of 0.1 M PB with 0.3 mM **[Cu₂L(μ-OH)]³⁺** (black markers). The H_2O_2 added in both solutions amounted to 0.3 mM. **b)** UV-VIS measurements of the solution with **[Cu₂L(μ-OH)]³⁺** and H_2O_2 (10x diluted), followed over time (dark blue t = 30 min, light blue t= 1150 min). The inset shows the d-d transition.

14. Computational methods

14.1 General remarks

Calculations were performed using the Gaussian 16 rev. C01 software.[7] The Becke 1988 exchange functional (B3LYP) was used.[8-11] For the geometry optimizations the redefinition of Ahlrichs split valence basis set (def2SVP) was used on all atoms.[12, 13] Starting geometries for the optimizations were obtained from the coordinates of the crystal structures if possible, or by modification of the optimized geometry of the most similar complex. For the single point calculation on the optimized structures, the redefinition of Ahlrichs triple-zeta split valence basis set (def2-TZVP) was used on all atoms.[12] A SMD continuum solvation model for water was used for the best approximation of the experimental conditions.[14] Additionally, Grimme's DFT-D3 scheme for atom-pairwise dispersion correction with the Becke-Johnson damping(GD3BJ) was used for all atoms in every calculation.[15, 16]

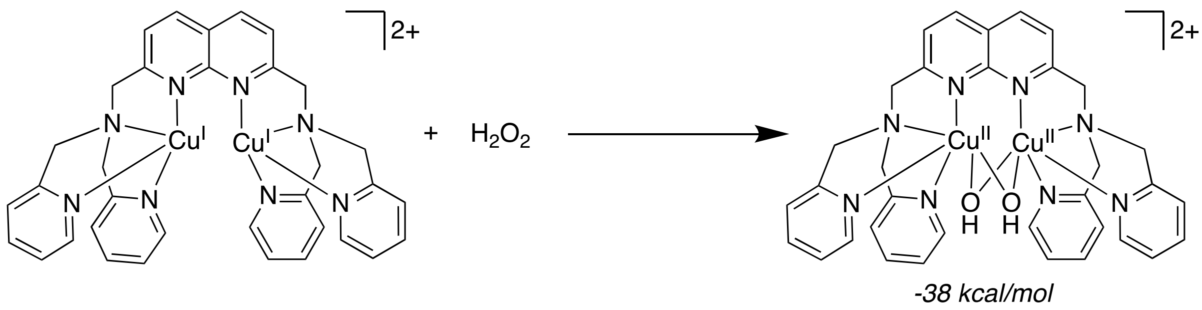

A comparison of the energies of the optimized geometries of **$[Cu_2L(\mu\text{-}OH)_2]^{2+}$** with that of **$[Cu_2L]^{2+}$** and H_2O_2, show that the former is lower in energy by approx. 38 kcal/mol. These calculations support our hypothesis that it is energetically feasible that H_2O_2 is activated over the dicopper(I) core. Moreover, our calculations show that the difference in energy between the triplet and BS Singlet state of the **$[Cu_2L(\mu\text{-}OH)_2]^{2+}$** is small and lies within the error of the computations.

Example of an input file for the geometry optimization:

#opt freq B3LYP/def2SVP empiricaldispersion=gd3bj SCRF=(SMD,Solvent=Water)

“Title”

2 3

“Coordinates”

Example of an input file for the singe point calculation:

#SP B3LYP/def2TZVP empiricaldispersion=gd3bj SCRF=(SMD,Solvent=Water)

“Title”

2 3

“Coordinates from geometry optimization”

14.2 Optimized XYZ coordinates and computed energies

$[Cu_2L]^{2+}$:

Electronic energy: -5034.79967401 Hartrees

Gibbs Thermal Correction: 0.542046 Hartrees

Total: -5034.257628 Hartrees

Cu 1.30617500 -0.12046600 -0.04742700

Cu -1.06465700 0.72238800 -0.15824700

N 0.63720100 -1.78246300 -1.10901100

N -1.42594100 -0.82495700 -1.53416900

N 2.98510100 -1.59740200 0.40071500

N 2.88378300 0.88917100 -0.93420500

N 1.22393000 -0.11791400 2.02615700

N -3.15669400 1.32063100 -0.89513900

N -0.76112000 2.69886300 -0.21089300

N -2.24052700 -0.38370000 1.22224100

C 1.46813500 -2.82020300 -1.12148700

C 1.06724600 -4.09648100 -1.58523300

H 1.78372400 -4.91907200 -1.56522300

C -0.21806400 -4.27713300 -2.04677400

H -0.56105300 -5.25057800 -2.40388800

C -2.46169900 -3.24329600 -2.48771500

H -2.86248100 -4.19233300 -2.85019700

C -3.24157700 -2.11058600 -2.43530500

H -4.28442600 -2.12558600 -2.75571600

C -2.68519400 -0.90207800 -1.94568100

C -0.63522800 -1.92949400 -1.56364000

C -1.11373700 -3.17866300 -2.04988300

C 2.89591900 -2.57470300 -0.67230800

H 3.37392200 -3.53787900 -0.41618900

H 3.44126800 -2.17964200 -1.54579700

C 4.16387600 -0.74381700 0.37095400

H 5.10342600 -1.31536300 0.25702700

H 4.22932500 -0.22677800 1.34269500

C 4.08698300 0.32457800 -0.70304200

C 5.23013200 0.74677700 -1.38562200

H 6.18665800 0.26056200 -1.18486300

C 5.12929500 1.78443400 -2.31341700

H 6.01232600 2.12807800 -2.85652000

C 3.88129800 2.36796200 -2.53910800

H 3.75148600 3.17961400 -3.25672200

C 2.78512200 1.88385400 -1.82918600

H 1.78817000 2.30361300 -1.98299800

C 2.62317700 -2.10788600 1.71364700

H 3.47577300 -2.57638000 2.23969800

H 1.87287000 -2.90331200 1.56933300

C 1.98838400 -1.06230200 2.60906000

C 2.12846400 -1.12889200 3.99793300

H 2.76596100 -1.89794200 4.43795400

C 1.44357400 -0.21530600 4.79905500

H 1.53859700 -0.25310400 5.88637400

C 0.63763900 0.74630700 4.18591100

H 0.07869600 1.48014700 4.76872800

C 0.55937500 0.75795600 2.79653500

H -0.06475000 1.48462600 2.27474800

C -3.52073000 0.36766700 -1.93679400

H -3.37277500 0.84186000 -2.92062400

H -4.58839500 0.09049400 -1.88728600

C -2.97310300 2.70654400 -1.30470700

H -3.89392100 3.30836300 -1.19669800

H -2.72329800 2.71239500 -2.37757000

C -1.83463800 3.42097500 -0.59432100

C -1.86530100 4.80848300 -0.42985300

H -2.75401200 5.36457700 -0.73433700

C -0.75899700 5.46098400 0.11372300

H -0.76721500 6.54477900 0.24771200

C 0.35593700 4.70499200 0.48377300

H 1.24740700 5.16994100 0.90746000

C 0.30909800 3.32607000 0.30903000

H 1.15160900 2.69108900 0.58910000

C -3.90607700 1.15502800 0.35036500

H -4.99791000 1.20399100 0.18324900

H -3.63662500 1.98849300 1.01906700

C -3.55434900 -0.14039300 1.04853200

C -4.52649300 -1.04252400 1.48220300

H -5.58238500 -0.81878600 1.32053900

C -4.12523100 -2.22676800 2.10589400

H -4.86842000 -2.95035200 2.44772400

C -2.76190200 -2.47419200 2.26989400

H -2.40041600 -3.39046600 2.73942400

C -1.85306400 -1.52228100 1.80867500

H -0.77690700 -1.68006200 1.90182100

H_2O_2:

Electronic energy: -151.631695229 Hartrees

Gibbs Thermal Correction: 0.003386 Hartrees

Total: -151.6283092 Hartrees

O -0.71087700 -0.14270800 -0.00000100

O 0.71087700 0.14270800 -0.00000100

H -1.06185800 0.76430400 0.00000700

H 1.06185800 -0.76430400 0.00000700

H_2O_2+**$[Cu_2L]^{2+}$:** -5185.885937 Hartrees

$[Cu_2L(\mu\text{-}OH)_2]^{2+}$ (Triplet)**:**

Electronic energy: -5186.51928307 Hartrees

Gibbs Thermal Correction: 0.571491 Hartrees

Total: -5185.947792 Hartrees

Cu -1.29147500 -0.43380000 0.09919600

Cu 1.58481100 -0.06844400 0.24030100

N -0.98722600 1.79135900 1.66901000

N 0.96139000 2.35279400 0.58540600

N -2.96519700 -0.50385300 1.52498700

N -2.74297600 0.47936600 -1.02621500

N -1.85874600 -2.61154000 0.13368800

N 3.13578400 1.03900000 -0.84468300

N 2.44498800 -1.55024500 -1.44556400

N 3.08462900 -0.11794100 1.62442400

C -2.30267600 1.85266800 1.78085900

C -3.07312800 2.86667600 1.15314500

H -4.14933800 2.90856100 1.32192100

C -2.44961300 3.73190600 0.27945900

H -3.01608500 4.49214300 -0.26244400

C -0.34072800 4.27403700 -0.97999400

H -0.85126800 5.02642700 -1.58539700

C 0.97902700 3.94565700 -1.21249800

H 1.55007700 4.42180900 -2.01082000

C 1.59422500 2.95462500 -0.40397200

C -0.35409800 2.59968900 0.78338300

C -1.06054000 3.58171400 0.02678700

C -2.92870500 0.65722700 2.46574500

H -2.29954500 0.37615500 3.31859800

H -3.94249500 0.87959200 2.83206300

C -4.16875200 -0.48030800 0.66631600

H -5.06216700 -0.19161600 1.24204600

H -4.34568900 -1.49892900 0.28915700

C -3.98744500 0.42370800 -0.52085800

C -5.03296700 1.15001000 -1.09044900

H -6.03231300 1.08914100 -0.65735200

C -4.76605300 1.96321500 -2.19187800

H -5.56545400 2.55026100 -2.64877600

C -3.46235300 2.03319900 -2.68813700

H -3.20770400 2.67247400 -3.53437800

C -2.47298300 1.27295700 -2.07139800

H -1.43150500 1.29212800 -2.39510100

C -2.83283000 -1.79419000 2.21949200

H -3.74196600 -2.04023600 2.79480500

H -2.00388900 -1.70123100 2.93674100

C -2.49925100 -2.92582300 1.26887300

C -2.79055700 -4.25343700 1.59796900

H -3.32860900 -4.48074100 2.52016900

C -2.37631200 -5.26752000 0.73420600

H -2.58715800 -6.31261400 0.97139900

C -1.69276200 -4.92942600 -0.43714500

H -1.35005300 -5.69242000 -1.13780200

C -1.46001700 -3.58088300 -0.69859900

H -0.93474900 -3.26164600 -1.60397100

C 3.02079400 2.50317300 -0.61990500

H 3.49131400 3.05489700 -1.44835000

H 3.58017500 2.74300400 0.29566700

C 2.98655400 0.69358100 -2.26296100

H 3.85439000 1.04712500 -2.84811300

H 2.10214200 1.22416300 -2.64709900

C 2.78161300 -0.78905300 -2.49613700

C 2.87750800 -1.32001900 -3.78779200

H 3.17068800 -0.67781200 -4.62055900

C 2.57733400 -2.66696500 -3.98524400

H 2.63472300 -3.10334400 -4.98487600

C 2.19945400 -3.44836800 -2.88893400

H 1.94920300 -4.50461400 -3.00045700

C 2.15192600 -2.84360000 -1.63478800

H 1.86645000 -3.41409900 -0.74571000

C 4.40730400 0.53342200 -0.29155300

H 5.24353600 1.21489300 -0.51529300

H 4.64152900 -0.42743400 -0.77178000

C 4.28976900 0.29224100 1.18843400

C 5.36532400 0.42932700 2.06671000

H 6.32911500 0.77458700 1.68960000

C 5.18015200 0.12298200 3.41519800

H 6.00705600 0.22513400 4.12116000

C 3.92488200 -0.30813400 3.85096200

H 3.73845400 -0.55545800 4.89686600

C 2.89726500 -0.41182100 2.91882200

H 1.89290600 -0.73720000 3.19650300

O 0.15143700 0.11110700 -1.13358800

O 0.15703900 -0.80031500 1.43824400

H -0.00724400 -0.00005800 1.97438200

H 0.23421800 -0.64167700 -1.73753200

$[Cu_2L(\mu\text{-}OH)_2]^{2+}$ (Broken Symmetry Singlet*)**:**

Electronic energy: -5186.51866685 Hartrees

Gibbs Thermal Correction: 0.572501 Hartrees

Total: -5185.946166 Hartrees

Cu -1.29170700 -0.43308700 0.10011600

Cu 1.58550900 -0.06740400 0.24012100

N -0.98721400 1.79236800 1.66955500

N 0.96073500 2.35371300 0.58480100

N -2.96417400 -0.50418000 1.52569600

N -2.74341400 0.47891400 -1.02462500

N -1.85816600 -2.61141600 0.13355300

N 3.13471300 1.03923900 -0.84604900

N 2.44536600 -1.55061300 -1.44436400

N 3.08560700 -0.11600800 1.62298400

C -2.30268600 1.85280500 1.78142900

C -3.07387100 2.86605000 1.15332900

H -4.15009900 2.90735700 1.32214900

C -2.45093600 3.73126400 0.27919500

H -3.01793500 4.49089800 -0.26300500

C -0.34267300 4.27350000 -0.98132700

H -0.85367500 5.02534600 -1.58701500

C 0.97705000 3.94528100 -1.21425300

H 1.54758200 4.42102200 -2.01319100

C 1.59287800 2.95492200 -0.40537500

C -0.35474400 2.60037600 0.78322400

C -1.06184900 3.58161400 0.02621500

C -2.92768100 0.65699200 2.46658500

H -2.29783300 0.37611300 3.31897400

H -3.94142400 0.87856400 2.83345700

C -4.16846900 -0.48129000 0.66793100

H -5.06142700 -0.19249400 1.24427300

H -4.34549700 -1.50009900 0.29133800

C -3.98798100 0.42222800 -0.51966700

C -5.03400400 1.14708000 -1.09013400

H -6.03346200 1.08546400 -0.65741000

C -4.76744500 1.95971100 -2.19207500

H -5.56726000 2.54560600 -2.64972000

C -3.46362200 2.03072800 -2.68789000

H -3.20934200 2.66967600 -3.53448500

C -2.47366600 1.27197400 -2.07029900

H -1.43202400 1.29177100 -2.39338400

C -2.83067600 -1.79444900 2.22023600

H -3.73944600 -2.04077900 2.79598400

H -2.00140900 -1.70095600 2.93700100

C -2.49705600 -2.92599100 1.26954400

C -2.78655600 -4.25380100 1.59946300

H -3.32333400 -4.48135600 2.52234100

C -2.37212000 -5.26771500 0.73560300

H -2.58155800 -6.31295900 0.97338100

C -1.69023200 -4.92929000 -0.43662600

H -1.34743000 -5.69217400 -1.13735600

C -1.45927800 -3.58059200 -0.69884600

H -0.93533800 -3.26107400 -1.60488500

C 3.01938200 2.50358400 -0.62192100

H 3.48926100 3.05481400 -1.45103600

H 3.57934000 2.74398500 0.29313300

C 2.98446500 0.69290900 -2.26406600

H 3.85183100 1.04641500 -2.84990600

H 2.09959800 1.22294800 -2.64780500

C 2.77985900 -0.78996700 -2.49600700

C 2.87372600 -1.32166600 -3.78749000

H 3.16515600 -0.67984900 -4.62117000

C 2.57369700 -2.66885200 -3.98356600

H 2.62948100 -3.10583200 -4.98302600

C 2.19798500 -3.44969000 -2.88612200

H 1.94786800 -4.50608200 -2.99655300

C 2.15243300 -2.84416900 -1.63226100

H 1.86863400 -3.41419900 -0.74234700

C 4.40709800 0.53463100 -0.29380100

H 5.24258100 1.21671200 -0.51838100

H 4.64167100 -0.42615500 -0.77397800

C 4.29059700 0.29383300 1.18623800

C 5.36660000 0.43086900 2.06393100

H 6.33027100 0.77582500 1.68624900

C 5.18197200 0.12490700 3.41258100

H 6.00922800 0.22707900 4.11812600

C 3.92685600 -0.30594500 3.84906000

H 3.74090700 -0.55306000 4.89509600

C 2.89871900 -0.40965800 2.91751500

H 1.89444800 -0.73494900 3.19554000

O 0.15118100 0.11098500 -1.13373300

O 0.15783400 -0.79970800 1.43885300

H -0.00594600 -0.00052800 1.97633500

H 0.23342100 -0.64022200 -1.73955700

ΔE (Triplet-(Cu(I)+H_2O_2)= -0.061854831 Hartrees= -38.81409202 kcal/mol

ΔE (BS Singlet-(Cu(I)+H_2O_2)= -0.060228611 Hartrees= -37.79363409 kcal/mol

* For the geometry optimization and single point calculation of the BS Singlet state bishydroxide a different input was used:

Geom. Opt.:

#opt freq UB3LYP/def2SVP guess=mix SCF=XQC empiricaldispersion=gd3bj SCRF=(SMD,Solvent=Water)

“Title”

2 1

“Coordinates”

Single Point calculation:

#SP UB3LYP/def2TZVP guess=mix SCF=XQC empiricaldispersion=gd3bj SCRF=(SMD,Solvent=Water)

“Title”

2 1

“Coordinates from geometry optimization”

References

1. Weber, E.; Kohler, H.-J., Benzo Condensed Crown Ethers Containing 1,8-Naphthyridine or 4-Pyridone Units - Synthesis and Complex Formation with Organic Guest Molecules. *J. Prakt. Chem.* **1995,** *337*, 451-455.
2. He, C.; Lippard, S. J., Design and Synthesis of Multidentate Dinucleating Ligands Based on 1,8-Naphthyridine. *Tetrahedron* **2000,** *56*, 8245-8252.
3. Su, X. J.; Gao, M.; Jiao, L.; Liao, R. Z.; Siegbahn, P. E.; Cheng, J. P.; Zhang, M. T., Electrocatalytic water oxidation by a dinuclear copper complex in a neutral aqueous solution. *Angew. Chem. Int. Ed.* **2015,** *54* (16), 4909-4914.
4. He, C.; DuBois, J. L.; Hedman, B.; Hodgson, K. O.; Lippard, S. J., A Short Copper Copper Distance in a (mu-1,2-Peroxo)dicopper(II) Complex Having a 1,8-Naphthyridine Unit as an Additional Bridge. *Angew. Chem. Int. Ed.* **2001,** *40* (8), 1484-1487.
5. Krezel, A.; Bal, W., A formula for correlating pKa values determined in D2O and H2O. *J Inorg Biochem* **2004,** *98* (1), 161-6.
6. Li, L.; Abe, Y.; Kanagawa, K.; Usui, N.; Imai, K.; Mashino, T.; Mochizuki, M.; Miyata, N., Distinguishing the 5,5-dimethyl-1-pyrroline N-oxide (DMPO)-OH radical quenching effect from the hydroxyl radical scavenging effect in the ESR spin-trapping method. *Anal. Chim. Acta* **2004,** *512* (1), 121-124.
7. Frisch, M. J.; Trucks, G. W.; Schlegel, H. B.; Scuseria, G. E.; Robb, M. A.; Cheeseman, J. R.; Scalmani, G.; Barone, V.; Petersson, G. A.; Nakatsuji, H.; Li, X.; Caricato, M.; Marenich, A. V.; Bloino, J.; Janesko, B. G.; Gomperts, R.; Mennucci, B.; Hratchian, H. P.; Ortiz, J. V.; Izmaylov, A. F.; Sonnenberg, J. L.; Williams; Ding, F.; Lipparini, F.; Egidi, F.; Goings, J.; Peng, B.; Petrone, A.; Henderson, T.; Ranasinghe, D.; Zakrzewski, V. G.; Gao, J.; Rega, N.; Zheng, G.; Liang, W.; Hada, M.; Ehara, M.; Toyota, K.; Fukuda, R.; Hasegawa, J.; Ishida, M.; Nakajima, T.; Honda, Y.; Kitao, O.; Nakai, H.; Vreven, T.; Throssell, K.; Montgomery Jr., J. A.; Peralta, J. E.; Ogliaro, F.; Bearpark, M. J.; Heyd, J. J.; Brothers, E. N.; Kudin, K. N.; Staroverov, V. N.; Keith, T. A.; Kobayashi, R.; Normand, J.; Raghavachari, K.; Rendell, A. P.; Burant, J. C.; Iyengar, S. S.; Tomasi, J.; Cossi, M.; Millam, J. M.; Klene, M.; Adamo, C.; Cammi, R.; Ochterski, J. W.; Martin, R. L.; Morokuma, K.; Farkas, O.; Foresman, J. B.; Fox, D. J. *Gaussian 16 Rev. C.01*, Wallingford, CT, 2016.
8. Becke, A. D., Density-functional thermochemistry. III. The role of exact exchange. *J. Chem. Phys.* **1993,** *98* (7), 5648-5652.
9. Lee, C.; Yang, W.; Parr, R. G., Development of the Colle-Salvetti correlation-energy formula into a functional of the electron density. *Physical Review B* **1988,** *37* (2), 785-789.
10. Vosko, S. H.; Wilk, L.; Nusair, M., Accurate spin-dependent electron liquid correlation energies for local spin density calculations: a critical analysis. *Can. J. Phys.* **1980,** *58*, 1200-1211.
11. Stephens, P. J.; Devlin, F. J.; Chabalowski, C. F.; Frisch, M. J., Ab Initio Calculation of Vibrational Absorption and Circular Dichroism Spectra Using Density Functional Force Fields. *J. Phys. Chem.* **1994,** *98* (45), 11623-11627.
12. Weigend, F.; Ahlrichs, R., Balanced basis sets of split valence, triple zeta valence and quadruple zeta valence quality for H to Rn: Design and assessment of accuracy. *Phys Chem Chem Phys* **2005,** *7* (18), 3297-305.
13. Weigend, F., Accurate Coulomb-fitting basis sets for H to Rn. *Phys Chem Chem Phys* **2006,** *8* (9), 1057-65.
14. Marenich, A. V.; Cramer, C. J.; Truhlar, D. G., Universal Solvation Model Based on Solute Electron Density and on a Continuum Model of the Solvent Defined by the Bulk Dielectric Constant and Atomic Surface Tension. *J. Phys. Chem. B* **2009,** *113* (18), 6378–6396.

15. Grimme, S.; Antony, J.; Ehrlich, S.; Krieg, H., A consistent and accurate ab initio parametrization of density functional dispersion correction (DFT-D) for the 94 elements H-Pu. *J Chem Phys* **2010,** *132* (15), 154104.
16. Grimme, S.; Ehrlich, S.; Goerigk, L., Effect of the damping function in dispersion corrected density functional theory. *J Comput Chem* **2011,** *32* (7), 1456-1465.
